# Recombination Does Not Hinder Formation or Detection of Ecological Species of *Synechococcus* Inhabiting a Hot Spring Cyanobacterial Mat

**DOI:** 10.3389/fmicb.2015.01540

**Published:** 2016-01-14

**Authors:** Melanie C. Melendrez, Eric D. Becraft, Jason M. Wood, Millie T. Olsen, Donald A. Bryant, John F. Heidelberg, Douglas B. Rusch, Frederick M. Cohan, David M. Ward

**Affiliations:** ^1^Department of Land Resources and Environmental Science, Montana State UniversityBozeman, MT, USA; ^2^Department of Biochemistry and Molecular Biology, Pennsylvania State UniversityUniversity Park, PA, USA; ^3^Department of Biological Sciences, College of Letters, Arts and Sciences, University of Southern CaliforniaLos Angeles, CA, USA; ^4^Informatics Group, J. Craig Venter InstituteRockville, MD, USA; ^5^Department of Biology, Wesleyan UniversityMiddletown, CT, USA

**Keywords:** population genetics, speciation, *Synechococcus*, cyanobacteria, multi-locus sequence typing, Ecotype Simulation, ecotype, recombination

## Abstract

Recent studies of bacterial speciation have claimed to support the biological species concept—that reduced recombination is required for bacterial populations to diverge into species. This conclusion has been reached from the discovery that ecologically distinct clades show lower rates of recombination than that which occurs among closest relatives. However, these previous studies did not attempt to determine whether the more-rapidly recombining close relatives within the clades studied may also have diversified ecologically, without benefit of sexual isolation. Here we have measured the impact of recombination on ecological diversification within and between two ecologically distinct clades (A and B') of *Synechococcus* in a hot spring microbial mat in Yellowstone National Park, using a cultivation-free, multi-locus approach. Bacterial artificial chromosome (BAC) libraries were constructed from mat samples collected at 60°C and 65°C. Analysis of multiple linked loci near *Synechococcus* 16S rRNA genes showed little evidence of recombination between the A and B' lineages, but a record of recombination was apparent within each lineage. Recombination and mutation rates within each lineage were of similar magnitude, but recombination had a somewhat greater impact on sequence diversity than mutation, as also seen in many other bacteria and archaea. Despite recombination within the A and B' lineages, there was evidence of ecological diversification within each lineage. The algorithm Ecotype Simulation identified sequence clusters consistent with ecologically distinct populations (ecotypes), and several hypothesized ecotypes were distinct in their habitat associations and in their adaptations to different microenvironments. We conclude that sexual isolation is more likely to follow ecological divergence than to precede it. Thus, an ecology-based model of speciation appears more appropriate than the biological species concept for bacterial and archaeal diversification.

## Introduction

For much of the last century, theories of the origin of species have revolved around genetic exchange (Mayr, 1942, 1963; Dobzhansky, 1951; Coyne and Orr, 2004). In highly sexual organisms such as animals and plants, genetic exchange is frequently seen as a cohesive force holding together the individuals within a species population, thus preventing divergence into irreversibly separate lineages (Cohan, 2013). The role of genetic exchange in bacteria and archaea, where recombination is not connected to reproduction (Levin and Bergstrom, 2000), has been vigorously debated. Recombination has been viewed by some as a rampant process that can erode the distinctness of nascent species (Doolittle and Zhaxybayeva, 2009); moreover, recombination is seen as a cohesive force binding a species together, as in the case of sexual species. Speciation theories based on Mayr's Biological Species Concept see the breaking of recombination between populations as a critical step in population divergence; in this view, populations can diverge ecologically and irreversibly only after their genetic exchange is blocked (Coyne and Orr, 2004).

On the other hand, theories of ecological speciation hold that ecological divergence is the critical step in the origin of diversity, and that recombination plays only a minor role in the subsequent fate of newly split, ecologically distinct populations (Van Valen, 1976; Mallet, 2008; Schluter, 2009). According to a theory developed long ago by Haldane, rare introduction of genes from one population to another cannot reverse the adaptive divergence between populations (Haldane, 1932). This is because natural selection against niche-specifying genes from other populations can limit these foreign genes to negligible frequencies, especially if the rate of recombination between populations is very low. The equilibrium frequency of a recombinant niche-specifying gene is equal to the rate of recombination between populations (*c*_*b*_) divided by the intensity of selection (*s*) against the gene (Mallet, 2008; Wiedenbeck and Cohan, 2011). The equilibrium frequency of recombinant genes is thus extremely low, as recombination rates are not known to exceed the mutation rate by more than a factor of 10 (Vos and Didelot, 2009), and mutation rates are known to be very low in nearly all bacteria and archaea studied (about 10^−6^ per gene per generation; Drake, 2009). It can therefore be inferred that recombination rates are much too low to reverse adaptive divergence of microbial populations, and that recombination is unlikely to prevent ecological divergence among the most closely related bacteria and archaea (Wiedenbeck and Cohan, 2011).

The conflict between recombination-based and ecology-based theories of speciation has motivated several recent studies on the role of recombination in the evolution of bacterial and archaeal species. In a study of coexisting strains of *Sulfolobus islandicus* isolated from the same hot spring, Cadillo-Quiroz and colleagues found depressed rates of recombination between two major clades formed by these organisms compared to recombination within either clade (Cadillo-Quiroz et al., 2012). Inter-clade recombination was taken to be insufficient to prevent the divergence of the clades into easily distinguishable clusters, and the authors claimed this as support for the Biological Species Concept in archaeal speciation. Ellegaard et al. came to a similar conclusion based on comparison of genomes derived from closely related *Wolbachia* strains (97–98% 16S rRNA identity) isolated from the same host fly (Ellegaard et al., 2013). Shapiro et al. obtained similar results for closely related clades of marine *Vibrio cyclitrophicus* strains that may be ecologically distinct, based on their isolation from particles of different sizes (Shapiro et al., 2012). They proposed a model in which ecological specialization, based on horizontal genetic transfer, leads to occupancy of different habitats; this causes a decrease in recombination between the ecologically distinct populations, allowing the ecological populations to further diverge (Shapiro et al., 2012; see also Retchless and Lawrence, 2010; Polz et al., 2013). In all these studies the reduced recombination between the clades under investigation was taken as evidence that recombination must be lowered to allow irreversible divergence among close relatives into stably coexisting, ecologically distinct populations. However, these studies did not address whether ecological divergence may have also occurred *within* each clade, where recombination rates are higher.

Here we test whether ecological divergence can occur among organisms so closely related that recombination between them is not reduced. Our model system is the set of unicellular cyanobacteria (*Synechococcus*) inhabiting the microbial mats of Mushroom Spring, Yellowstone National Park. This hypothesis emerged from a series of studies based on variation found in single genetic loci (Ferris and Ward, 1997; Ramsing et al., 2000; Ferris et al., 2003; Melendrez et al., 2011). First, closely related 16S rRNA genotypes were distributed differently along the effluent channel (genotypes A,” A', A, B', and B along a temperature gradient from ~72–74°C to ~50°C Ferris and Ward, 1997) and with depth in the photic zone (Ramsing et al., 2000), suggesting differential adaptations to temperature and light that were later confirmed by studying isolates with genotypes representative of *in situ* populations (Allewalt et al., 2006). Second, analyses based on the internal transcribed spacer separating 16S and 23S rRNA genes revealed that 16S rRNA sequence variation was insufficient to detect all ecologically distinct populations (Ferris et al., 2003; Ward et al., 2006), limiting the utility of 16S rRNA-based methods for detecting closely related ecotypes (e.g., Eren et al., 2013). Third, analyses of several more rapidly evolving protein-encoding genes revealed numerous closely related and ecologically distinct populations, the number of which depended on the depth of coverage and on the degree of sequence divergence of the gene used (Becraft et al., 2011; Melendrez et al., 2011). It is clearly important to appreciate that the amount of molecular divergence between these closely related species is very small (see Discussion).

Our aim in these studies has been to discover the fundamental “ecotypes” within the A and B' clades (originally demarcated as 16S rRNA clusters), where we define an *ecotype* as a group of organisms that is ecologically distinct from other ecotypes and whose members are ecologically interchangeable (Kopac and Cohan, 2011). More specifically, different ecotypes are expected to coexist indefinitely owing to their ecological distinctness, while lineages within an ecotype are too similar to be able to coexist indefinitely (Kopac et al., 2014). In our recent work we hypothesized the demarcations of putative ecotypes (PEs) from sequence clusters using a theory-based algorithm (Ecotype Simulation, based on the Stable Ecotype Model of species and speciation, Kopac et al., 2014; Cohan, 2016), which simulates the evolutionary dynamics of ecotype formation and the forces that purge sequence diversity within ecotypes (Koeppel et al., 2008). Unlike other studies that have used traditional or intuitive sequence cutoffs to define sequence clusters of interest (e.g., “main cloud” roughly equivalent to 3% 16S rRNA cutoff, see Rosen et al., 2015), we have used this dynamic approach to let the natural variation, itself, reveal the clusters that most likely fit the dynamic properties of species, given the resolution allowed by the sequence data. In recent pyrosequencing analyses of a gene encoding an essential photosynthesis protein (PsaA), Ecotype Simulation demarcated 18 B'-like, 14 A-like, and 5 A'-like *Synechococcus* PEs (Becraft et al., 2015). Importantly, these analyses demonstrated the ecological distinctness of the PEs: (i) the most abundant PEs had unique environmental distributions, (ii) the PEs showed unique gene expression patterns, (iii) cultivated strains with identical 16S rRNA sequences (see Nowack et al., 2015) from PEs with different depth distributions showed different light adaptations and acclimation responses (Nowack et al., 2015), and (iv) comparative genomic analyses of these strains are beginning to reveal the genetic mechanisms underlying these adaptations (Olsen et al., 2015). Moreover, the membership within PEs was shown to be ecologically interchangeable: (v) individuals predicted to belong to the same PE were distributed similarly along ecological gradients and (vi) the membership within a PE showed coordinated responses to environmental change (Becraft et al., 2015; Olsen et al., 2015).

Recently, Rosen et al. (2015) presented a view of the same Mushroom Spring *Synechococcus* populations that suggests recombination prevents the formation of such PEs. These authors pyrosequenced numerous PCR-amplified genes to study the genetic diversity within the A and B' lineages of the present model system (that is, the A and B' lineages of *Synechococcus* in Mushroom Spring) Although, Rosen et al. described their study as a multi-locus sequence analysis, any given organism was sampled for only one locus (i.e., multiple loci were unlinked in these analyses). Rosen et al. (2015) inferred from patterning of single-nucleotide polymorphisms (SNPs) in individual amplicons that recombination and mutation occur at similar rates within the sampled population. Viewing recombination to be “ubiquitous” and, based on comparison of neutral drift and sexual recombination models, they inferred that their “results were inconsistent with the presence of multiple ecotypes” within the A and B' lineages and conjectured that the A and B' lineages of *Synechococcus* were each behaving “as effectively [a] quasisexual species… occupying a broad environmental niche.” However, as noted above we previously showed these lineages to each contain multiple, ecologically distinct populations (Becraft et al., 2015; Nowack et al., 2015; Olsen et al., 2015). Clearly, these *Synechococcus* populations provide a test case for understanding the roles of recombination and ecological specialization in speciation within these lineages. Here we will demonstrate in a true multilocus analysis that each lineage has diversified into ecologically distinct populations in spite of recombination between populations.

Single-locus analyses may suffer from the effects of recombination, such that the evolutionary history of the gene may deviate from that of the organisms (Feil et al., 2001, 2004). Thus, we developed a novel multi-locus sequence analysis (MLSA) approach for cultivation-free sampling and analysis of population structure. MLSA provides a buffer against the effects of recombination in any single gene, increases molecular resolution, and allows detection of recombination events that involve both whole genes and parts of genes because the genes investigated are known to be linked on the same genome. MLSA was developed for population genetics studies of cultivated pathogenic isolates (Maiden et al., 1998; Feil et al., 2000a; Salerno et al., 2007; Vitorino et al., 2008; Cesarini et al., 2009) and has also been applied to nonpathogenic isolates (Papke et al., 2004, 2007; Whitaker et al., 2005; Koeppel et al., 2008; Mazard et al., 2012). Furthermore, the development of methods that permit access to large segments of genomes has extended its application to uncultivated organisms (Kashtan et al., 2014). Our cultivation-independent MLSA approach was based on the use of bacterial artificial chromosome (BAC) libraries (Shizuya et al., 1992; Tao et al., 2002; Liles et al., 2008) to sample multiple genes from individual genomes of *Synechococcus* inhabiting the microbial mat of Mushroom Spring.

BACs were derived from libraries constructed from DNA that was extracted from samples collected at either a 60°C site or a 65°C site. Consequently, like the other studies mentioned above, our focus populations have diverged into two major lineages (i.e., the A and B' lineages of *Synechococcus*, defined by 16S rRNA sequence variants A and B', see above) inferred to rarely recombine between lineages and known to be ecologically distinct (Ward et al., 1998, 2006; Klatt et al., 2011). BACs were screened to identify clones containing a *Synechococcus* A/B lineage-specific 16S rRNA region. Multiple loci were selected and PCR-amplified from these BAC clones and the sequence data obtained were analyzed using Ecotype Simulation of both concatenated MLSA and single-gene phylogenies, and alternatively, using eBURST analysis, which has routinely been used in MLSA studies to demarcate variants into clusters (see Methods; Feil et al., 2004).

Ecotype Simulation and eBURST use very different approaches to infer populations from sequence variation. Ecotype Simulation is a theory-based algorithm demarcating clades that are consistent with the dynamics of ecotype formation and purging of diversity within ecotypes; eBURST clusters variants on the basis of a pre-determined cutoff—that members of a cluster must be identical at all or all but one of the loci studied; however, concatenated MLSA sequences have been used in phylogenetic analyses (independent from eBURST analyses) to assist in demarcating species clusters (Maiden et al., 1998; Hanage et al., 2005). In previous analyses of this type, suspected recombinant sequences have simply been removed from Ecotype Simulation analyses, on the assumption that these recombinant sequences do not reflect the history of the organism at the locus undergoing recombination and, if not removed, might lead to overestimation of PEs (Koeppel et al., 2008). In this study, however, we address the ability of Ecotype Simulation to predict PEs when recombined sequences are included.

The extent of recombination was assessed in single-gene and concatenated MLSA alignments using programs designed to detect recombination events and phylogenetic incongruency (also assessed by visual comparisons), and by SNP pattern analyses. The ecological distinctness of PEs, predicted either by MLSA or single-locus analysis, was determined through association with the temperature (60° or 65°) from which the BACs were obtained. We also tested for finer-scale habitat associations by tracking the distributions of genetic variants of an MLSA locus using pyrosequencing of DNA from samples collected over a range of vertical microenvironments in the 63–65°C mat (Becraft et al., 2015). In some cases genomic sequences were available for isolates representative of PEs demarcated in MLSA analyses (Olsen et al., 2015). This permitted comparison of distribution of the MLSA locus with *psaA* distributions that had previously been determined on the same samples (Becraft et al., 2011).

We will demonstrate not only that recombination has occurred more frequently within than between the A and B' lineages, but that it has neither prevented ecological diversification within each of these lineages nor our ability to detect ecotypes. We suggest that an ecology-based model of speciation is more appropriate than the biological species concept for bacterial speciation in hot spring *Synechococcus*, and possibly many other bacteria and archaea in diverse environments.

## Results

### BAC clone libraries

**Figure 1:**
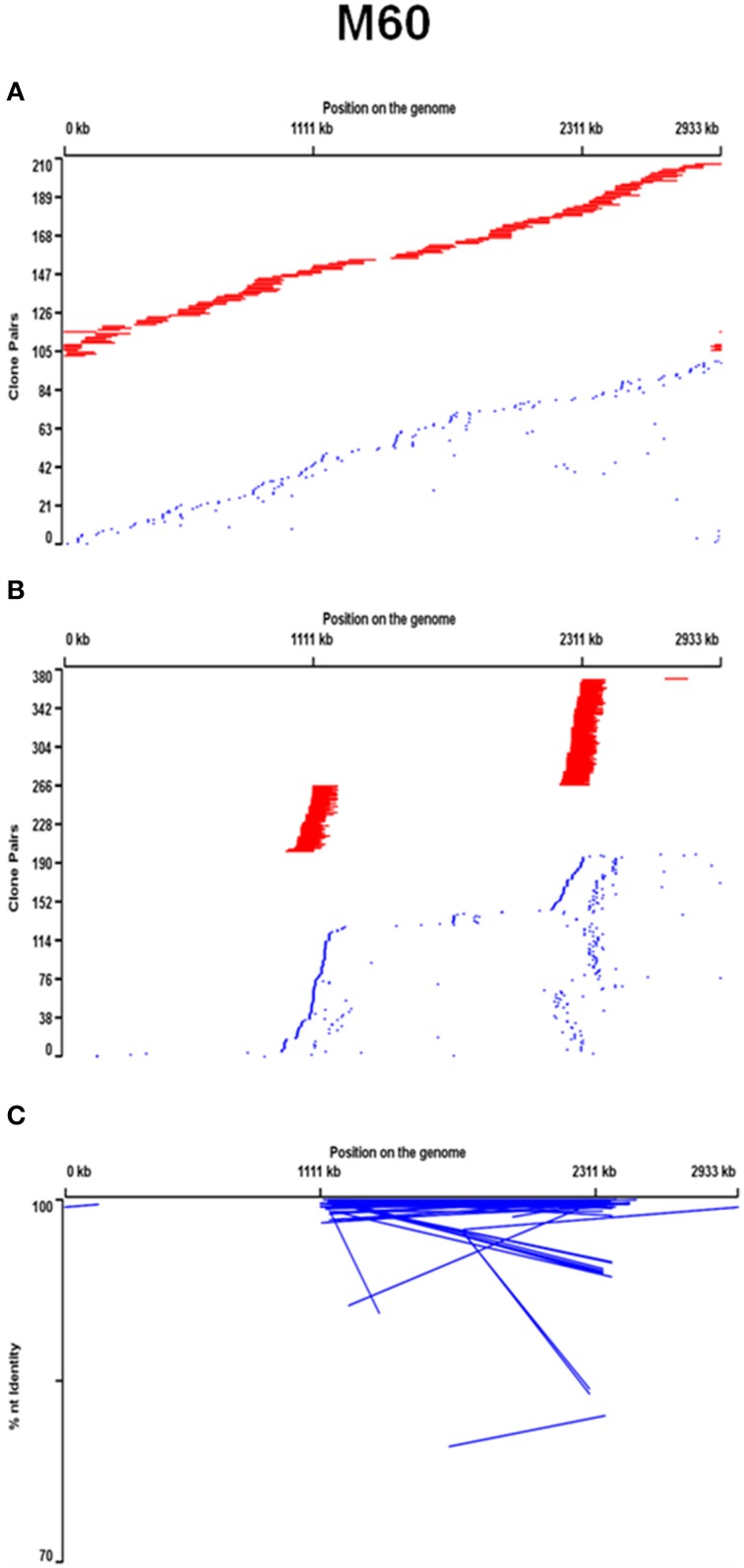
**Positional analysis relative to the *Synechococcus* strain A genome of jointly recruited syntenous (red line joins end sequences) and jointly recruited non-syntenous (blue) end sequences of 60°C BAC clones that are (A) recruited by this genome, and (B) contain an A-like *Synechococcus* 16S rRNA region, and (C) have anti-normal long end-sequence mate pairs**. In **(C)** percent nucleotide identity of recruited sequences with genomic homologs is also plotted and lines connect mate-pairs.

**Figure 2:**
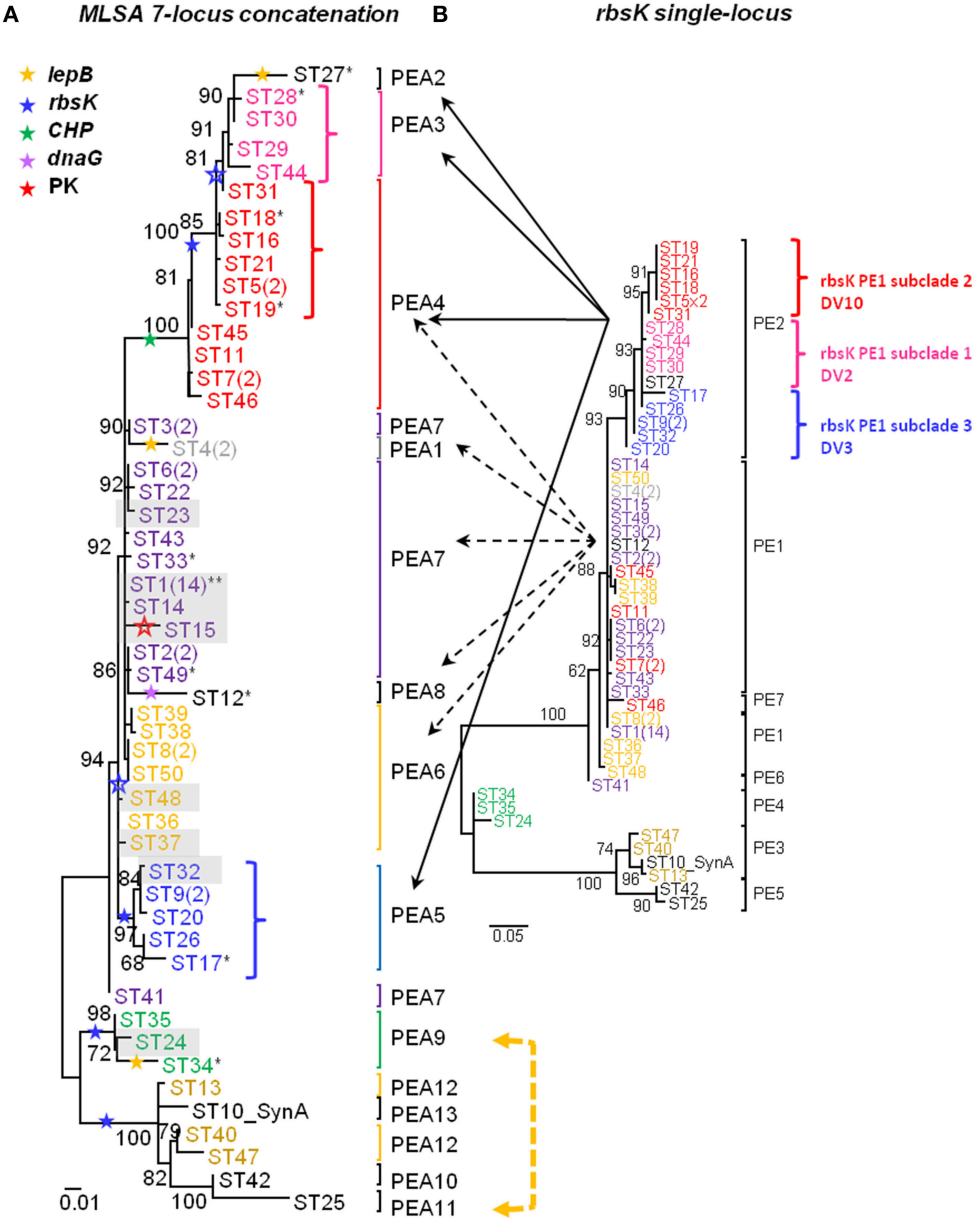
**A-like *Synechococcus* phylogenies based on maximum likelihood analysis of (A) 7 concatenated BAC loci and (B) BAC-associated *rbsK* sequences**. Putative ecotypes (PEs) demarcated by Ecotype Simulation are indicated by brackets or vertical bars adjacent to each tree. STs within PEs are colored according to the MLSA phylogeny shown in A and colors are maintained in both phylogenies (color correspondence also maintained in Supplemental Data Presentation Figure 14). The number of variants belonging to dominant and subdominant sequence types (STs) is indicated in parentheses. Stars, colored coded according to gene (see inset star legend), demarcate recombination events interpreted from SNP patterns confirmed (closed stars) or not confirmed (open stars) by Clonal Frame analysis. Clade splitting events between *rbsK* and the concatenated phylogeny, where grouped variants within *rbsK* are split apart into separate PEs in the concatenated phylogeny, are indicated by dashed (clade splitting event of *rbsK* PE1) and solid arrows (clade splitting event of *rbsK* PE2). Non-syntenous STs are shaded in gray and STs that contained a combination of sequences that were syntenous and non-syntenous are indicated by an asterisk; syntenous STs are not shaded and are not annotated by an asterisk. Shared SNP pattern between STs of two distinct PEs indicated by a bidirectional arrow colored according to gene (yellow = *lepB*). Bootstrap values are provided for major nodes. Reference genome indicated by “SynA”; Genbank accession number CP000239.

**Figure 3:**
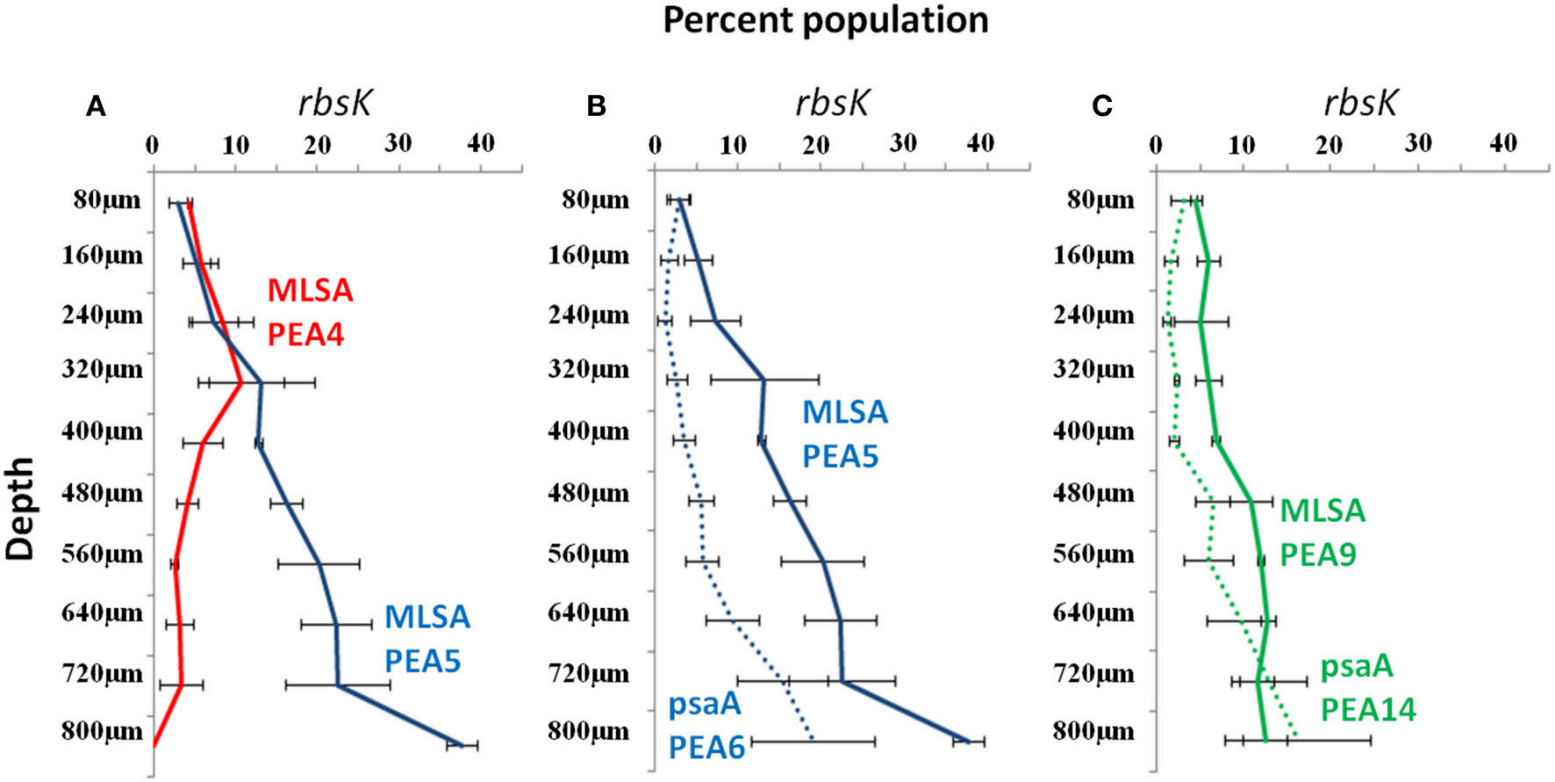
***rbsK* vertical distributions in the 63–65°C Mushroom Spring microbial mat of dominant *rbsK* and *psaA* variants associated with *Synechococcus* putative ecotypes (PE) demarcated from multi-locus sequence analyses (MLSA) by Ecotype Simulation, shown as colored solid lines**. **(A)** *rbsK* distributions of two MLSA PEs that are demarcated as a single PE in *rbsK* analysis. **(B,C)** *rbsK* (solid line) and *psaA* (dotted line) distributions of two MLSA PEs connected through genomes of representative isolates. Bars represent standard error (*n* = 3). PE colors correspond across panels.

Metagenomic libraries constructed from the 60° and 65° mat samples from the Mushroom Spring effluent channel contained 304,128 and 64,512 BAC clones with average insert sizes of 90 and 120 kb, respectively (see Supplemental Data Sheet Section I). BLAST analyses of paired-end sequences of 9216 randomly selected clones from each BAC library showed that the BAC libraries contained the same predominant taxonomic composition as small-insert metagenomes previously produced from the same DNA (Klatt et al., 2011; also see Supplemental Data Sheet Section II, Supplemental Data Tables 3, 4, and Supplemental Data Presentation Figure 2). While the BACs yielded poorer recovery of *Synechococcus* genes than the small-insert metagenomes, they provided broad coverage of *Synechococcus* strain JA-3-3Ab [accession: CP000239.1] and JA-2-3B'a(2-13) [accession: CP000240.1] reference genomes, which are representative of A-like and B'-like *Synechococcus* 16S rRNA lineages (Bhaya et al., 2007; Figure 1A, Supplemental Data Presentation Figure 3). BAC clones containing a *Synechococcus* strain A-like or B'-like 16S rRNA sequence were identified by oligonucleotide probe screening and sequence analysis (Supplemental Data Sheet Section I). Mapping the distribution of the paired-end sequences of each BAC clone relative to reference genomes suggested nearly equal and random coverage of the two unlinked 16S rRNA loci of these genomes (Figure 1B, Supplemental Data Presentation Figure 3). Interestingly, many *Synechococcus* A-like or B'-like BACs containing a 16S rRNA sequence were non-syntenous when compared with the respective genome sequence. Syntenous sequences are considered here to be those whose mate pairs were of the same orientation as the reference genome and were separated by a distance that was similar to the size of the DNA fragments used to construct the metagenomic library. Non-syntenous sequences are considered here to be those whose mate pairs deviated from the insert length and/or orientation of the reference genome (See Supplemental Data Sheet Section II and reference, Klatt et al., 2011). Such cases were revealed by the paired-end sequences of the same clone matching regions near both 16S rRNA loci, as opposed to one. The percentage of normal- and anti-normal long orientations of paired-end sequences in these non-syntenous clones was greater than in randomly sampled BACs (Figure 1C, Supplemental Data Sheet Section II, Supplemental Data Table 5, and Supplemental Data Presentation Figure 3). This suggested that non-syntenous BACs may have been associated with genomic inversions (Rusch et al., 2007).

### MLSA databases

BACs containing A-like and B'-like *Synechococcus* 16S rRNA genes were screened by PCR amplification of loci targeted for MLSA analysis (Tables 1, 2; Supplemental Data Tables 1, 2). MLSA loci were selected on the basis of their being (i) single-copy genes, (ii) under neutral or purifying selection, that (iii) represented a range of degrees of divergences and distances from the 16S rRNA locus and (iv) were not adjacent to potential mobile elements (see Supplemental Data Sheet Sections I, III).

**Table 1:** **Results from recombination and mutation rate and ratio analyses for A-like *Synechococcus* BACs**.

**Locus (No. of Loci)**	**No. of STs in Analysis**	**No. of nts^b^**	**No. of Segregating Sites**	**LDHat**					**ClonalFrame**			
				**θ^c^**	**R^d^ |R^k^ |R^l^**	**Ave PWD^i^**	**Var PWD^j^**	**R/θ^e^**	**θ^c^(CI)**	**R^d^ (CI)**	**ρ∕θ^e^(CI)**	**r/m^f^(CI)**
7^m^	50	4008	183	40.86	41|13|1.6	33.14	580.03	0.04–1.0	33.94	20.365 (12.92–30.29)	0.600 (0.38–0.892)	2.596 (1.57–4.01)
5^a^^,^ ^m^	69	2760	222	45.67	44|16|2.8	39.19	706.5	0.06–0.96	42.59	24.76 (16.75–34.65)	0.581 (0.39–0.81)	3.396 (2.21–4.92)
*rbsK*	^h^	520	119	24.48	5|9|0	23.24	509.7	0–0.368	^h^	^h^	^h^	^h^
*PK^*m*^*	^h^	584	30	6.17	11|1|0	3.63	18.85	0–1.7	^h^	^h^	^h^	^h^
*hisF*	^h^	429	0	^g^	^g^			^g^	^g^	^g^	^g^	^g^
*lepB^*m*^*	^h^	420	30	6.17	0|2|0	2.11	18.58	0–0.324	^h^	^h^	^h^	^h^
*CHP^*m*^*	^h^	629	28	5.76	7|1|0	9.13	93.59	0–1.2	^h^	^h^	^h^	^h^
*aroA*	^h^	607	15	3.09	0|0|0	1.10	2.67	0.0	^h^	^h^	^h^	^h^
*dnaG^*m*^*	^h^	547	26	5.80	0|0|0	1.49	20.48	0.0	^h^	^h^	^h^	^h^

CI: 95% credibility interval.

a rbsK, CHP, PK, lepB, aroA.

b Sum of all single gene lengths (number of nucleotides) and analysis pertains to a 73-sequence (145 sequence de-duplicated) 5-locus alignment except for hisF and dnaG which pertain to 7-locus alignment.

c Watterson's theta.

d Maximum at 4Ner(region); composite likelihood method (LDHat; pairwise) finite sites model (McVean et al., 2002, 2004).

e R/θ from LDHat is equivalent to the ρ/θ measure from Clonal Frame and define the ratio of the rates of recombination (R or ρ) to mutation (θ) illustrating how often recombination occurs relative to mutation (McVean et al., 2002, 2004; Didelot and Falush, 2007; Didelot and Maiden, 2010). For LDHat-pairwise analysis, R/θ range includes calculations from composite-likelihood, Rmin and Wakeleys moment method (McVean et al., 2002, 2004).

f Ratio of the probabilities that a given site is altered through recombination (r) and mutation (m) illustrating how important the effect of recombination was in the diversification of the samples relative to mutation (Didelot and Falush, 2007; Didelot and Maiden, 2010).

g There were no segregating sites in the sequence dataset.

h Clonal Frame analysis is designed for concatenated multi-locus alignments (Didelot and Falush, 2007; Didelot and Maiden, 2010).

i avePWD: average pairwise distance (LDHat pairwise).

j varPWD: variance in pairwise distance (LDHat pairwise).

k Rmin value (LDHat pairwise); minimum number of recombination events describing evidence for recombination in region (McVean et al., 2002, 2004) infinite sites model.

l Population scaled 4Ner (recombination estimate) by Wakeley Moment method (McVean et al., 2002, 2004).

m The look up table for LDHat pairwise not generated from data but from existing lkgen table provided in LDHat distribution (lk_n100_t0.01; https://github.com/auton1/LDhat).

**Table 2:** **Results from recombination and mutation rate and ratio analyses for B'-like *Synechococcus* BACs**.

**Locus (No. of Loci)–PE**	**No. of STs in Analysis**	**No. of Nts**	**No. of Segregating Sites**	**LDHat**					**ClonalFrame**			
				**θ^a^**	**R^b^ |R^h^ |R^i^**	**Ave PWD^f^**	**Var PWD^g^**	**R/θ^c^**	**θ^a^**	**R^b^(CI)**	**ρ∕θ^d^(CI)**	**r/m^e^(CI)**
4^j^	51	2448	361	80.24	85|0|22.3	66.14	905.36	0–1.06	63.34	23.53 (15.18–33.89)	0.371 (0.24–0.535)	2.42 (1.58–3.44)
*aroA*^j^	^e^	550	101	22.45	0|13|0	14.42	219.48	0–0.579	^e^	^e^	^e^	^e^
*rbsk*	^e^	535	122	27.12	15|15|2.9	34.05	539.83	0.44–0.55	^e^	^e^	^e^	^e^
*pcrA*^j^	^e^	628	62	13.78	38|11|0.5	11.06	75.06	0.36–2.8	^e^	^e^	^e^	^e^
16S rRNA/ITS^j^	^e^	733	76	16.89	15|0|0	6.725	43.46	0–0.889	^e^	^e^	^e^	^e^

CI: 95% credibility interval.

a Watterson's theta.

b Maximum at 4Ner(region); composite likelihood method (LDHat; pairwise) finite sites model (McVean et al., 2002, 2004).

c R/θ from LDHat is equivalent to the ρ/θ measure from Clonal Frame and define the ratio of the rates of recombination (R or ρ) to mutation (θ) illustrating how often recombination occurs relative to mutation (McVean et al., 2002, 2004; Didelot and Falush, 2007; Didelot and Maiden, 2010). For LDHat-pairwise analysis, R/θ range includes calculations from composite-likelihood, Rmin and Wakeleys moment method (McVean et al., 2002, 2004).

d Ratio of the probabilities that a given site is altered through recombination (r) and mutation (m) illustrating how important the effect of recombination was in the diversification of the samples relative to mutation (Didelot and Falush, 2007; Didelot and Maiden, 2010).

e Clonal Frame analysis is designed for concatenated multi-locus alignments (Didelot and Falush, 2007; Didelot and Maiden, 2010).

f avePWD: average pairwise distance (LDHat pairwise).

g varPWD: variance in pairwise distance (LDHat pairwise).

h Rmin value (LDHat pairwise); minimum number of recombination events describing evidence for recombination in region (McVean et al., 2002, 2004) infinite sites model.

i Population scaled 4Ner (recombination estimate) by Wakeley Moment method (McVean et al., 2002, 2004).

j The look up table for LDHat pairwise ***not*** generated from data but from existing lkgen table provided in LDHat distribution (lk_n100_t0.01; https://github.com/auton1/LDhat).

**Figure 4:**
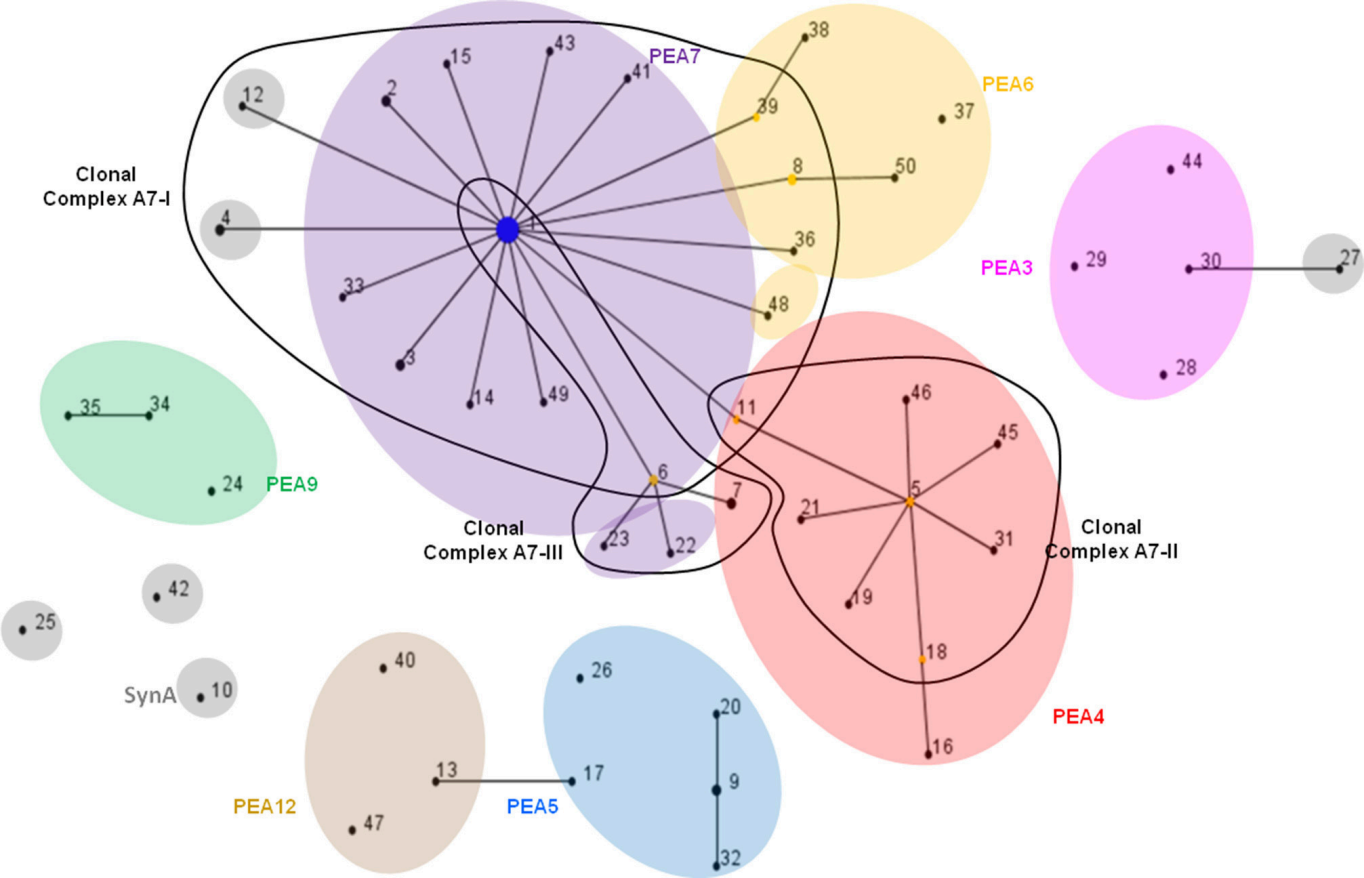
**eBURST population snapshot of A-like *Synechococcus* BACs for the 7-locus analysis shown in Figure 2A.** Clonal complexes enclosed by solid black lines. PE demarcation from Ecotype Simulation analysis are overlaid, using different colors corresponding to Figure 2A to represent distinct PEs. STs are represented by numbers and those colored in gray belong to PEs demarcated from a single sequence with a unique ST. Reference genome indicated by “SynA” next to ST10.

The distributions of MLSA loci among the 267 A-like and 237 B'-like BACs containing the targeted 16S rRNA locus and at least one of the other MLSA loci are shown in Supplemental Data Sheet Section III and Supplemental Data Tables 6, 7. The majority of BACs (94 and 66% of *Synechococcus* A- and B'-like BACs, respectively) exhibited locus combinations consistent with the gene order of the reference genomes. The number of BACs positive for various loci decreased as the distance separating them from the 16S rRNA locus increased, providing additional evidence of genome rearrangements in these lineages (Supplemental Data Sheet Section III and Supplemental Data Presentation Figure 4). There was no obvious pattern of decaying synteny with increasing phylogenetic divergence (see end of legends in Figure 2 and Supplemental Data Presentation Figure 10 identifying syntenous and non-syntenous clones in the dataset).There were 71 *Synechococcus* A-like BACs that contained the total set of 7 selected loci (*rbsK, PK* [locus tag CYA_2262], *hisF, lepB, CHP* [locus tag CYA_2291], *aroA* and *dnaG*) in addition to the 16S rRNA/16S-23S rRNA internal transcribed spacer (ITS) region (Supplemental Data Table 6). However, only two *Synechococcus* B'-like BACs contained 7 of the targeted MSLA loci (Supplemental Data Table 7). In order to obtain a comparable sampling of *Synechococcus* B'-like and A-like BAC clones, the number of loci for the *Synechococcus* B'-like population was reduced to three protein-encoding loci (*rbsK, aroA* and *pcrA*) plus the 16S rRNA/16S-23S rRNA ITS sequence. The amount of nucleotide divergence at the 16S rRNA locus within A-like and B'-like BACs averaged 0.31% (±0.036% SE) and 0.71% (±0.064% SE), respectively, much less than that between BACS of the different lineages, which averaged 2.73% (±0.024% SE), which was somewhat lower than the 3.6% divergence reported for the *Synechococcus* strain A and B' genomes (Bhaya et al., 2007). We compared the *rbsK* sequences of A-like and B'-like BACs to *rbsK* sequences recovered by direct PCR amplification from the same mat samples and found that the BAC library sequences sampled broadly across B'-like and A-like lineages (Supplemental Data Sheet Section IV and Supplemental Data Presentation Figures 6, 7). All multi-locus sequence datasets were assembled into sequence types (STs) comprised of individual BACs with identical sequences at all loci (Supplemental Data Sheet Section V and Supplemental Data Tables 8, 9).

### Recombination and mutation within and between A and B' *Synechococcus* lineages

Evidence of recombination within the A- and B'-lineages was readily detected by various analyses of both single genes and multiple loci. The ability to detect recombination events was influenced by the number and length of sequences analyzed and by the number of genes included in the analysis. Furthermore, different methods detected different events. For instance, as shown in Table 3, RDP4 analysis of 7 loci in 71 A-like BACs detected 11 unique events. Clonal Frame detected 7 of the same events, and manual inspection of SNPs (using the definition from (Feil et al., 2000a), where >1 SNP within any single locus is indicative of recombination) suggested 24 additional unique putative events. Hence, 11–35 unique events were detected. However, in an analysis in which we more than doubled the number of sequences by removing 2 of the 7 genes (see below), RDP4 detected only 5 events; Clonal Frame detected 1 additional event and SNP analysis suggested 2 more events (range 6–8 events). Similarly, in an analysis of 4 genes in 72 B'-like BACs RDP4 detected 4 events, Clonal Frame detected an additional 9 events and SNP inspection suggested 11 additional unique events (range 13–24 events).

**Table 3:** **The number of unique (u) and overlapping (ovl; supported by multiple methods) recombination events recorded by RDP4, Clonal Frame (CF), and single nucleotide polymorphism (SNP) analysis of MLSA datasets**.

**Organism**	**Datastet**	**No. Sequences**	RDP4u^*^^a^^,^^b^	**CFu**	**CFovl**	**SNPu**	**SNPovl**	**All Unique^e^**
*Synechoccocus* A-like BACs	MLSA7^d^	71	11	0^a^	7^a^	24^a^	7^a^	35
	MLSA5	49	5	0^a^	0^a^	0^a^	0^a^	5
	MLSA5	145	5	1^a^	8^a^	2^a^	6^a^	8
*Synechococcus* B'-like BACs	MLSA4	72	4	9^b^^,^ ^c^	19^b^	11^b^^,^ ^c^	14^b^^,^ ^c^	24

All loci were considered in analysis.

* Overlapping RDP4 events with CF or SNP analyses were not recorded in CFu or SNPu columns, only the RDP4u column.

a See Supplemental Data Table 10.

b See Supplemental Data Table 11.

c See Supplemental Data Presentation Figure 10.

d See Figure 2.

e Sum of “u” columns only.

The majority of recombination events were detected in the *rbsK* locus in both lineages; to a lesser extent recombination events were also detected in *PK, CHP*, and *lepB* for A-like *Synechococcus* and in the 16S rRNA/ITS region and *pcrA* gene for B'-like *Synechococcus* (Supplemental Data Tables 10–13). Phylogenies for *rbsK*, constructed using RDP4 with sequence data from either side of the predicted breakpoints, were shown to be incongruent providing additional evidence of recombination at this locus in both lineages (Supplemental Data Presentation Figures 8, 9).

In contrast, evidence of recombination between the A-like and B'-like lineages was rare. Blast analyses of 7 MLSA loci on 71 A-like BACs and 4 MLSA loci on 72 B'-like BACs revealed only three examples, all involving *rbsK* loci on A-like (*n* = 1) or B'-like (*n* = 2) BACs that were associated with the genome of the other lineage (Supplemental Data Sheet Section VI). Similarly, of the loci used to study both A-like and B'-like *Synechococcus* lineages, *rbsK* was the only gene for which RDP4 analysis detected evidence of recombination. Only 3 unique recombination events were identified among 123 combined A-like and B'-like *rbsK* sequences in the 511 nt overlapping region (all within B'-like BACs; Supplemental Data Table 14).

The importance of recombination relative to mutation for the evolution of A-like and B'-like *Synechococcus* lineages was estimated independently in two ways: (i) R/θ, which is the ratio at which recombination and mutation occur (McVean et al., 2002, 2004; Didelot and Falush, 2007) using LDHat analysis (reported as a range depending on LDHat method annotated in footnotes of Tables 1, 2 and equivalent to ρ/θ in Clonal Frame analysis), and (ii) r/m, which is a ratio of the probabilities that a given site was altered through recombination or mutation (i.e., how important the effect of recombination was in the diversification of the sample relative to mutation in terms of the number of resulting nucleotide substitutions) as defined by Clonal Frame analysis (Didelot and Falush, 2007; Didelot and Maiden, 2010). For MLSA concatenated datasets, the ratios (R/θ, ρ/θ, and r/m) differed somewhat in the two lineages. Recombination events were generally less frequent than mutation events (ρ/θ ranging from 0.38 to 0.89 for A-like BACs and 0.24 to 0.54 for B'-like BACs, in Clonal frame analysis; R/θ ranging from 0.4 to 1.0 for A-like BACs and 0.44 to 1.06 for B'-like BACs in LDHat analysis; see Tables 1, 2). It was also clear from Clonal frame analysis that recombination had a greater impact than mutation on diversification of the lineage (r/m ratio ranging from 2.60 to 3.40 depending on number of loci for *Synechococcus* A-like BACs, and 2.42 for *Synechococcus* B'-like BACs; Tables 1, 2).

### Ecotype simulation analyses of concatenated A-like *Synechococcus* MLSA datasets

We focus in the main text on the A-like *Synechococcus* lineage, which offered a greater number of genes for MLSA analyses (7 loci, hence termed MLSA7). Also, A-like *Synechococcus* BACs were recovered from both mat samples, thus enabling analysis of habitat association. Comparable results for a 4-locus MLSA analysis (MLSA4) of B'-like lineage variants, which were recovered only from the 60°C sample, are presented in Supplemental Data Sheet Section VII. For clarity, PEs demarcated by Ecotype Simulation are named according to whether they are based on MLSA data (MLSA7 or MLSA4 followed by the PE designation; e.g., MLSA7 PEA1; MLSA4 PEB'4) or single-locus data (specified by the gene analyzed followed by the PE designation; e.g., *rbsK* PEA2).

All individual-locus sequences sampled by BACs and multi-locus concatenations constructed from them (STs) were analyzed using Ecotype Simulation (Figure 2A, Table 4). STs predicted by Ecotype Simulation to be members of the same putative ecotype in the MLSA7 analysis are given the same color, but members of different PEs are colored differently, and colors are maintained throughout all MLSA7 Figures. The MLSA7 concatenated phylogeny and the single-locus phylogeny for *rbsK* sequences sampled by the same BACs are compared in Figures 2A,B respectively, which also show PEs predicted by Ecotype Simulation. Phylogenetic comparisons with other loci (*CHP, lepB, dnaG PK, aroA*, and *hisF*) are discussed in Supplemental Data Sheet Section VIII and are shown in Supplemental Data Presentation Figure 14. Ecotype Simulation predicted 2–7 A-like PEs from individual BAC loci, depending on the locus analyzed. Among individual loci, the greatest number of PEs was predicted from the *rbsK* gene (Figure 2B), which had the greatest average evolutionary divergence among the loci studied (Table 4). Ecotype Simulation predicted 13 A-like MLSA7 PEs from concatenated MLSA sequence data (Figure 2A), though five of these PEs were based on a single ST sequence (i.e., singletons) and one was based on two occurrences of the same ST (i.e., a doubleton). MLSA7 PE clades contained from 1 to 28 BACs. Three of the 13 MLSA7 PE clades for *Synechococcus* A-like BACs contained a dominant variant (i.e., 2–14 clones that were identical at all MLSA7 loci) and singleton variants. In two cases, MLSA7 PEs A4 and A7, subdominant variants were also observed, along with singletons. Supplemental Data Table 23 shows how STs, dominant variants and subdominant variants were distributed among MLSA7 PEs.

**Table 4:** **Ecotype Simulation and eBURST output for 71 A-like *Synechococcus* BACs that contained all 7 loci assayed for the study**.

**Locus**	**Average evolutionary divergence (BACs)**	**Ecotype Simulation (ES)**				**eBURST^b^**	
		**PEs Demarcated-ES (95% CI)**	**Omega (95% CI)**	**Sigma (95% CI)**	**Sample-specific PEs**	**No. of alleles or sequence types**	**No. of clonal complexes**
*rbsK*	0.03	7 (4–16)	0.05 (0.01–0.14)	3.5 (0.33–42)	Yes	24	^d^
*PK*	0.002	2 (2–60)	0.052 (0.04–66)	13 (1.2–12.2)	No	11	^d^
*hisF*^a^	< 0.0001	2 (2–71)	3113 (<2e^−7^–>100)	48,000 (0.7->100)	No	2	^d^
*lepB*	0.005	2 (2–6)	0.09 (0.003–0.6)	26.9 (1.7–99.8)	No	6	^d^
*CHP*	0.01	2 (2–5)	0.03 (0.001–0.23)	564 (5.4–>100)	No	4	^d^
*aroA*	0.001	3 (2–71)	1.86 (0.03–99.6)	22.7 (0.07–100)	No	7	^d^
*dnaG*	0.002	4 (3–22)	0.3 (0.07–2.5)	180 (3.8–>100)	No	7	^d^
Concatenation^b^	0.01	13 (9–44)	0.05 (0.03–0.12)	1.11 (0.07–100)	^c^	50	3

a Molecular resolution of hisF was not significant enough for Ecotype Simulation to effectively predict ecotypes.

b Calculated from concatenated sequence datasets only.

c Not enough 65° sequences to determine sample specificity of demarcated PEs.

d Clonal complexes only apply to concatenated sequence datasets.

Only one case of complete correspondence between *rbsK* PEs and MLSA7 PEs was observed (i.e., MLSA7 PE A9 = *rbsK* PEA 4) (green colored STs in Figure 2). Otherwise, the *rbsK* and MLSA7 phylogenies were mainly incongruent, providing further evidence that recombination complicates single-locus analyses of PEs (compare Figures 2A,B). For instance, some members of MLSA7 PE A4 were classified into three *rbsK* PEs (A1, A2, and A7; red colored sequences in Figure 2). Ecotype Simulation analysis of concatenated MLSA7 loci mainly split individual PEs demarcated by *rbsK* analysis into multiple MLSA7 PEs, as shown by dashed and solid black arrows in Figure 2 (e.g., *rbsK* PE2 was split into MLSA PEs 3 (pink), 4 (red), and 5 (blue).

### Evidence of habitat association

In order to obtain a greater number of A-like BACs from samples collected at both temperatures sufficient to observe habitat associations, we performed two separate MLSA analyses of 5 of the 7 loci (*rbsK, lepB, aroA, CHP*, and *PK*; MLSA5, see Supplemental Data Sheet Section VIII). One included 49 concatenated sequences with nearly equal representation of sequences from 60 and 65°C (Supplemental Data Presentation Figure 15). The other included the full dataset of 145 concatenated sequences that could be compared at these 5 loci (Supplemental Data Presentation Figure 16). Fisher's exact tests of contingency on both sets of BACs from 60 and 65°C yielded significant evidence of heterogeneity in habitat associations (i.e., collection temperatures; *p*-value = 0.001). Additionally, several MLSA5 PEs were comprised of multiple BACs obtained only from one of the two temperatures.

In order to investigate vertical distributions of alleles we pyrosequenced the *rbsK* locus in DNAs extracted from 80 μm-thick cryotome sections from different depths in the top green layer of the 63–65°C mat, where A-like PEs predominate. Pyrosequencing analysis of the *rbsK* locus resulted in a phylogeny (Supplemental Data Sheet Section IX; Supplemental Data Presentation Figure 19) that closely resembled the *rbsK* phylogeny based on sequences sampled by BAC clones (compare Figure 2B and Supplemental Data Presentation Figure 19). In cases where pyrosequences matched only STs of the same PE or subclade, we could test the hypothesis that these PEs represent ecologically distinct populations. As shown in Figure 3A, the subclade representing MLSAs PE A4 and A5 (red and blue STs in Figure 2A), which were grouped together into *rbsK* PE A2 (Figure 2B), were shown to exhibit different vertical distributions (Fisher's exact test, ANOVA and *G*-tests *p* < 0.001). *rbsK* sequences representative of MLSA PE A4 declined with depth, whereas those representative of MLSA PE A5 increased with depth. This provides an example of how a single-locus analysis can lump populations that are ecologically distinct, giving the impression of a clade containing ecologically heterogeneous members. Additionally, the availability of genome sequences of isolates representative of MLSA7 PEs A5 and A9 (Olsen et al., 2015; and see Supplemental Data Sheet Section X) allowed us to associate *rbsK* and *psaA* sequences corresponding to the same PEs. In these cases we could demonstrate correspondence between *rbsK* distributions and the *psaA* distributions observed in single-locus analysis of the same samples by Becraft et al. (2015) (see also Figures 3B,C, where MLSA7 PE 5 = *psaA* PE A6 and MLSA7 PE 9 = *psaA* PE A14). Both of these PEs have been shown to be most prevalent toward the bottom portion of the upper green layer of the mat at this temperature (Becraft et al., 2015), and representative isolates have been shown to possess genes that enable harvesting of lower amounts of light, characteristic of *in situ* light conditions (Becraft et al., 2015; Nowack et al., 2015; Olsen et al., 2015; also see Supplemental Data Sheet Section X).

### eBURST analyses

The eBURST algorithm assembles STs into clonal complexes (Maiden et al., 1998; Feil et al., 1999, 2000a, 2004) that are typically composed of a single predominant genotype (consensus group or here, dominant variant), plus variants that are identical to the predominant genotype at all but one locus (single-locus variants; Feil and Spratt, 2001; Feil et al., 2004). Single-locus variants can differ from the dominant variant by any number of SNPs as long as those SNPs occur in only one locus (Feil et al., 1999, 2000b, 2003). Only 3 clonal complexes were observed with eBURST analyses of the MLSA7 dataset (Figure 4, Table 5), and the correspondence between eBURST's clonal complexes and Ecotype Simulation's PEs was imprecise (see Figure 4 and Supplemental Data Sheet Section VIII for details of these analyses and results). In the eBURST analyses of the MLSA5 datasets, Fisher's exact tests did not indicate differences among clonal complexes in their habitat associations (Supplemental Data Presentation Figure 17, *p*-value 0.943; Supplemental Data Presentation Figure 18, *p*-value 0.734).

Table 5**eBURST analysis of clonal complexes for the 7-locus MLSA of A-like *Synechococcus* BACs**.

**Table:** 

**Clonal Complex A7-I**																	
**Locus**	**Consensus DV-ST1 (14)**	**ST2**	**ST3**	**ST4**	**ST6**	**ST8**	**ST11**	**ST12**	**ST14**	**ST15**	**ST33**	**ST36**	**ST39**	**ST41**	**ST43**	**ST48**	**ST49**
*rbsK*	1	1	1	1	3^1^	1	1	1	1	1	1	7^2^	8^2^	10^5^	12^1^	17^2^	1
*PK*	1	1	1	1	1	2^1^	1	1	8^1^	10^11^	1	1	1	1	1	1	1
*hisF*	1	1	1	1	1	1	1	1	1	1	1	1	1	1	1	1	1
*lepB*	1	1	2^1^	3^13^	1	1	1	1	1	1	1	1	1	1	1	1	1
*CHP*	1	1	1	1	1	1	2^20^	1	1	1	1	1	1	1	1	1	1
*aroA*	1	1	1	1	1	1	1	1	1	1	7^2^	1	1	1	1	1	1
*dnaG*	1	2^1^	1	1	1	1	1	5^19^	1	1	1	1	1	1	1	1	3^2^

**Table:** 

**Clonal Complex A7-II**								
**Locus**	**Consensus DV-ST5 (2)**	**ST11**	**ST18**	**ST19**	**ST21**	**ST31**	**ST45**	**ST46**
*rbsK*	2	1^20^	2	2	2	18^2^	14^8^	15^13^
*PK*	1	1	1	1	8^1^	1	1	1
*hisF*	1	1	1	1	1	1	1	1
*lepB*	1	1	2^1^	1	1	1	1	1
*CHP*	2	2	2	3^1^	2	2	2	2
*aroA*	1	1	1	1	1	1	1	1
*dnaG*	1	1	1	1	1	1	1	1

**Table:** 

**Clonal Complex A7-III**					
**Locus**	**Consensus DV-ST6 (2)**	**ST1**	**ST7**	**ST22**	**ST23**
*rbsK*	3	1^1^	3	3	3
*PK*	1	1	1	1	1
*hisF*	1	1	1	1	1
*lepB*	1	1	1	1	1
*CHP*	1	1	2^20^	1	1
*aroA*	1	1	1	1	5^2^
*dnaG*	1	1	1	4^1^	1

The number of BACs within an ST is in parentheses when greater than 1. Superscripts next to the allele number denote number of nucleotide differences compared to the consensus sequence. Corresponding to Figure 4.

DV, dominant variant.

### Evidence of historic and recent recombination events

**Figure 5:**
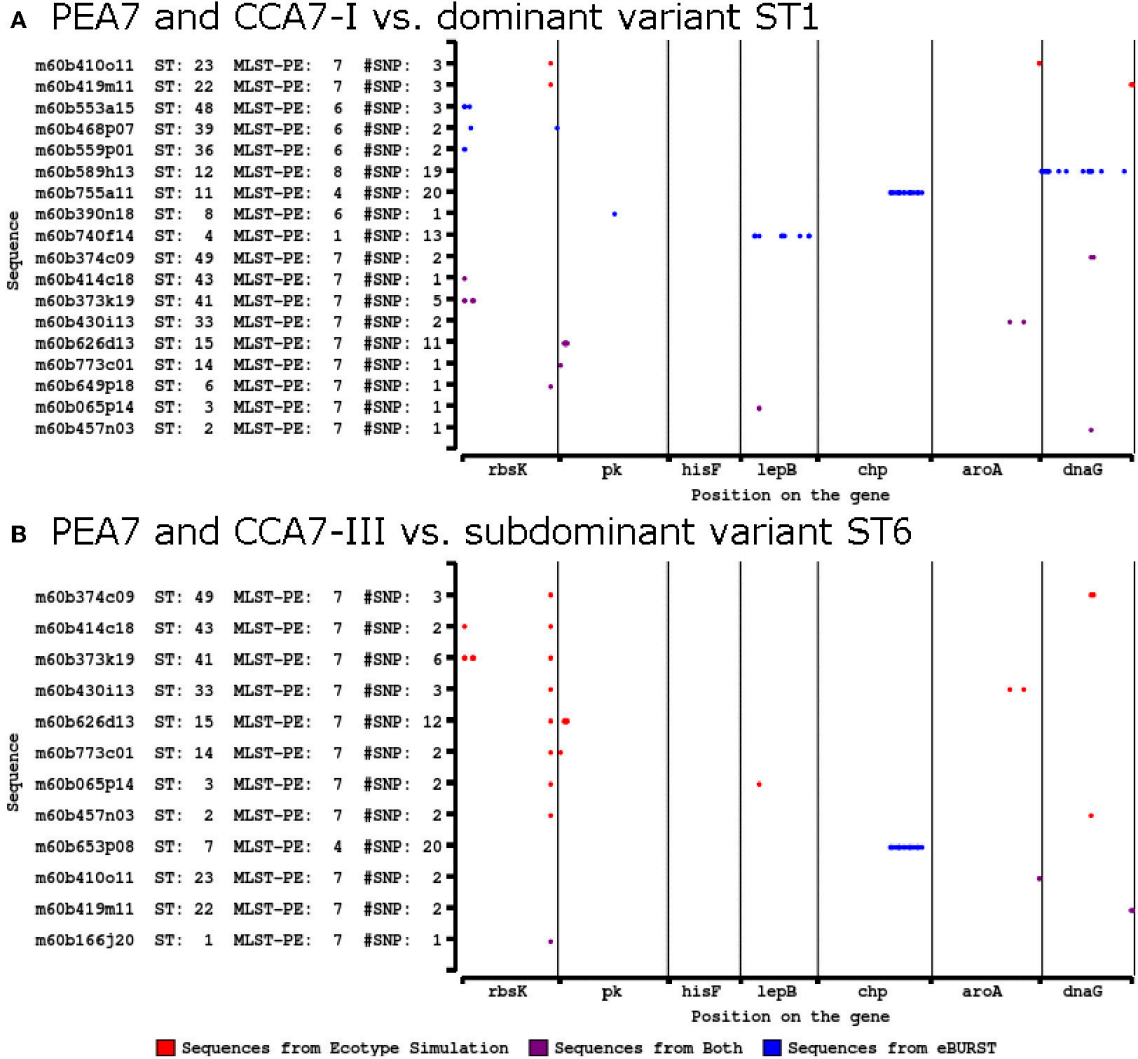
**Single nucleotide polymorphism patterning of A-like *Synechococcus* BACs grouped around the same dominant variant (also consensus sequence) by Ecotype Simulation and eBURST**. Variants detected only by eBURST (blue), only by Ecotype Simulation (red) or by both (purple) are compared to the shared dominant variant (consensus sequence). **(A)** Dominant variant ST1 in PEA7 and clonal complex A7-I and **(B)** subdominant variant ST6 in PE7 and clonal complex A7-III. STs correspond with STs in Figure 2A.

The presence of historic recombination events was further investigated after it was initially found through SNP analysis that identical SNP differences were observed in two different MLSA7 PEs. Figure 5 shows SNP differences between variants that were grouped into MLSA7 PEs and clonal complexes sharing the same dominant variant. Most differences resulted either from the exclusion of STs containing SNPs in multiple loci from clonal complexes by eBURST, or from the exclusion of STs containing a large number of SNPs from phylogenetic clusters demarcated as PEs by Ecotype Simulation. Of particular interest, however, was the observation of identical SNP differences in the *CHP* locus in two different MLSA7 PE A4 STs relative to two different dominant variants of MLSA7 PE A7 (ST7 vs. ST6 and ST11 vs. ST1). This prompted us to consider the possibility that these SNP patterns were clade-specific. We investigated recombination events occurring on internal nodes (suggestive of historic recombination) using Clonal Frame. Clonal Frame predicted several internal nodes in both the *Synechococcus* A-like and B'-like lineages where there was a high probability of recombination having occurred in one or more loci (closed colored stars in Figure 2A and Supplemental Data Presentation Figure 10). This revealed a historical record of the impact recombination has had on the diversification of these organisms. Detailed manual inspection of SNP patterns in all clades allowed us to suggest additional putative recombination events in the phylogeny of these *Synechococcus* lineages (open colored stars in Figure 2A and Supplemental Data Presentation Figure 10; also see Supplemental Data Sheet Section XI and Supplemental Data Presentation Figure 21). For instance, all variants grouped into the clade containing MLSA7 PEs A2-A4 appear to have vertically inherited a recombinant *CHP* gene after an early recombination event (closed green star) and variants within MLSA7 PEs A2, A3, and a portion of PEA4, appear to have vertically inherited an *rbsK* sequence from a subsequent recombination event (closed blue star). A third putative recombination event involving the *rbsK* locus (open blue star) may distinguish MLSA7 PEs A2 and A3 from MLSA7 PE A4.

These SNP analyses also revealed several examples of singleton MLSA7 PEs associated with recombination patterns involving a large number of SNPs [MLSA7 PEs A1 (ST4), A2 (ST27), A8 (ST12), and A11 (ST25); see Figure 2A]. These singleton-PE variants formed long-branches and were closely related to sister PEs comprised of multiple variants, but different in many SNPs from the dominant variant of that PE. For example, in Figures 2A, 5A, singleton-PE A8 (ST12) was closely related to PE A7, but differed by 19 SNPs in the *dnaG* locus, suggesting that a recent recombination event might have caused enough sequence difference to prevent its accurate demarcation by Ecotype Simulation. There was only one case in which an identical SNP pattern shared by two STs (ST25 in singleton MLSA7 PE A11 and ST 34 in MLSA7 PE A9; *lepB*) was not clade-specific (Figure 2 and Supplemental Data Presentation Figure 21 panel I).

Similar analysis was performed on the *Synechococcus* B'-like BACs with the interesting result that 2–3 recombination events were associated with some divergences, even though only 4 loci were included in the B'-like MLSA4 phylogeny (Supplemental Data Sheet Section VIII and Supplemental Data Presentation Figures S10, S12, S13).

## Discussion

### Recombination within and between lineages of *Synechococcus*

There is abundant evidence that genomic rearrangements and recombination have influenced the evolution of the *Synechococcus* populations inhabiting the Mushroom Spring microbial mat. As in previous investigations of *Sulfolobus islandicus* (Cadillo-Quiroz et al., 2012), *Wolbachia* (Ellegaard et al., 2013), and *Vibrio cyclitrophicus* (Shapiro et al., 2012) mentioned above, recombination between the divergent A and B' lineages of *Synechococcus* appears to be less frequent than recombination within each lineage. Specifically, only approximately 2% of BACs exhibited evidence of recombination between A- and B'-like *Synechococcus* BACs (see Results, Supplemental Data Sheet Section VI and Supplemental Data Table 14). This corroborates our previous metagenomic analyses, in which we found a low percentage of metagenomic clones with end sequences stemming from both the A-like and B'-like lineages (Klatt et al., 2011) (Supplemental Data Sheet Section VI). In contrast, within-lineage recombination was greater, with 5.5% of A-like and 33.3% of B'-like *Synechococcus* BACs exhibiting evidence of recombination for approximately equal numbers of loci analyzed (MLSA5, 145 sequences and MLSA4, 72 sequences for *Synechococcus* A- and B'-like BACs respectively; Table 3).

Recombination events have occurred at various times in the history of the A-like and B'-like clades: some recombinant segments are fixed in large subclades (i.e., evidenced by clustering of SNPs in MLSA PEs 2, 3, and 4 in Supplemental Data Presentation Figures 21A,D,G or MLSA PEs 10, 11, 12, and 13 in Supplemental Data Presentation Figures 21C,F,I; for analogous B'-like fixed recombinant segments see Supplemental Data Presentation Figure 13), indicating a recombination event early in the clade's history followed by vertical inheritance of the recombined gene, while others are specific only to singleton (one doubleton) variants demarcated as PEs based on recent recombination in single loci (i.e., MLSA PEs 1, 2, 8, 10, 11, and 13 in Figure 2A). These results suggest that recent recombination events might cause overestimation of PEs by Ecotype Simulation. That is, a single recombination event, if involving a long enough fragment, can cause Ecotype Simulation to split a PE. In the case of eBURST, a single recombination event of any size cannot split a consensus group; however, two or more recombinants of any size will separate a recipient organism into a different consensus group.

The rates of recombination within the A and B' lineages were estimated to be less than or about the same as that of mutation (Tables 1, 2). However, we determined that recombination had a somewhat greater impact than mutation on sequence change within these populations (Tables 1, 2; Clonal Frame, r/m range 1.58–4.92). The impact of recombination (r/m) was estimated at 2.6 and 2.4, for the *Synechococcus* A-like and B'-like lineages, respectively. These r/m ratios are similar to values reported for other cyanobacteria (Vos and Didelot, 2009) and are in the same range as those reported for *S. islandicus, Wolbachia*, and *Halorubrum* (Papke et al., 2004; Whitaker et al., 2005; Ellegaard et al., 2013). The recent study on *Synechococcus* by Rosen et al. (2015), did not provide r/m, but their estimate of the ratio of recombination to mutation rates (ρ/θ) was close to 1, which fell within the R/θ range provided by our LDHat analysis (Tables 1, 2). Why do such different interpretations arise from such similar results? Although, Rosen et al. interpreted from a very similar recombination/mutation ratio that recombination is “ubiquitous,” we note that this ratio is not extraordinarily high when compared against the many groups of bacteria whose recombination rates were quantified by Vos and Didelot (2009). Furthermore, Rosen et al. have not considered the theoretical work of Haldane (1932), which shows that such low rates of recombination cannot prevent ecological diversification.

Given the rates of mutation estimated for various bacteria (Drake, 2009; Vos and Didelot, 2009), we may conclude that a typical gene in the *Synechococcus* populations is experiencing recombination at an extremely low *absolute* rate, so that recombination between nascent species is unlikely to hinder their divergence (Haldane, 1932; Vos, 2011; Wiedenbeck and Cohan, 2011). Low-frequency recombination may allow shuffling of niche-neutral alleles, which do not determine ecological niche, but low-frequency recombination of *niche-determining* alleles from other populations can be countered by natural selection (Haldane, 1932). Maladaptive niche-determining alleles from other populations will be kept at negligible levels, maintaining the integrity of populations' adaptations (Wiedenbeck and Cohan, 2011). Rates of recombination observed in *Synechococcus* should not lead us to dismiss *a priori* the possibility of ecological diversification within either clade.

We therefore disagree with Rosen et al.'s conclusion (Rosen et al., 2015) that frequent recombination has caused each of the A and B' clades of *Synechococcus* to be a “quasisexual population,” where close relatives are prevented from diversifying into long-standing, ecologically homogeneous populations, or ecotypes. While Rosen et al. (2015) have shown a shuffling of alleles (really gene segments) within the A and B' clades, they have not shown that the recombination has randomized associations between niche-determining alleles, which are responsible for ecotype divergence. Moreover, we do not see how they could rule out ecological diversification without studying ecology. As we have shown in many previous papers, as well as in the present study, there is ample evidence for the coexistence of long-standing, ecologically divergent populations within both the A and B' clades, as we discuss below. Rosen et al. mentioned some of these studies, but did not use an ecological model in their work, as we have done here.

As with most (if not all) bacteria, recombination has been sufficient in *Synechococcus* to create new ecological diversity. Within the A lineage, for example, low-light PEs differ from high-light PEs in having acquired a gene cluster likely coding for an additional photoreceptor (Olsen et al., 2015). It does not take a recombination rate much higher than that of mutation to transfer a gene that is adaptive in a new niche. Only one adaptive transfer of a gene (or one mutation event); see Bantinaki et al. (2007) is required to found a new ecotype and thereby bring about ecological diversification. On the other hand, a much higher rate of recombination is required to prevent adaptive divergence between nascent populations; this requires recurrent inter-population transfer of genes poorly adapted to their new niche (Wiedenbeck and Cohan, 2011).

### Ecological diversification within the A-like and B'-like *Synechococcus* lineages

The Ecotype Simulation analysis of concatenated MLSA sequences hypothesized 13 and 29 PEs within the A-like and B'-like clades, respectively. This was greater than the number of PEs predicted from single-locus analyses of the most divergent locus, *rbsK*. This is likely because of greater resolution provided by MLSA7 (as well as MLSA4; see Supplemental Data Presentation Figure 10), as evidenced by the fact that MLSA7 split numerous PEs that were lumped by *rbsK* analysis. We were able to demonstrate the ecological distinctness of some of the A-like putative ecotypes, first with respect to the two habitat types investigated in this study (60 and 65°C). That is, some hypothesized A-like ecotypes were sampled entirely from different habitats, while others were quantitatively different in their habitat associations. Also, pyrosequencing of PCR-amplified *rbsK* genes typical of different MLSA7 PEs demonstrated fine-scale distribution differences of MLSA7 PEs along vertical gradients. Additionally, the availability of genomes of isolates representative of MLSA7 PEs permitted us to demonstrate correspondence of *rbsK* distributions with distributions observed earlier in *psaA* analyses (Becraft et al., 2015). This led to demonstration of adaptation consistent with distribution patterns (Nowack et al., 2015) and genomic evidence of mechanisms likely to underlie these adaptations (Olsen et al., 2015). For instance, MLSA PEs 5 and 9 correspond to *psaA* PEs A6 and A14, whose distribution deep in the upper green layer of the mat (Becraft et al., 2015) is consistent with the presence of auxiliary photosystem genes that are likely to be associated with the ability to acclimate to low irradiance (Nowack et al., 2015; Olsen et al., 2015); and see Supplemental Data Sheet Section X). Becraft et al. (2011, 2015) also describe the existence and ecological distinction of B'-like *Synechococcus* ecotypes, as well as the ecological interchangeability of the members of each ecotype.

We conclude that both the A-like and B'-like *Synechococcus* lineages have clearly diversified into many younger species populations, whose niches correspond in part to differences along temperature and vertical gradients (Becraft et al., 2015). Our approach did not permit us to evaluate the role of positive selection within our dataset as our genes were under neutral/purifying selection. Evaluation of full genomes of strains of closely related species will allow us to further examine the roles that recombination, mutation, selection and neutral processes have played in the diversification of *Synechococcus*.

### Whither the biological species concept?

That recombination has occurred more frequently within closely related clades than between them has previously been taken as support for the Biological Species Concept in bacteria and archaea (Cadillo-Quiroz et al., 2012; Shapiro et al., 2012; Ellegaard et al., 2013). However, the quintessential idea of the Biological Species Concept is that sexual isolation is required for irreversible divergence and ecological distinctness in the origin of species (Cohan, 2013). Support for the Biological Species Concept requires more than a correlation between sexual isolation and divergence among clades; one must demonstrate that reduced recombination was necessary for their divergence.

We argue that such evidence is lacking in the above-cited studies on *Vibrio, Wolbachia, and Sulfolobus*. As seen in the present work, *Synechococcus* lineages have been noted to have recombination levels similar to those reported for *Halorubrum* and *Sulfolobus*. Like these organisms, major *Synechococcus* clades that are highly divergent (3.6% diverged at the 16S rRNA locus and 18% diverged in average nucleotide identity (ANI), Bhaya et al., 2007) are ecologically distinct. However, our high-resolution analysis of closest relatives within each clade has shown ecological speciation among organisms that have identical or nearly identical 16S rRNA sequences and have diverged only on the order of 1% ANI (Olsen et al., 2015; also see Supplemental Data Table 25) and show minimal sexual isolation. Similarly, in work on another system we have found ecotype differentiation among *Bacillus* clades that show very little sexual isolation (Majewski and Cohan, 1999; Connor et al., 2010). We hypothesize that analysis of fine-scale ecological divergence within *Halorubrum* or *Sulfolobus* may yield similar results (Papke et al., 2007; Cadillo-Quiroz et al., 2012). Recombination does not appear to have impeded ecological diversification among close relatives that are not sexually isolated. Thus, sexual isolation, in this context, is unnecessary for the origin of species (Wiedenbeck and Cohan, 2011).

Instead, sexual isolation is more likely to be the result of divergence among closely related groups than the cause. Not only do ecologically distinct populations tend to diverge over time into separate sequence clusters (Cohan and Perry, 2007), but ecological divergence may also cause occupancy of different microhabitats, reducing opportunities for genetic exchange (Shapiro et al., 2012); in addition, functional divergence may result in reduced fitness of recombinants (Retchless and Lawrence, 2010). For other mechanisms of sexual isolation, also see Wiedenbeck and Cohan (2011). Regarding evidence for the Biological Species Concept provided by previous bacterial and archaeal studies, we note that two of these studies have provided no direct evidence that the groups of sequences studied have distinct ecological properties (Cadillo-Quiroz et al., 2012; Ellegaard et al., 2013), and rely primarily on the coexistence of sequence clusters as evidence that they utilize different resources. A third study by Shapiro et al looking at genetic exchange between two recently diverged populations of *Vibrio* (Shapiro et al., 2012) reported similar patterns of sub-clustering within the *Vibrio* populations (their Figure S2), and we predict that these sub-clusters would show ecological diversification if the authors were to test this. We predict each subgroup would represent an ecotype, subject to genomic sweeps. The fourth study (Rosen et al., 2015) claimed that there cannot be discrete ecotypes within the *Synechococcus* A and B' clades, owing to recombination, yet the existence of such discrete ecotypes has been amply demonstrated here and in many earlier papers (Becraft et al., 2015; Nowack et al., 2015; Olsen et al., 2015).

Most importantly, an argument for the Biological Species Concept should provide evidence that ecological diversification was prevented among groups hypothesized to recombine more frequently. This would require a search for ecological diversification among the close relatives recombining at the highest rates. While previous studies have not attempted to search for ecological diversity among closest relatives (within the major clades); this has been a principal aim of our study.

## Conclusion

In conclusion, it appears that we have found some of the most newly divergent ecotypes of hot spring *Synechococcus*. They do recombine, and recombination has not prevented their divergence, as had been suggested by Doolittle and Zhaxybayeva (2009). Because a reduction in recombination does not appear necessary for ecological divergence, and because ecological patterning of bacterial and archaeal diversity is common, the Biological Species Concept does not appear appropriate for bacterial and archaeal speciation. While recombination has not hindered speciation, ongoing genomic and metagenomic analyses have begun to reveal evidence suggesting that (i) horizontal genetic transfer and recombination have likely led to ecological diversification by introducing niche-transcending adaptations to new lineages (Gogarten et al., 2002; Lawrence, 2002; Doolittle and Zhaxybayeva, 2009; Wiedenbeck and Cohan, 2011; Olsen et al., 2015), and (ii) mutation and selection have also played a role in the evolutionary ecology of *Synechococcus* ecological species (Olsen et al., 2015). Sub-clustering within major clades predicted to be ecologically distinct has been observed across a variety of taxa and habits (Smith et al., 2006; Zinser et al., 2006; Hunt et al., 2008; Connor et al., 2010; Denef et al., 2010; Mazard et al., 2012), suggesting that our observations may have general importance to the issue of species and speciation in bacteria and archaea. It is important to keep in mind that the molecular dimension of microbial species may be smaller than we have previously thought, and that it is important to understand theories underlying species and speciation.

## Materials and methods

Samples were collected on 2 October 2003 from the effluent channel of Mushroom Spring (44.5386°N, 110.7979°W), an alkaline siliceous hot spring in the Lower Geyser Basin of Yellowstone National Park, WY, at two temperatures, 60 and 65°C. DNA extraction and construction of bacterial artificial chromosome (BAC) clones and oligonucleotide probe screening to identify BACs containing a *Synechococcus* A/B lineage-specific 16S rRNA gene are described in the Supplemental Data Sheet Section I. DNA from parallel samples was used for analyses of the 16S rRNA and ITS region on the BACs (Ward et al., 2006) and PCR-amplified single protein-encoding loci (*rbsK, aroA*, and *apcAB*) (Melendrez et al., 2011), as well as for construction of small-insert (2–12 kb) metagenomic libraries (Bhaya et al., 2007; Klatt et al., 2011).

### Locus selection

From one hundred genes that were upstream and downstream of the two unlinked 16S rRNA genes in the *Synechococcus* strain A and B' genomes, loci were selected based on the following characteristics: (i) presence in both genomes, (ii) range of distances from the 16S rRNA locus, (iii) high degree of nucleotide divergence between *Synechococcus* strain A and B' homologs, (iv) high average nucleotide divergence and variance of metagenomic homologs from *Synechococcus* strain A and B' homologs, (v) not under positive evolutionary selection (dN/dS values < 1) and (vi) functionally useful for gene expression studies. In addition, loci adjacent to transposons or mobile elements were avoided due to the greater possibility of co-migration of adjacent loci. Loci used in this study are presented in Supplemental Data Tables 1, 2.

### PCR amplification and sequencing of BAC MLSA loci

Primers for the separate amplification of *Synechococcus* A-like and B'-like BAC target genes (Supplemental Data Tables 1, 2) were designed and tested as described for amplification of *rbsK, aroA*, and *apcAB* gene segments by Melendrez et al. (2011), which also provides details of PCR conditions and gel-based size verification and purification of amplicons. Cycling conditions for the *dnaG, pcrA, ispE, sufB*, and *argD* genes were the same used for *aroA* and *rbsK* genes, and cycling conditions for the protein kinase (*PK*), *lepB*, and *accC* genes were the same as for the *apcAB* gene, except for the use of 40 cycles instead of 30 cycles. Cycling conditions for *hisF* and the conserved hypothetical protein (*CHP* [locus tag CYA_2291]) gene were: an initial denaturing step at 94°C (2 min) followed by 20 cycles of 94°C (1 min), 60°C (1 min), and 72°C (1 min); then 20 cycles of 94°C (1 min), 55°C (1 min), and 72°C (1 min) with a final extension at 72°C for 10 min and storage at 4°C. BAC clones exhibiting no PCR product for any gene were amplified two more times (directly from the BAC clone sample) to increase confidence that the gene was absent.

### Sequencing

Purified PCR products were sequenced using the forward and reverse primers for each gene described in Supplemental Data Tables 1, 2 and the BigDye v.3.1 cycle sequencing kit (Applied Biosystems) at the University of Nevada-Reno Sequence Center (Reno, NV). The sequences have been submitted to GenBank, via the BankIt submission tool; Accession numbers: *pcrA*; KT425377-KT425447, *rbsK;* HQ662694-HQ662843, HQ187926-187996, KT425519-KT425589, and KT426096-KT426240, 16S rRNA; KT425590-KT425660, *PK* (protein kinase); KT425661-KT425805, *CHP* (conserved hypothetical protein); KT425806-KT425950, *lepB*; KT425951-KT426095, *aroA*; HQ187854-HQ187925, KT425447-KT425518, and KT426241-426385, *dnaG*; KT426386-KT426455, *hisF*; KT426456-KT426525.

### Sequence alignment and phylogenetic analysis

Bidirectional sequence data for each locus were analyzed using Sequencher v.4.8. Sequence data were then analyzed with NCBI-BLAST nr/nt (Altschul et al., 1990) to determine whether the top match was with genomic homologs of *Synechococcus* strains JA-3-3Ab [accession: CP000239.1], JA-2-3B'a(2-13) [accession: CP000240.1], or neither. Sequences used in MLSA were concatenated using a custom perl script (available from J.M. Wood) and uploaded in MEGA4. Alignments were made using the ClustalW algorithm in the MEGA4 software (Tamura et al., 2007) or the MUSCLE (Edgar, 2004) algorithm implemented in Seaview (Gouy et al., 2010). The *Synechococcus* strain A and B' genomes were included as references (Bhaya et al., 2007). MLSA including newly obtained isolates are described in Supplemental Data Sheet Section X. Alignments were analyzed using jModeltest2 (Darriba et al., 2012) to determine the best model fit for maximum likelihood tree construction. Maximum likelihood trees were constructed using PhyML (Guindon et al., 2010) as implemented in Seaview (Gouy et al., 2010) with aLRT support and edited using MEGA4 (Tamura et al., 2007) or Figtree (Rambaut, 2009). Estimates of average evolutionary divergence for single and multi-locus phylogenies were computed over all sequence pairs using the Maximum Composite Likelihood method in MEGA4 as previously described (Tamura et al., 2004, 2007; Melendrez et al., 2011).

### Ecotype simulation and demarcation

Concatenated and single-locus sequence alignments were analyzed using Ecotype Simulation to predict the number of PEs (n), rates of periodic selection (sigma), ecotype formation (omega), and 95% confidence intervals (CI) for all parameters at the best precision match between observed and simulated data for that sequence dataset (between 1.25x and 2x). Ecotypes were manually demarcated conservatively as previously described (Cohan and Perry, 2007; Koeppel et al., 2008; Becraft et al., 2011; Melendrez et al., 2011) (http://fcohan.web.wesleyan.edu/ecosim/). Statistical tests were conducted using the R statistical package (http://cran.r-project.org/). Groups (rows) were defined as PEs that contained >5 sequences. The variables being tested were temperature (60 and 65°C) and vertical (80 μm intervals) distributions. Significance level was set at 0.05.

### Multi-locus and eBURST analyses

Alignments for each gene used in MLSA were organized into groups that were 100% identical at the nucleotide level using Sequencher v 4.8. Allele types were assigned for each unique sequence and were used to generate allelic profiles; BACs with identical allelic profiles at all loci were assigned as unique sequence types (STs), as described in Supplemental Data Tables 8, 9, 19, 20. Allelic profiles and their unique ST designation were uploaded into eBURST (Feil et al., 2004; Spratt et al., 2004) and population snapshots were generated to view A-like and B'-like *Synechococcus* diversity. Clonal complexes (CC) were defined as a consensus group of BACs and at least 3 single-locus variants, as suggested (Feil et al., 2004). Population snapshots to visualize CCs with less stringent criteria were generated by defining CCs as a consensus group with at least 2 single-locus variants and/or allowing for double-locus variants to be included in the CC and are defined as sub-clonal complexes (see Supplemental Data Sheet Section XII).

### Single nucleotide polymorphism analysis

SNPs were analyzed by comparing sequences of PE clade variants and single-locus variants with that of the dominant variant of the same PE clade or consensus group of the same CC (often the same as the dominant variant) using the Perl program, Pigeon (https://github.com/sandain/pigeon). Pigeon reads in the FASTA file and compares each nucleotide in the dominant variant to the corresponding nucleotide in each PE variant (or single-locus variant), locating and reporting the position of SNPs.

### Detection of recombination signals

Five methods were utilized to detect recombination in single-gene and concatenated datasets; (i) outlier detection on phylogeny followed by BLAST (http://blast.ncbi.nlm.nih.gov/Blast.cgi), (ii) RDP version 4 (RDP4) (Martin et al., 2015), (iii) Clonal Frame version 1.1 (Didelot and Falush, 2007), (iv) SNP analysis (PIGEON; https://github.com/sandain/pigeon), and (v) LDHat v2.2 (McVean et al., 2002).

(i) BLAST. Phylogenies were manually inspected for long branch lengths. Those STs/clones which fell on long branch lengths were analyzed using BLAST (bl2seq program) against isolate genomes (CP000239.1 and CP000240.1) to ensure that all genes within the concatenation had the highest match to the appropriate isolate genome as determined by 16S rRNA analysis (also see Supplemental Data Sheet Section VI).
(ii) RDP identifies recombinants in either multi-locus or single-gene sequence datasets. All loci for A-like and B'-like *Synechococcus* populations were tested for putative recombination events using Recombination Detection Program, version 4 (RDP4) (Martin et al., 2015). The loci were tested individually and as concatenated sequences. In the RDP suite of programs a number of different methods are implemented. The methods used for recombination detection in this study included the RDP method (Martin et al., 2005), GENECONV (Sawyer, 1989), Maximum Chi Square (Smith, 1992; Posada and Crandall, 2001), Chimera (Posada and Crandall, 2001), Sister Scanning (Siscan) (Gibbs et al., 2000), 3SEQ (Boni et al., 2007), and Likelihood Assisted Recombination Detection (LARD), which constitute some of the most powerful methods currently available (RDP4 manual, Martin et al., 2005). The general settings within RDP4 were as follows: the highest acceptable *p*-value was set to 0.05 with Bonferroni corrections. For the individual methods default parameters were used for all methods with the following exceptions:
In RDP, the window size was set to 30 as recommended (RDP4 Instruction Manual, available at http://web.cbio.uct.ac.za/~darren/RDP4Manual.pdf). In MaxChi and Chimera the 'variable window size' was used. In Siscan the window size was set to 200 bp with a step size of 20.
Recombination signals were considered present if they could be detected by at least 3 methods within the RDP4 package at significant *p*-values as suggested (RDP4 manual).
(iii) Clonal Frame identifies evidence of recombination in multi-locus sequence datasets (Didelot and Falush, 2007). Extended multi-FASTA formatted alignments (XMFA) were generated using the MAUVE program (Darling et al., 2004, 2007) and given file extensions of.dat. DAT files were loaded into Clonal Frame and analyzed using default settings with 1,000,000 iterations after burn in, 250,000 burnin, sampling every 100 iterations and verbose mode (-v,-x,-y,-z options). OUT files with phylogenies and statistics (mutation events, recombination events, rho/theta and r/m) were visualized using the cfgui.bat program included in the Clonal Frame installation. Recombination events identified along branches are noted with closed stars for MLSA datasets in Figure 2A and Supplemental Data Presentation Figure 10.
(iv) SNP analysis. The patterning of PE variant and single-locus variant SNPs against the dominant variant sequence was also considered in interpreting possible evidence of recombination events (method described above). Recombination events inferred by SNP analysis are noted with open stars on branches for MLSA datasets in Figure 2A and Supplemental Data Presentation Figure 10.
(v) LDHat estimates a per-locus population recombination parameter (R), the average per-site population mutation rate (Watterson's θ), average pairwise distance (avePWD) and variance within the average pairwise difference (varPWD). These were determined using three methods within the LDHat v2.2a package (McVean et al., 2002); the composite-likelihood method, Rmin calculation and Wakeley's moment method (all implemented using convert, pairwise and associated lookup tables generated either from precompiled tables included with package distribution or generated directly from the sequence data). All analyses were run using default parameters. The recombination rate calculated by LDHat assumes a constant recombination rate over the region and a gene conversion model with either a finite sites model (composite likelihood) or infinite sites model (Rmin calculation). We note that the linkage of our loci caused by sampling a portion of the genome as well as sequencing only a portion of each gene may possibly cause underestimation or large range of recombination ratios if recombined segments tend to span several genes or if the recombined segment was larger than the amplified gene segment thereby capturing one ‘breakpoint’ but not the other (see Supplemental Data Sheet Section VI).

### Pyrosequencing

PCR primers and protocols described by Melendrez et al. (2011) or in Supplemental Data Table 1 were used to amplify *rbsK* sequences from Mushroom Spring samples collected at ~63–65°C and sectioned along vertical gradients (~80 μm intervals using a cryotome). Samples were collected on 13 and 14 September 2008 and DNA was extracted as described in Becraft et al. (2011). Barcoding and Ti454-sequencing were completed at the J. Craig Venter Institute according to the GS FLX Titanium Series Rapid Library Preparation Method. DNA was sheared using the Covaris S2 System, and qPCR was used to accurately estimate the number of molecules needed for emPCR. BAC clone MLSA sequences were trimmed at the 5′ and 3′ ends to match the average sequence length, which collapsed some SNP variation contained in the *rbsK* single-locus analysis and caused a decrease in molecular resolution. Homopolymer inserts were removed by aligning sequences to the *rbsK* reading frame of genomes from the A or B' *Synechococcus* isolates. High- frequency sequences (sequences totaling ≥50 identical copies in all combined samples) were then identified and PEs were demarcated from high-frequency sequences using Ecotype Simulation. Population percentages based on high-frequency sequences that matched STs of a single MLSA PE were calculated as described in Becraft et al. (2015). Sequence data for *rbsK* was deposited into the MG-RAST version 4 database (https://metagenomics.anl.gov/) under accession numbers 4642340.3-4642367.3.

## Funding

This research was supported by the National Science Foundation Frontiers in Integrative Biology Research Program (EF-0328698), the National Aeronautics and Space Administration Exobiology Program (NAG5-8807 and NX09AM87G), and the U.S. Department of Energy (DOE), Office of Biological and Environmental Research (BER), as part of BER's Genomic Science Program (GSP). This contribution originates from the GSP Foundational Scientific Focus Area (FSFA) at the Pacific Northwest National Laboratory (PNNL), contract no. 112443. It was also supported by Montana Agricultural Experiment Station project 911352.

### Conflict of interest statement

The authors declare that the research was conducted in the absence of any commercial or financial relationships that could be construed as a potential conflict of interest.

## Supplemental data

The Supplemental Data for this article can be found online at: http://journal.frontiersin.org/article/10.3389/fmicb.2015.01540
