# Supplementary material for: Recombination Does Not Hinder Formation or Detection of Ecological Species of Synechococcus Inhabiting a Hot Spring Cyanobacterial Mat
Source: Front Microbiol. 2016 Jan 14;6:1540. doi: 10.3389/fmicb.2015.01540 (PMC4712262; doi:10.3389/fmicb.2015.01540)
Supplement: Supplementary file 1 [file Table1.docx]

Supplementary Material Tables

Recombination does not hinder formation or detection of ecological species of *Synechococcus* inhabiting a hot spring cyanobacterial mat

Melanie C. Melendrez^1*^, Eric Becraft^1^, Jason M. Wood^1^, Millie T. Olsen^1^, Donald A. Bryant^2^, John F. Heidelberg^3^, Doug B. Rusch^4^, Frederick M. Cohan^5^ and David M. Ward^1^

^1^ Department of Land Resources and Environmental Science, Montana State University, Bozeman, MT, USA

^2^ Department of Biochemistry and Molecular Biology, Pennsylvania State University, University Park, PA, USA

^3^ Department of Biological Sciences, College of Letters, Arts and Sciences, University of Southern California, Los Angeles, CA, USA

^4^ Informatics Group, J. Craig Venter Institute, Rockville, MD, USA

^5^ Department of Biology, Wesleyan University, Middletown, CT, USA

*** Correspondence:** Walter Reed Army Institute of Research, Viral Diseases Branch, 503 Robert Grant Ave. Silver Spring, MD 20910 USA. [mmelendrez@gmail.com](mailto:mmelendrez@gmail.com)

## Supplementary Table 1. Characteristics of loci used in analysis of A-like *Synechococcus* mat populations.

| **Locus** | **Genomic annotation^a^** | **Primer**  **Sequence**  **(5´-3´)** | **Gene Length** | **Amplified fragment length** | **Distance from the 16S rRNA locus (kb)** | ***Synechococcus* strain A/B´ homolog divergence (%)** | **No. of Metagenomic Sequences** | **Average % Divergence of Metagenomic and A-like homologs** | **Variance of Metagenomic and A-like homologs** | **dN/dS ratio^f^** |
| --- | --- | --- | --- | --- | --- | --- | --- | --- | --- | --- |
| *apcAB** | Allo-phycocyanin alpha/beta subunits and IS region^b^ | Ref. ([1](#_ENREF_1)) | 972 | 500 | 82 | 8.5 | 7 | 0.1 | 0.01 | 0.01 |
| *rbsK* | Ribokinase | Ref. ([1](#_ENREF_1)) | 930 | 582 | 50 | 18.2 | 3 | ^d^ | ^d^ | 0.08 ^e^ |
| *PK* | Hypothetical protein kinase | *PK*F:atccatgccctttcgcttggaaac  *PK*R:tgacttcagcggtagaaatcggct | 2166 | 654 | 39 | ^c^ | 5 | 1.7 | 1.5 | 0.96 |
| *hisF* | Imidazole-glycerol phosphate synthase, cyclase subunit | hisFF:ccactcacgaagagcgggaaatct  hisFR:ggcaattgtcaactggccgtagtg | 762 | 429 | 23 | 11.5 | 7 | 0.4 | 0.01 | 0.71 |
| *lepB* | Signal peptidase I | lepBF:gagaacttgctgacagtggtgctg  lepBR:gatactctggcggggtgaaaaagt | 687 | 462 | 28 | 14.5 | 2 | ^d^ | ^d^ | 0.66 ^e^ |
| *CHP* | Conserved hypothetical protein | *CHP*F:tcgaggacatgaagggccaaatct  *CHP*R:aaatgggtcgtcaaagccgtttcc | 2106 | 678 | 15 | ^c^ | 9 | 1.8 | 1.9 | 0.09 |
| *aroA* | 3-phospho-shikimate 1-carboxyvinyl-transferase | Ref. ([1](#_ENREF_1)) | 1335 | 657 | 38 | 15.1 | 6 | 0.3 | 0.05 | 0.82 |
| *dnaG* | DNA primase | dnaGF:cacaacgaccacaagcccagcttt  dnaGR:ttccgtttccggggagttgaggta | 1896 | 546 | 24 | 12 | 10 | 0.3 | 0.02 | 0.88 |

* Considered but not used in this study.

^a^ Links to genomic annotations for *Synechococcus* strain A: <http://cmr.jcvi.org/cgi-bin/CMR/GenomePage.cgi?org=gyma>

^b^ The *apcAB* amplified product contained 380 bp from *apcA,* 60 bp of *apcB* and 60 bp of the internal sequence in between.

^c^ The conserved hypothetical protein (*CHP*) and protein kinase (*PK*) genes do not contain a homolog in the *Synechococcus* strain B´ genome.

^d^ Number of metagenomic sequence < 5 so this statistic was not calculated.

^e^ Calculated based on < 5 sequences

^f^ Based on >5 metagenomic reads.

## Supplementary Table 2. Characteristics of loci used in MSLA analysis of B´-like *Synechococcus* populations.

| **Locus** | **Genomic annotation^a^** | **Primer**  **Sequence**  **(5´-3´)** | **Gene**  **Length** | **Amplified fragment**  **length** | **Distance from the 16S rRNA locus**  **(kb)** | ***Synechococcus***  **strain A/B´**  **homolog**  **divergence (%)** | **No. of Metagenomic Sequences** | **Average % Divergence of Metagenomic and B´-like homologs** | **Variance**  **of Metagenomic and B´-like homologs** | **dN/dS**  **ratio** |
| --- | --- | --- | --- | --- | --- | --- | --- | --- | --- | --- |
| *aroA* | 3-phospho-shikimate 1-carboxyvinyl-transferase | Ref. ([1](#_ENREF_1)) | 1305 | 550 | 52 | 15.1 | 10 | 3.5 | 2.0 | 1.1 |
| *rbsK* | Ribokinase | Ref. ([1](#_ENREF_1)) | 918 | 535 | 24 | 18.2 | 14 | 3.1 | 4.0 | 0.7 |
| *pcrA* | ATP-dependent DNA helicase | pcrAF:attgcctacctggttcgccactat  pcrAR:tgacgggttggcctgagaacttta | 2364 | 630 | 8 | 13.7 | 19 | 2.5 | 2.1 | 1.0 |
| *ispE** | 4-diphospho-cytidyl-2C-methyl-D-erythritol kinase | ispEF:gcgatggtgttgcagagcattcat  ispER:aaccacctgttccaagtcattgcg | 936 | 499 | 14 | 19 | 7 | 2.6 | 2.1 | 0.2 |
| *accC** | acetyl-CoA carboxylase, biotin carboxylase | accCF:ggttcttggcggagaatgccaaat  accCR:aatcagatccagccctgtcaccat | 1356 | 580 | 15 | 8.1 | 11 | 2.5 | 2.8 | 0.04 |
| *sufB** | FeS assembly protein | sufBF:agtcgattggcaaaggcttgaacg  sufBR:tcttggagtttgctccctgacaca | 1437 | 679 | 27 | 5.0 | 15 | 2.9 | 2.7 | 0.1 |
| *argD** | Acetyl-ornithine transaminase | argDF:aagccaatgagggagccattaagc  argDR:gctgggcattccaaaccaaactct | 1266 | 551 | 38 | 13.8 | 13 | 0.99 | 1.3 | 0.1 |
| *apcAB** | Allo-phycocyanin alpha/beta subunits and IS region^b^ | Ref. ([1](#_ENREF_1)) | 972 | 498 | 61 | 8.5 | 11 | 1.8 | 2.2 | 0.1 |

* Considered but not used in this study.

^a^ Links to genomic annotations for *Synechococcus* strain B´: <http://cmr.jcvi.org/cgi-bin/CMR/GenomePage.cgi?org=gymb>.

^b^ The *apcAB* amplified product contained 380 bp from *apcA,* 55 bp of *apcB* and 63 bp of the internal sequence in between.

## Supplementary Table 3. Percent recruitment by clusters defined in 2-12 kbp metagenome assembly analyses and BAC libraries (60^o^C and 65^o^C combined).

| Cluster^1^ | Phylogeny | % sequence in short-insert metagenome sequences recruited by cluster | % BAC end sequences recruited by cluster |
| --- | --- | --- | --- |
| 1 | *Synechococcus* spp. | 24.7% | 8.5% |
| 2 | *Roseiflexus* spp. | 7.9% | 15.8% |
| 3 | *Chloroflexus* spp. | 0.61% | 1.1% |
| 4 | *Candidatus* Cab. thermophilum-like organisms | 13.2% | 10.4% |
| 5 | *Chlorobiales*-like organisms | 5.0% | 14.8% |
| 6 | *Anaerolineae*-like organisms | 7.5% | 11.9% |
| 7 | Unknown 1 | 4.8% | 11.2% |
| 8 | Unknown 2 | 1.9% | 3.2% |
| Null^2^ |  | 31.9% | 23.4% |

^1^ These clusters were determined by repeated K-means analysis of the oligonucleotide usage frequency of ≥ 20 kb Celera assemblies (see ([2](#_ENREF_2))).

^2^ “Null” indicates that we have low confidence in the sequences having any relationship with clusters or genomes used in these analyses.

## Supplementary Table 4. Comparison of BAC and small-insert metagenomic library compositions and synteny with reference genomes.

| **Reference**  **Genome** | **% nt identity range^a^** | **% of sequences recruited** | | | | **% Syntenous/Non-syntenous Clones^b^**  **of No. total sequences in analysis** | | | | **Estimated BAC library fold coverage by^c^** | |
| --- | --- | --- | --- | --- | --- | --- | --- | --- | --- | --- | --- |
|  |  | Small-Insert | | BAC | | Small-Insert | | BAC | | End sequences | Cloned DNA |
|  |  | MS60^a^ | MS65^a^ | 60^o^C | 65^o^C | MS60^a^ | MS65^a^ | 60^o^C | 65^o^C |  |  |
| *Synechococcus* strain A | 83-100 | 7.8 | 25.7 | 4.0 | 8.0 | 62/12  (1257) | 69/20 (10172) | 30.8/29.0  (689) | 1.4/74.6  (1308) | 0.175X | 139X |
| *Synechococcus* strain B´ | 90-100 | 22.3 | 1.14 | 5.18 | 1.04 | 72/19  (3414) | 7/6  (928) | 21.5/56.7  (808) | n.d. | 0.076X | 118X |
| *Roseiflexus* sp. RS-1 | 80-100 | 15.99 | 9.58 | 7.92 | 5.94 | 58/28  (2793) | 41/26  (5616) | 0/46.1  (1397) | 2.1/61.5  (3056) | 0.135X | 101X |
| *Chloroflexus* sp. 396-1 | 65-100 | 0.76 | 15.90 | 1.77 | 15.97 | 28/2  (201) | 77/9  (5071) | 0.6/8.7  (366) | 0/27.8  (1016) | --- | --- |
| *Candidatus* Chloroacidobacterium thermophilum | 70-100 | 9.65 | 2.13 | 6.31 | 1.64 | 70/15  (1663) | 37/14  (1582) | 45.0/36.6  (2813) | 2.3/54.1  (968) | 0.272X | 367X |
| *Chloroherpeton thalassium* | 50-75 | 6.46 | 2.11 | 10.11 | 2.25 | 14/25  (748) | 6/22  (655) | 0.5/26.4  (1770) | 0.7/26.7  (292) | --- | --- |
| *Thermomicrobium roseum* | 75-100 | 0.21 | 0.66 | 2.0 | 3.0 | 15/10  (192) | 18/7  (1141) | 1.3/7.4  (297) | 1.7/24.5  (595) | 0.017X | 8.9X |

^a^ Small-insert data obtained from Klatt et al. ([2](#_ENREF_2)) was from libraries constructed from the same mat samples used to construct the BAC clone libraries but with different lysing and library construction protocols.

^b^ Number of syntenous or non-syntenous sequences divided by the total number of sequences, including disjointly recruited sequences.

^c^ Coverage by end sequences was estimated by multiplying the number of BAC end sequences above the sequence cutoff for each recruitment bin in each library by the approximate length of Sanger sequences (800 bp), then dividing by the number of bp in the recruiting genome. These numbers were added to determine total coverage by end sequence data. Coverage by cloned BAC DNA was then estimated by dividing the estimate of coverage/recruitment bin/library by the fraction of the library actually end-sequenced (4% for 60^o^C and 14.2% for 65^o^C), then multiplying by the ratio of approximate insert length (100,000 bp/BAC clone) divided by the approximate total end sequence per BAC clone (1600 bp). Coverages for 60^o^C and 65^o^C were then summed.

## Supplementary Table 5. Distributions of mate-pair types of jointly-recruited end sequences of randomly selected and cyanobacterial (cyano) BAC clones.

| **Type^1^** | **60^o^C BAC Library** | | | | **65^o^C BAC Library** | |
| --- | --- | --- | --- | --- | --- | --- |
|  | ***Synechococcus* sp. strain A** | | ***Synechococcus* sp.**  **strain B´** | | ***Synechococcus* sp. strain A** | |
|  | **Random (%)** | **Cyano (%)** | **Random (%)** | **Cyano (%)** | **Random (%)** | **Cyano (%)** |
| G-G | 50 | 39 | 27 | 11 | 2 | 32 |
| G-L | 7 | 18 | 12 | 16 | 7 | 16 |
| G-S | 34 | 1 | 18 | 0 | 73 | 3 |
| Anti-G | 0.5 | 0 | 2 | 0.6 | 1 | 1 |
| Anti-L | 0 | 27 | 11 | 24 | 5 | 23 |
| Anti-S | 1 | 0 | 1 | 0 | 1 | 2 |
| Nor-G | 0 | 0 | 2 | 1 | 0.4 | 0 |
| Nor-L | 3 | 15 | 14 | 35 | 6 | 15 |
| Nor-S | 0.5 | 0 | 0.6 | 0 | 0.8 | 0 |
| Out-G | 0 | 0 | 0.3 | 0.6 | 0.2 | 0.6 |
| Out-L | 0.5 | 0 | 10.4 | 11 | 4 | 7 |
| Out-S | 0 | 0 | 1 | 0 | 0.4 | 0 |

^1^ G: Good, L: Long, S: Short, Anti: Antinormal, Nor: Normal, Out: Outie. For further explanation of mate pair types, see ref ([3](#_ENREF_3)).

## Supplementary Table 6. Locus distribution of *Synechococcus* A-like BACs based on PCR amplification.

| **60^o^C and 65^o^C *Synechococcus* A-like BACs with Sequence Data for MLSA Loci** | | | | | | | | | | | | | | | | | | | | | | |
| --- | --- | --- | --- | --- | --- | --- | --- | --- | --- | --- | --- | --- | --- | --- | --- | --- | --- | --- | --- | --- | --- | --- |
| *apcAB* | | *rbsK* | | *PK* | | *lepB* | | *hisF* | | *CHP* | | *aroA* | | 16S rRNA | | d*naG* | | # of Protein- Encoding Loci | No. 60^o^C BACs | | No. 65^o^C BACs | Total |
| + | | + | | + | | + | | + | | + | | + | | + | | + | | 8 | 57 | | 7 | 64 |
|  | | + | | + | | + | | + | | + | | + | | + | | + | | 7 | 90 | | 7 | 97 |
| + | | + | | + | | + | | + | | + | | + | | + | |  | | 7 | 83 | | 8 | 91 |
|  | |  | | + | | + | | + | | + | | + | | + | | + | | 6 | 93 | | 7 | 100 |
|  | | + | | + | | + | | + | | + | | + | | + | |  | | 6 | 126 | | 10 | 136 |
|  | |  | |  | | + | | + | | + | | + | | + | | + | | 5 | 111 | | 9 | 120 |
|  | |  | | + | | + | | + | | + | | + | | + | |  | | 5 | 130 | | 10 | 140 |
|  | |  | |  | |  | |  | | + | | + | | + | | + | | 3 | 127 | | 28 | 155 |
|  | |  | |  | |  | |  | | + | | + | | + | |  | | 2 | 181 | | 45 | 226 |
|  | |  | |  | |  | |  | |  | | + | | + | |  | | 1 | 193 | | 57 | 250 |
|  | |  | |  | |  | |  | |  | |  | | + | | + | | 1 | 145 | | 28 | 173 |
| Number of BACs with Expected Loci | | | | | | | | | | | | | | | | | | | 159 | | 17 | 176 |
| ***Synechococcus* A-like BACs with missing MLSA Loci^a^** | | | | | | | | | | | | | | | | | | | | | | |
| + | + | | + | | + | |  | | + | | + | | + | | + | | 7 | | | 6 | 14 | 20 |
| + | + | | + | | + | | + | |  | | + | | + | | + | | 7 | | | 1 | 0 | 1 |
| + | + | | + | | + | |  | | + | | + | | + | |  | | 6 | | | 4 | 4 | 8 |
|  | + | | + | | + | |  | | + | | + | | + | | + | | 6 | | | 5 | 0 | 5 |
|  | + | | + | | + | | + | | + | |  | | + | | + | | 6 | | | 2 | 0 | 2 |
| + | + | |  | | + | |  | | + | | + | | + | | + | | 6 | | | 1 | 0 | 1 |
| + | + | | + | | + | | + | |  | | + | | + | |  | | 6 | | | 1 | 1 | 2 |
|  |  | | + | | + | |  | |  | | + | | + | |  | | 6 | | | 1 | 0 | 1 |
| + |  | | + | | + | | + | | + | | + | | + | |  | | 6 | | | 1 | 0 | 1 |
| + |  | |  | | + | |  | | + | | + | | + | | + | | 6 | | | 0 | 1 | 1 |
|  | + | |  | | + | |  | | + | | + | | + | |  | | 5 | | | 3 | 0 | 3 |
| + | + | |  | | + | |  | | + | | + | | + | |  | | 5 | | | 1 | 0 | 1 |
| + |  | | + | | + | | + | |  | | + | | + | |  | | 5 | | | 0 | 1 | 1 |
| + | + | | + | | + | |  | |  | | + | | + | |  | | 5 | | | 0 | 2 | 2 |
| + |  | | + | | + | |  | | + | | + | | + | |  | | 5 | | | 0 | 3 | 3 |
|  | + | | + | | + | |  | | + | | + | | + | |  | | 5 | | | 0 | 2 | 2 |
|  |  | | + | | + | |  | | + | | + | | + | | + | | 5 | | | 1 | 0 | 1 |
|  |  | | + | | + | |  | | + | | + | | + | |  | | 4 | | | 1 | 1 | 2 |
|  |  | |  | | + | |  | | + | | + | | + | | + | | 4 | | | 6 | 3 | 9 |
| + | + | |  | | + | |  | |  | | + | | + | |  | | 4 | | | 0 | 1 | 1 |
|  |  | |  | | + | |  | | + | | + | | + | |  | | 3 | | | 0 | 2 | 2 |
|  |  | |  | | + | | + | |  | |  | | + | | + | | 3 | | | 1 | 0 | 1 |
| + |  | |  | | + | |  | |  | | + | | + | |  | | 3 | | | 0 | 5 | 5 |
|  |  | | + | |  | |  | |  | | + | | + | |  | | 2 | | | 1 | 0 | 1 |
|  | + | |  | | + | |  | |  | |  | | + | |  | | 2 | | | 3 | 0 | 3 |
|  | + | |  | |  | |  | | + | |  | | + | |  | | 2 | | | 2 | 0 | 2 |
|  | + | |  | |  | |  | |  | | + | | + | | + | | 3 | | | 1 | 0 | 1 |
| + | + | |  | |  | |  | |  | |  | | + | |  | | 2 | | | 1 | 0 | 1 |
|  | + | |  | |  | |  | |  | |  | | + | | + | | 2 | | | 7 | 0 | 7 |
| + |  | |  | |  | |  | |  | |  | | + | |  | | 1 | | | 0 | 1 | 1 |
| Number of BACs with missing ‘internal’ loci | | | | | | | | | | | | | | | | | | | | 50 | 41 | 91 |

^a^ BACs that did not yield an amplicon after 3 PCR attempts. These BACs were not used in subsequent analyses of the *Synechococcus* A-like population 7-locus MLSA study but some 65^o^C BACs were included in the 5-locus MLSA study.

## Supplementary Table 7. Locus distribution on *Synechococcus* B´-like BACs based on PCR amplification.

| **60^o^C *Synechococcus* B´-like BACs with Sequence Data for MLSA Loci** | | | | | | | | | | | | | | | | | | | | |
| --- | --- | --- | --- | --- | --- | --- | --- | --- | --- | --- | --- | --- | --- | --- | --- | --- | --- | --- | --- | --- |
| *aroA* | | *rbsK* | | *pcrA* | | 16S rRNA | | *ispE* | | *accC* | | *sufB* | | *argD* | | *apcAB* | | # of Protein-Encoding Loci | | No. 60^o^C BACs |
| + | | + | | + | | + | | + | | + | | + | | + | |  | | 7 | | 2 |
|  | | + | | + | | + | | + | | + | | + | | + | | + | | 7 | | 1 |
|  | | + | | + | | + | | + | | + | | + | | + | |  | | 6 | | 8 |
|  | |  | | + | | + | | + | | + | | + | | + | | + | | 6 | | 4 |
| + | | + | | + | | + | | + | | + | |  | |  | |  | | 5 | | 18 |
|  | |  | |  | | + | | + | | + | | + | | + | | + | | 5 | | 35 |
| + | | + | | + | | + | | + | |  | |  | |  | |  | | 4 | | 28 |
|  | | + | | + | | + | | + | | + | |  | |  | |  | | 4 | | 47 |
|  | |  | |  | | + | | + | | + | | + | | + | |  | | 4 | | 48 |
| + | | + | | + | | + | |  | |  | |  | |  | |  | | 3 | | 92 |
|  | | + | | + | | + | | + | |  | |  | |  | |  | | 3 | | 65 |
|  | | + | | + | | + | |  | |  | |  | |  | |  | | 2 | | 188 |
|  | |  | |  | | + | | + | | + | |  | |  | |  | | 2 | | 83 |
|  | |  | | + | | + | |  | |  | |  | |  | |  | | 1 | | 197 |
|  | |  | |  | | + | | + | |  | |  | |  | |  | | 1 | | 102 |
| Number of BACs with Expected Loci | | | | | | | | | | | | | | | | | | | | 222 |
| ***Synechococcus* B´-like BACs with missing MLSA Loci^a^** | | | | | | | | | | | | | | | | | | | | |
| + | + | |  | | + | | + | |  | | + | |  | |  | | 4 | | 2 | |
| + |  | | + | | + | | + | | + | |  | |  | |  | | 4 | | 1 | |
|  | + | |  | | + | |  | |  | |  | |  | |  | | 2 | | 8 | |
| + |  | | + | | + | |  | |  | |  | |  | |  | | 2 | | 4 | |
| Number of BACs with Missing ‘internal’ Loci | | | | | | | | | | | | | | | | | | | 15 | |

^a^ BACs that did not yield an amplicon after 3 PCR attempts. These BACs were not used in subsequent analyses.

## Supplementary Table 8. Allelic profiles generated from analysis of single nucleotide polymorphisms in the A-like *Synechococcus* BACs for protein-encoding sequence datasets of 7 loci.

| **BAC Clone^a^** | **ST^b^** | ***rbsK*** | ***pk*** | ***hisF*** | ***lepB*** | ***CHP*** | ***aroA*** | ***dnaG*** |
| --- | --- | --- | --- | --- | --- | --- | --- | --- |
| M60B166J20 | 1 | 1 | 1 | 1 | 1 | 1 | 1 | 1 |
| M60B405K13 | 1 | 1 | 1 | 1 | 1 | 1 | 1 | 1 |
| M60B471D04 | 1 | 1 | 1 | 1 | 1 | 1 | 1 | 1 |
| M60B501M02 | 1 | 1 | 1 | 1 | 1 | 1 | 1 | 1 |
| M60B502F22 | 1 | 1 | 1 | 1 | 1 | 1 | 1 | 1 |
| M60B514A21 | 1 | 1 | 1 | 1 | 1 | 1 | 1 | 1 |
| M60B516C08 | 1 | 1 | 1 | 1 | 1 | 1 | 1 | 1 |
| M60B651G15 | 1 | 1 | 1 | 1 | 1 | 1 | 1 | 1 |
| M60B658F14 | 1 | 1 | 1 | 1 | 1 | 1 | 1 | 1 |
| M60B663O15 | 1 | 1 | 1 | 1 | 1 | 1 | 1 | 1 |
| M60B689K07 | 1 | 1 | 1 | 1 | 1 | 1 | 1 | 1 |
| M60B701L08 | 1 | 1 | 1 | 1 | 1 | 1 | 1 | 1 |
| M60B729E1 | 1 | 1 | 1 | 1 | 1 | 1 | 1 | 1 |
| M60B788A07 | 1 | 1 | 1 | 1 | 1 | 1 | 1 | 1 |
| M60B457N03 | 2 | 1 | 1 | 1 | 1 | 1 | 1 | 2 |
| M60B574L23 | 2 | 1 | 1 | 1 | 1 | 1 | 1 | 2 |
| M60B065P14 | 3 | 1 | 1 | 1 | 2 | 1 | 1 | 1 |
| M60B533H13 | 3 | 1 | 1 | 1 | 2 | 1 | 1 | 1 |
| M60B740F14 | 4 | 1 | 1 | 1 | 3 | 1 | 1 | 1 |
| M60B761P11 | 4 | 1 | 1 | 1 | 3 | 1 | 1 | 1 |
| M60B399F15 | 5 | 2 | 1 | 1 | 1 | 2 | 1 | 1 |
| M60B461H19 | 5 | 2 | 1 | 1 | 1 | 2 | 1 | 1 |
| M60B649P18 | 6 | 3 | 1 | 1 | 1 | 1 | 1 | 1 |
| M60B690I15 | 6 | 3 | 1 | 1 | 1 | 1 | 1 | 1 |
| M60B653P08 | 7 | 3 | 1 | 1 | 1 | 2 | 1 | 1 |
| M60B733O24 | 7 | 3 | 1 | 1 | 1 | 2 | 1 | 1 |
| M60B390N18 | 8 | 1 | 2 | 1 | 1 | 1 | 1 | 1 |
| M60B413O21 | 8 | 1 | 2 | 1 | 1 | 1 | 1 | 1 |
| M65B009D20 | 9 | 5 | 5 | 1 | 1 | 1 | 1 | 1 |
| M60B527I20 | 9 | 5 | 5 | 1 | 1 | 1 | 1 | 1 |
| *Synechococcus* strain A | 10 | 24 | 11 | 2 | 2 | 1 | 4 | 7 |
| M60B755A11 | 11 | 1 | 1 | 1 | 1 | 2 | 1 | 1 |
| M60B589H13 | 12 | 1 | 1 | 1 | 1 | 1 | 1 | 5 |
| M65B080L10 | 13 | 22 | 1 | 1 | 1 | 1 | 2 | 1 |
| M60B773C01 | 14 | 1 | 8 | 1 | 1 | 1 | 1 | 1 |
| M60B626D13 | 15 | 1 | 10 | 1 | 1 | 1 | 1 | 1 |
| M60B247G10 | 16 | 2 | 1 | 1 | 2 | 2 | 1 | 2 |
| M65B090N22 | 17 | 23 | 1 | 1 | 1 | 1 | 2 | 1 |
| M60B725H17 | 18 | 2 | 1 | 1 | 2 | 2 | 1 | 1 |

Supplementary Table 8 Continued…

| M60B195D22 | 19 | 2 | 1 | 1 | 1 | 3 | 1 | 1 |
| --- | --- | --- | --- | --- | --- | --- | --- | --- |
| M60B760D02 | 20 | 21 | 5 | 1 | 1 | 1 | 1 | 1 |
| M60B443F23 | 21 | 2 | 8 | 1 | 1 | 2 | 1 | 1 |
| M60B419M11 | 22 | 3 | 1 | 1 | 1 | 1 | 1 | 4 |
| M60B410O11 | 23 | 3 | 1 | 1 | 1 | 1 | 5 | 1 |
| M60B715I02 | 24 | 20 | 4 | 1 | 2 | 1 | 1 | 1 |
| M60B595E04 | 25 | 19 | 3 | 1 | 3 | 1 | 1 | 6 |
| M65B115I21 | 26 | 4 | 1 | 1 | 1 | 1 | 4 | 1 |
| M60B177G14 | 27 | 4 | 1 | 1 | 5 | 2 | 2 | 1 |
| M60B499M16 | 28 | 4 | 6 | 1 | 1 | 4 | 2 | 1 |
| M60B503C09 | 29 | 4 | 6 | 1 | 1 | 2 | 1 | 1 |
| M60B478F03 | 30 | 4 | 1 | 1 | 1 | 2 | 2 | 1 |
| M60B563B21 | 31 | 18 | 1 | 1 | 1 | 2 | 1 | 1 |
| M60B183F17 | 32 | 5 | 7 | 1 | 1 | 1 | 1 | 1 |
| M60B430I13 | 33 | 1 | 1 | 1 | 1 | 1 | 7 | 1 |
| M60B609H12 | 34 | 6 | 4 | 1 | 3 | 1 | 1 | 1 |
| M60B504G12 | 35 | 6 | 4 | 1 | 1 | 1 | 1 | 1 |
| M60B559P01 | 36 | 7 | 1 | 1 | 1 | 1 | 1 | 1 |
| M60B560H17 | 37 | 7 | 1 | 1 | 2 | 1 | 1 | 2 |
| M60B491M01 | 38 | 8 | 2 | 1 | 1 | 1 | 1 | 1 |
| M60B468P07 | 39 | 8 | 1 | 1 | 1 | 1 | 1 | 1 |
| M60B349M23 | 40 | 9 | 3 | 1 | 4 | 1 | 3 | 2 |
| M60B373K19 | 41 | 10 | 1 | 1 | 1 | 1 | 1 | 1 |
| M60B379G20 | 42 | 11 | 3 | 1 | 1 | 1 | 1 | 1 |
| M60B414C18 | 43 | 12 | 1 | 1 | 1 | 1 | 1 | 1 |
| M60B436G19 | 44 | 13 | 7 | 1 | 4 | 2 | 6 | 2 |
| M60B463L24 | 45 | 14 | 1 | 1 | 1 | 2 | 1 | 1 |
| M60B541E20 | 46 | 15 | 1 | 1 | 1 | 2 | 1 | 1 |
| M60B547N16 | 47 | 16 | 9 | 1 | 6 | 1 | 3 | 1 |
| M60B553A15 | 48 | 17 | 1 | 1 | 1 | 1 | 1 | 1 |
| M60B374C09 | 49 | 1 | 1 | 1 | 1 | 1 | 1 | 3 |
| M60B360H08 | 50 | 1 | 2 | 1 | 1 | 1 | 1 | 2 |

^a^ Clone names were compiled from spring (M = Mushroom), temperature (60 or 65), BAC library plate (1-792 for Mushroom Spring 60^o^; 1-168 for Mushroom Spring 65^o^) and specific well of that plate (A-P and 1-24). Example: M65134I01, Mushroom Spring, 65°C library, plate #134 well I-01.

^b^ ST = sequence type.

## Supplementary Table 9. Allelic profiles generated from analysis of single nucleotide polymorphisms in the B´-like *Synechococcus* BACs for protein-encoding datasets of 4 loci.

| **Bac Clone^a^** | **ST^b^** | ***aroA*** | ***pcrA*** | ***rbsK*** | **16S/ITS** |
| --- | --- | --- | --- | --- | --- |
| M60B067L11 | 1 | 1 | 1 | 1 | 1 |
| M60B089D09 | 1 | 1 | 1 | 1 | 1 |
| M60B174D01 | 1 | 1 | 1 | 1 | 1 |
| M60B259J13 | 1 | 1 | 1 | 1 | 1 |
| M60B518L02 | 1 | 1 | 1 | 1 | 1 |
| M60B588P04 | 1 | 1 | 1 | 1 | 1 |
| M60B614H18 | 1 | 1 | 1 | 1 | 1 |
| M60B636L16 | 1 | 1 | 1 | 1 | 1 |
| M60B699K08 | 1 | 1 | 1 | 1 | 1 |
| M60B709P04 | 1 | 1 | 1 | 1 | 1 |
| M60B091H06 | 2 | 2 | 2 | 2 | 2 |
| M60B186K12 | 2 | 2 | 2 | 2 | 2 |
| M60B347P16 | 2 | 2 | 2 | 2 | 2 |
| M60B513G21 | 2 | 2 | 2 | 2 | 2 |
| M60B648C08 | 2 | 2 | 2 | 2 | 2 |
| M60B769K22 | 2 | 2 | 2 | 2 | 2 |
| M60B057N07 | 3 | 1 | 3 | 6 | 1 |
| M60B085O17 | 3 | 1 | 3 | 6 | 1 |
| M60B626O23 | 3 | 1 | 3 | 6 | 1 |
| M60B714K10 | 3 | 1 | 3 | 6 | 1 |
| M60B458H11 | 4 | 2 | 8 | 8 | 1 |
| M60B594L19 | 4 | 2 | 8 | 8 | 1 |
| M60B718A23 | 4 | 2 | 8 | 8 | 1 |
| M60B527C11 | 5 | 4 | 5 | 4 | 4 |
| M60B623B05 | 5 | 4 | 5 | 4 | 4 |
| M60B078P12 | 6 | 3 | 2 | 5 | 3 |
| M60B554K06 | 6 | 3 | 2 | 5 | 3 |
| M60B475H14 | 7 | 1 | 1 | 1 | 5 |
| M60B614C12 | 8 | 1 | 1 | 1 | 13 |
| M60B018J02 | 9 | 1 | 1 | 1 | 17 |
| M60B041O05 | 10 | 1 | 1 | 1 | 20 |
| M60B100J22 | 11 | 1 | 1 | 1 | 26 |
| M60B543D23 | 12 | 1 | 1 | 16 | 1 |
| M60B541D06 | 13 | 1 | 7 | 17 | 1 |
| M60B468M14 | 14 | 1 | 7 | 19 | 1 |
| M60B700J15 | 15 | 1 | 11 | 15 | 1 |
| M60B041P24 | 16 | 2 | 1 | 3 | 21 |
| M60B456A21 | 17 | 2 | 2 | 2 | 11 |
| M60B397N23 | 18 | 2 | 6 | 2 | 6 |
| M60B129N02 | 19 | 2 | 6 | 7 | 29 |
| M60B772E10 | 20 | 2 | 6 | 21 | 1 |
| M60B345J11 | 21 | 2 | 8 | 8 | 5 |

Supplementary Table 9 Continued…

| M60B157O24 | 22 | 2 | 8 | 8 | 18 |
| --- | --- | --- | --- | --- | --- |
| M60B433E13 | 23 | 3 | 1 | 3 | 1 |
| M60B420G12 | 24 | 3 | 2 | 5 | 8 |
| M60B455A17 | 25 | 3 | 2 | 5 | 10 |
| M60B674F15 | 26 | 3 | 2 | 5 | 15 |
| M60B115G12 | 27 | 3 | 2 | 5 | 28 |
| M60B754M13 | 28 | 3 | 2 | 14 | 3 |
| M60B450N20 | 29 | 3 | 13 | 5 | 3 |
| M60B456P18 | 30 | 4 | 5 | 3 | 4 |
| M60B684G15 | 31 | 4 | 5 | 4 | 1 |
| M60B400M20 | 32 | 4 | 5 | 4 | 6 |
| M60B399J16 | 33 | 4 | 5 | 4 | 7 |
| M60B623N20 | 34 | 4 | 5 | 4 | 14 |
| M60B499C24 | 35 | 5 | 6 | 7 | 1 |
| M60B426N01 | 36 | 5 | 6 | 7 | 5 |
| M60B249G11 | 37 | 6 | 9 | 13 | 19 |
| M60B081I21 | 38 | 6 | 9 | 13 | 25 |
| M60B090B21 | 39 | 7 | 4 | 3 | 1 |
| M60B113A15 | 40 | 7 | 4 | 20 | 27 |
| M60B046B18 | 41 | 8 | 8 | 11 | 22 |
| M60B250B15 | 42 | 8 | 15 | 12 | 23 |
| M60B015H24 | 43 | 9 | 8 | 18 | 16 |
| *Synechococcus* sp. B´ | 44 | 10 | 10 | 3 | 1 |
| M60B703M24 | 45 | 11 | 4 | 3 | 1 |
| M60B579B21 | 46 | 12 | 3 | 9 | 12 |
| M60B075B13 | 47 | 13 | 16 | 3 | 24 |
| M60B081O16 | 48 | 14 | 17 | 11 | 1 |
| M60B477G06 | 49 | 15 | 12 | 2 | 2 |
| M60B421K10 | 50 | 16 | 14 | 10 | 9 |
| M60B403F16 | 51 | 17 | 6 | 7 | 1 |

^a^ Clone names were compiled from spring (M = Mushroom), temperature (60), BAC library plate (1-792 for Mushroom Spring 60^o^) and specific well of that plate (A-P and 1-24). Example: M60703M24, Mushroom Spring, 60^o^C library, plate #703 well M-24.

^b^ ST = sequence type.

## Supplementary Table 10. Analysis of recombination signals in A-like *Synechococcus* BAC sequences in three studies (MLSA7, MLSA5-49 sequences and MLSA5-145 sequences^a^).

| **Locus** | **Alignment**  **Analysis^b^** | **Recombinant BAC** | **ST^b,e^** | **CC**  **(PE-DVST)** | **CC Cons. ST** | **No. total SNP differences with cons. ST and/or (dominant variant-PE)** | **Position** | **Parents** | **Evidence^k,^** **^q^** |
| --- | --- | --- | --- | --- | --- | --- | --- | --- | --- |
| *rbsK* | MLSA5(145) | M65B149H05^c,g,l^ | 4 | ^f^(10-4) | ^f^ | ^f(j)^ | 156-358 | M60B207O10/M65B035K07 | RDP4/CF |
| *rbsK* | MLSA5(145) | M65B093I20^d,l^ | 4 | ^f^(10-4) | ^f^ | ^f(j)^ | 156-358 | M60B207O10/M65B035K07 | RDP4/CF |
| *rbsK* | MLSA5(145) | M65B134I01^d,l^ | 4 | ^f^(10-4) | ^f^ | ^f(j)^ | 156-358 | M60B207O10/M65B035K07 | SNP/RDP4/CF |
| *rbsK* | MLSA5(145) | M65B145I07^d,l^ | 4 | ^f^(10-4) | ^f^ | ^f(j)^ | 156-358 | M60B207O10/M65B035K07 | RDP4/CF |
| *rbsK* | MLSA5(145) | M65B155E13^d,l^ | 4 | ^f^(10-4) | ^f^ | ^f(j)^ | 156-358 | M60B207O10/M65B035K07 | RDP4/CF |
| *rbsK* | MLSA5(145) | M60B467J19^d,l^ | 18 | A5-2(10-4) | 18 | ^j^ (15) | 156-358 | M60B207O10/M65B035K07 | RDP4 |
| *rbsK* | MLSA5(145) | M60B504G12^d,l^ | 17 | A5-1(10-4) | 17 | ^j^ (7) | 156-358 | M60B207O10/M65B035K07 | RDP4 |
| *rbsK* | MLSA5(145) | M60B609H12^d,l^ | 46^i^ | A5-1(9-^f^) | 17 | 8(^f^) | 156-358 | M60B207O10/M65B035K07 | RDP4/CF |
| *rbsK* | MLSA5(145) | M60B680J12 ^d,l^ | 18 | A5-2(10-4) | 18 | ^j^(15) | 156-358 | M60B207O10/M65B035K07 | RDP4 |
| *rbsK* | MLSA5(145) | M60B715I02 ^d,l^ | 45^i^ | A5-1(10-4) | 18 | 7(8) | 156-358 | M60B207O10/M65B035K07 | SNP/RDP4 |
| *rbsK* | MLSA5(145) | M60B730H18 ^d,l^ | 17 | A5-1(10-4) | 17 | ^j^(7) | 156-358 | M60B207O10/M65B035K07 | RDP4 |
| *rbsK* | MLSA5(145) | M65B053E10 ^d,l^ | 47^i^ | A5-2(10-4) | 18 | 1(16) | 156-358 | M60B207O10/M65B035K07 | RDP4 |
| *rbsK* | MLSA5(145) | M65B150M20 ^d,l^ | 44^i^ | ^f^(19-^f^) | ^f^ | ^f^ | 156-358 | M60B207O10/M65B035K07 | RDP4 |
| *rbsK* | MLSA5(145) | M60B573N02^c^ | 65^i^ | ^f^(1-^f^) | ^f^ | ^f^ | 48-252 | OSB/M60B379G20 | RDP4 |
| *rbsK* | MLSA5(145) | M60B379G20^c,g^ | 56^i^ | ^f^(7-10) | ^f^ | ^f^(13) | 231-518^h^ | M60B573N02/M60B534F12 | RDP4 |
| *rbsK* | MLSA5(145) | OSA | 55^i^ | ^f^(2-^f^) | ^f^ | ^f(f)^ | 231-518^h^ | M60B573N02/M60B534F12 | RDP4 |
| *rbsK* | MLSA5(145) | M65B038L09^d^ | 53^i^ | ^f^(3-^f^) | ^f^ | ^f(f)^ | 231-518^h^ | M60B573N02/M60B534F12 | RDP4 |
| *rbsK* | MLSA5(145) | M65B080L10^d^ | 54^i^ | ^f^(3-^f^) | ^f^ | ^f(f)^ | 231-518^h^ | M60B573N02/M60B534F12 | RDP4 |
| *rbsK* | MLSA5(145) | M65B141F14^d^ | 52^i^ | A5-7(3-^f^) | 2 | 48^(f)^ | 231-518^h^ | M60B573N02/M60B534F12 | SNP/RDP4 |
| *rbsK* | MLSA5(145) | M60B207O10^d^ | 9 | ^f^(5-9) | ^f^ | ^f(j)^ | 231-518^h^ | M60B573N02/M60B534F12 | RDP4 |
| *rbsK* | MLSA5(145) | M60B393F20^d^ | 10 | A5-7(7-10) | 2 | 54(^j^) | 231-518^h^ | M60B573N02/M60B534F12 | SNP/RDP4 |
| *rbsK* | MLSA5(145) | M60B595E04^d^ | 57^i^ | ^f^(7-10) | ^f^ |  | 231-518^h^ | M60B573N02/M60B534F12 | RDP4/CF |
| *rbsK* | MLSA5(145) | M60B349M23^d^ | 9 | ^f^(5-9) | ^f^ | ^f(j)^ | 231-518^h^ | M60B573N02/M60B534F12 | RDP4 |
| *rbsK* | MLSA5(145) | M60B547N16^d^ | 63^i^ | ^f^(5-9) | ^f^ | ^f(j)^ | 231-518^h^ | M60B573N02/M60B534F12 | RDP4 |
| *rbsK* | MLSA5(145) | M60B691E09^d^ | 51^i^ | ^f^(4-^f^) | ^f^ | ^f(f)^ | 231-518^h^ | M60B573N02/M60B534F12 | RDP4 |
| *rbsK* | MLSA5(145) | M60B071F22^d^ | 10 | A5-7(7-10) | 2 |  | 231-518^h^ | M60B573N02/M60B534F12 | SNP/RDP4 |
| *rbsK* | MLSA5(145) | M65B104K09^d^ | 23^i^ | ^f^(6-^f^) | ^f^ | ^f(f)^ | 231-518^h^ | M60B573N02/M60B534F12 | RDP4/CF |
| *rbsK* | MLSA5(145) | M65B63K13^d^ | 50^i^ | ^f^(5-9) | ^f^ | ^f(j)^ | 231-518^h^ | M60B573N02/M60B534F12 | RDP4 |
| *rbsK* | MLSA5(145) | M65B078P18^d^ | 36^i^ | A5-2(6-^f^) | 18 | 25^(f)^ | 231-518^h^ | M60B573N02/M60B534F12 | SNP/RDP4 |
| *rbsK* | MLSA5(145) | M60B030P24 | 32^i^ | A5-7(17-1) | 2 | 5^n^ | ^m^ | ^m^ | SNP |
| *rbsK* | MLSA5(145) | M60B066K08 | 28^i^ | A5-7(17-1) | 2 | 5 ^n^ | ^m^ | ^m^ | SNP |
| *rbsK* | MLSA5(145) | M60B183F17 | 2 | A5-7(17-1) | 2 | 5 ^n^ | ^m^ | ^m^ | SNP |
| *rbsK* | MLSA5(145) | M60B595A23 | 67^i^ | ^f^(17-1) | 1 | 7^n^ | ^m^ | ^m^ | SNP |
| *rbsK* | MLSA5(145) | M60B535B15 | 62^i^ | ^f^(17-1) | ^f^ | 6^n^ | ^m^ | ^m^ | SNP |
| *rbsK* | MLSA5(145) | M60B753F06 | 31^i^ | A5-9(17-1) | 7 | 5^n^ | ^m^ | ^m^ | SNP/CF |
| *rbsK* | MLSA5(145) | M60B086E07 | 30^i^ | ^f^(17-1) | ^f^ | 5^n^ | ^m^ | ^m^ | SNP |

Supplementary Table 10 Continued…

| **Locus** | **Alignment**  **Analysis^b^** | **Recombinant BAC** | **ST^b,e^** | **CC**  **(PE-DVST)** | **CC Cons. ST** | **# total SNP differences with cons. ST and/or (dominant variant-PE)** | **Position** | **Parents** | **Evidence^k^** |
| --- | --- | --- | --- | --- | --- | --- | --- | --- | --- |
| *rbsK* | MLSA5(145) | M60B083K10 | 29^i^ | A5-9(17-1) | 7 | 5^n^ | ^m^ | ^m^ | SNP |
| *rbsK* | MLSA5(145) | M60B782C12 | 27^i^ | ^f^(17-1) | ^f^ | 5^n^ | ^m^ | ^m^ | SNP |
| *rbsK* | MLSA5(145) | M65B151I23 | 26^i^ | A5-7(17-1) | 2 | 5^n^ | ^m^ | ^m^ | SNP |
| *rbsK* | MLSA5(145) | M60B616K18 | 13 | A5-7(17-1) | 2 | 5^n^ | ^m^ | ^m^ | SNP |
| *rbsK* | MLSA5(145) | M60B575O18 | 25^i^ | A5-7(17-1) | 2 | 5^n^ | ^m^ | ^m^ | SNP |
| *rbsK* | MLSA5(145) | M60B003C20 | 6 | A5-4(11-^f^) | 3 | 9^n^ | ^m^ | ^m^ | SNP |
| *rbsK* | MLSA5(145) | M60B463L24 | 60^i^ | A5-4(11-^f^) | 3 | 8^n^ | ^m^ | ^m^ | SNP |
| *lepB* | MLSA5(145) | M60B609H12 | 46^i^ | A5-1(9-^f^) | 17 | 14 | ^m^ | ^m^ | SNP/CF |
| *PK* | MLSA5(145) | M60B467J19 | 18 | A5-2(10-4) | 18 | 8^n,o^ | ^m^ | ^m^ | SNP |
| *PK* | MLSA5(145) | M65B053E10 | 47^i^ | A5-2(10-4) | 18 | 8^n,o^ | ^m^ | ^m^ | SNP |
| *PK* | MLSA5(145) | M65B134I01 | 4 | ^f^(10-4) | ^f^ | ^m^ | ^m^ | ^m^ | SNP/CF |
| *PK* | MLSA5(145) | M60B715I02 | 45^i^ | A5-1(10-4) | 17 | 8^n,p^ | ^m^ | ^m^ | SNP |
| *PK* | MLSA5(145) | M60B730H18 | 17 | A5-1(10-4) | 17 | 8^n,p^ | ^m^ | ^m^ | SNP |
| *lepB* | MLSA5(145) | M60B177G14 ^c^ | 43^i^ | A5-3(14-^f^) | 15 | 14^n^ | ^m^ | ^m^ | SNP |
| *rbsK, PK, aroA* | MLSA5(145) | M60B177G14 ^c^ | 43^i^ | A5-3(14-^f^) | 15 | 14^n^ | 1-1100, 2337-2760 | Unk(M65B090N22)/M60B534F14 | RDP4/SNP |
| *PK* | MLSA5(145) | M60B626L24 | 37^i^ | A5-4(13-3) | 3 | 13^n^ | ^m^ | ^m^ | SNP/CF |
| *PK* | MLSA5(145) | M60B626D13 | 24^i^ | A5-5(17-1) | 1 | 11^n^ | ^m^ | ^m^ | SNP |
| *PK* | MLSA5(145) | M60B591D09 | 66^i^ | ^f^(17-1) | ^f^ | 11^n^ | ^m^ | ^m^ | SNP |
| *PK* | MLSA5(145) | M60B534F12 | 61^i^ | ^f^(17-1) | ^f^ | 11^n^ | ^m^ | ^m^ | SNP/CF |
| *PK* | MLSA5(145) | M60B616K18 | 13 | A5-7(17-1) | 2 | 11^n^ | ^m^ | ^m^ | SNP |
| *lepB* | MLSA5(145) | M60B761P11 | 12 | A5-5(17-1) | 1 | 15^n^ | ^m^ | ^m^ | SNP |
| *CHP* | MLSA5(145) | M60B003C20 | 6 | A5-4(11-^f^) | 3 | 20^n^ | ^m^ | ^m^ | SNP |
| *CHP* | MLSA5(145) | M60B733O24 | 19 | A5-8(11-^f^) | 8 | 20^n^ | ^m^ | ^m^ | SNP |
| *rbsK* | MLSA5(145) | M65B141F14^c^ | 52 | A5-7(3-^f^) | 2 | ^m^ | 68^h^-356 | Unk(M60B373K19)/M60B626L24 | RDP4 |
| *rbsK* | MLSA5(145) | M60B504G12^d^ | 17 | A5-2(10-4) | 18 | ^m^ | 68^h^-356 | Unk(M60B373K19)/M60B626L24 | RDP4 |
| *rbsK* | MLSA5(145) | M60B609H12 ^d^ | 46 | A5-1(9-^f^) | 17 | ^m^ | 68^h^-356 | Unk(M60B373K19)/M60B626L24 | RDP4 |
| *rbsK* | MLSA5(145) | M60B580J12 ^d^ | 18 | A5-2(10-4) | 18 | ^m^ | 68^h^-356 | Unk(M60B373K19)/M60B626L24 | RDP4 |
| *rbsK* | MLSA5(145) | M60B715I02 ^d^ | 45 | A5-1(10-4) | 17 | ^m^ | 68^h^-356 | Unk(M60B373K19)/M60B626L24 | RDP4 |
| *rbsK* | MLSA5(145) | M60B730H18 ^d^ | 17 | A5-1(10-4) | 17 | ^j(m)^ | 68^h^-356 | Unk(M60B373K19)/M60B626L24 | RDP4 |
| *rbsK* | MLSA5(145) | M65B053E10 ^d^ | 47 | A5-2(10-4) | 18 | ^m^ | 68^h^-356 | Unk(M60B373K19)/M60B626L24 | RDP4 |
| *rbsK* | MLSA5(145) | M65B155E13 ^d^ | 4 | f(10-4) | ^f^ | ^f(j)^ | 68^h^-356 | Unk(M60B373K19)/M60B626L24 | RDP4 |
| *rbsK* | MLSA5(145) | M60B071F22 ^d^ | 10 | A5-7(7-10) | 2 | ^m^ | 68^h^-356 | Unk(M60B373K19)/M60B626L24 | RDP4 |
| *rbsK* | MLSA5(145) | M60B207O10 ^d^ | 9 | ^f^(5-9) | ^f^ | ^f(j)^ | 68^h^-356 | Unk(M60B373K19)/M60B626L24 | RDP4 |
| *rbsK* | MLSA5(145) | M60B349M23 ^d^ | 9 | ^f^(5-9) | ^f^ | ^f(j)^ | 68^h^-356 | Unk(M60B373K19)/M60B626L24 | RDP4 |
| *rbsK* | MLSA5(145) | M60B547N16 ^d^ | 63 | ^f^(5-9) | ^f^ | ^j(m)^ | 68^h^-356 | Unk(M60B373K19)/M60B626L24 | RDP4 |

Supplementary Table 10 Continued…

| *rbsK* | MLSA5(145) | M60B691E09 ^d^ | 51 | ^f^(4-^f^) | ^f^ | ^f(f)^ | 68^h^-356 | Unk(M60B373K19)/M60B626L24 | RDP4 |
| --- | --- | --- | --- | --- | --- | --- | --- | --- | --- |
| *rbsK* | MLSA5(145) | M65B038L09 ^d^ | 53 | ^f^(3-^f^) | ^f^ | ^f(f)^ | 68^h^-356 | Unk(M60B373K19)/M60B626L24 | RDP4 |
| *rbsK* | MLSA5(145) | M65B063K13 ^d^ | 50 | ^f^(5-9) | ^f^ | ^j(m)^ | 68^h^-356 | Unk(M60B373K19)/M60B626L24 | RDP4 |
| *rbsK* | MLSA5(145) | M65B080L10 ^d^ | 54 | ^f^(3-^f^) | ^f^ | ^f(f)^ | 68^h^-356 | Unk(M60B373K19)/M60B626L24 | RDP4 |
| *rbsK* | MLSA5(145) | M65B102K09 ^d^ | 23 | ^f^(6-^f^) | ^f^ | ^f(f)^ | 68^h^-356 | Unk(M60B373K19)/M60B626L24 | RDP4 |
| *rbsK* | MLSA5(145) | M65B078P18 ^d^ | 36 | A5-2(6-^f^) | 18 | ^m^ | 68^h^-356 | Unk(M60B373K19)/M60B626L24 | RDP4 |
| *rbsK, PK, CHP, aroA* | MLSA5(145) | M60B626D13^c^ | 24 | A5-5(17-1) | 1 | ^m^ | 1-780, 1690-^h^ | Unk(M65B019P07)/M60B740F14 | RDP4 |
| *PK, lepB, CHP, aroA* | MLSA5(145) | M60B595E04 ^c^ | 57 | ^f^(7-10) | ^f^ | ^j(m)^ | 472-^h^ | M60B691E09/M60B609H12 | RDP4 |
| *lepB, CHP, aroA* | MLSA5(145) | M65B150M20 ^c^ | 44 | ^f^(19-^f^) | ^f^ | ^f(f)^ | 1133-^h^ | Unk(M60B740F14)/M60B051K07 | RDP4 |
| *rbsK* | MLSA5(145) | M65B150M20 ^c^ | 44 | ^f^(19-^f^) | ^f^ | ^f(f)^ | 156-^h^ | M60B379G20/M65B035K07 | RDP4 |
| *lepB, CHP* | MLSA5(145) | M60B573N02 ^c^ | 65 | ^f^(1-^f^) | ^f^ | ^f(f)^ | 1110-2138^h^ | Unk(M60B761P11)/M60B503C09 | RDP4 |
| *rbsK* | MLSA5(145) | M60B573N02 ^c^ | 65 | ^f^(1-^f^) | ^f^ | ^f(f)^ | 78^h^-228 | M60B373K19/M60B379G20 | RDP4 |
| *rbsK* | MLSA5(49) | M65B149H05^c,g^ | 2 | ^f^(8-2) | ^f^ | ^f^(^j^) | ^h^ | OSA/M65B053E10 | RDP4 |
| *rbsK* | MLSA5(49) | M65B093I20^d^ | 2 | ^f^(8-2) | ^f^ | ^f^(^j^) | ^h^ | OSA/M65B053E10 | RDP4 |
| *rbsK* | MLSA5(49) | M65B134I01^d^ | 2 | ^f^(8-2) | ^f^ | ^f^(^j^) | ^h^ | OSA/M65B053E10 | RDP4 |
| *rbsK* | MLSA5(49) | M65B145I07^d^ | 2 | ^f^(8-2) | ^f^ | ^f^(^j^) | ^h^ | OSA/M65B053E10 | RDP4 |
| *rbsK* | MLSA5(49) | M65B155E13^d^ | 2 | ^f^(8-2) | ^f^ | ^f^(^j^) | ^h^ | OSA/M65B053E10 | RDP4 |
| *rbsK* | MLSA5(49) | M60B065P14 ^d^ | 4 | A5-I(13-1) | 1 | ^m^ | 2458-^h^ | M65B141F14/M65B093I20 | RDP4 |
| *rbsK* | MLSA5(49) | M60B166J20 ^d^ | 1 | A5-I(13-1) | 1 | ^j(j)^ | 2458-^h^ | M65B141F14/M65B093I20 | RDP4 |
| *rbsK* | MLSA5(49) | M60B183F17 ^d^ | 3 | A5-ii(10-3) | 3 | ^j(j)^ | 2458-^h^ | M65B141F14/M65B093I20 | RDP4 |
| *rbsK* | MLSA5(49) | M60B373K19 ^d^ | 8 | A5-I(13-1) | 1 | ^m^ | 2458-^h^ | M65B141F14/M65B093I20 | RDP4 |
| *rbsK* | MLSA5(49) | M60B374C09 ^d^ | 1 | A5-I(13-1) | 1 | ^j(j)^ | 2458-^h^ | M65B141F14/M65B093I20 | RDP4 |
| *rbsK* | MLSA5(49) | M60B405K13d | 1 | A5-I(13-1) | 1 | ^j(j)^ | 2458-^h^ | M65B141F14/M65B093I20 | RDP4 |
| *rbsK* | MLSA5(49) | M60B436G19 ^c^ | 26 | ^f^(9-5) | ^f^ | ^f^ | 2458-^h^ | M65B141F14/M65B093I20 | RDP4 |
| *rbsK* | MLSA5(49) | M60B443F23 ^d, l^ | 5 | A5-i(9-5) | 5 | ^j(j)^ | 2458- ^h^ | M65B141F14/M65B093I20 | RDP4 |
| *rbsK* | MLSA5(49) | M60B468P07 ^d^ | 6 | A5-I(13-1) | 1 | ^m^ | 2458- ^h^ | M65B141F14/M65B093I20 | RDP4 |
| *rbsK* | MLSA5(49) | M60B491M01 ^d^ | 6 | A5-I(13-1) | 1 | ^m^ | 2458- ^h^ | M65B141F14/M65B093I20 | RDP4 |
| *rbsK* | MLSA5(49) | M60B501M02 ^d^ | 1 | A5-I(13-1) | 1 | ^j(j)^ | 2458- ^h^ | M65B141F14/M65B093I20 | RDP4 |
| *rbsK* | MLSA5(49) | M60B514A21 ^d^ | 1 | A5-I(13-1) | 1 | ^j(j)^ | 2458- ^h^ | M65B141F14/M65B093I20 | RDP4 |
| *rbsK* | MLSA5(49) | M60B527I20 ^d^ | 3 | A5-ii(10-3) | 3 | ^j(j)^ | 2458- ^h^ | M65B141F14/M65B093I20 | RDP4 |
| *rbsK* | MLSA5(49) | M60B559P01 ^d^ | 1 | A5-I(13-1) | 1 | ^j(j)^ | 2458- ^h^ | M65B141F14/M65B093I20 | RDP4 |
| *rbsK* | MLSA5(49) | M60B560H17 ^d^ | 4 | A5-I(13-1) | 1 | ^m^ | 2458- ^h^ | M65B141F14/M65B093I20 | RDP4 |
| *rbsK* | MLSA5(49) | M60B563B21 ^d,l^ | 5 | A5-i(9-5) | 5 | ^j(j)^ | 2458- ^h^ | M65B141F14/M65B093I20 | RDP4 |
| *rbsK* | MLSA5(49) | M60B574L23 ^d^ | 1 | A5-I(13-1) | 1 | ^j(j)^ | 2458- ^h^ | M65B141F14/M65B093I20 | RDP4 |
| *rbsK* | MLSA5(49) | M60B651G15 ^d^ | 1 | A5-I(13-1) | 1 | ^j(j)^ | 2458- ^h^ | M65B141F14/M65B093I20 | RDP4 |
| *rbsK* | MLSA5(49) | M60B653P08 ^d^ | 14 | A5-i(9-5) | 5 | ^m^ | 2458- ^h^ | M65B141F14/M65B093I20 | RDP4 |
| *rbsK* | MLSA5(49) | M60B689K07 ^d^ | 1 | A5-I(13-1) | 1 | ^j(j)^ | 2458- ^h^ | M65B141F14/M65B093I20 | RDP4 |
| *rbsK* | MLSA5(49) | M60B773C01 ^d^ | 1 | A5-I(13-1) | 1 | ^j(j)^ | 2458- ^h^ | M65B141F14/M65B093I20 | RDP4 |
| *rbsK* | MLSA5(49) | M60B788A07 ^d^ | 1 | A5-I(13-1) | 1 | ^j(j)^ | 2458- ^h^ | M65B141F14/M65B093I20 | RDP4 |

Supplementary Table 10 Continued…

| *rbsK* | MLSA5(49) | M65B009D20 ^d^ | 3 | A5-ii(10-3) | 3 | ^j(j)^ | 2458-^h^ | M65B141F14/M65B093I20 | RDP4 |
| --- | --- | --- | --- | --- | --- | --- | --- | --- | --- |
| *rbsK* | MLSA5(49) | M65B010I14 ^d^ | 1 | A5-I(13-1) | 1 | ^j(j)^ | 2458-^h^ | M65B141F14/M65B093I20 | RDP4 |
| *rbsK* | MLSA5(49) | M65B025J23 ^d^ | 3 | A5-ii(10-3) | 3 | ^j(j)^ | 2458-^h^ | M65B141F14/M65B093I20 | RDP4 |
| *rbsK* | MLSA5(49) | M65B032B16 ^d, l^ | 16 | A5-i(9-5) | 5 | ^m^ | 2458-^h^ | M65B141F14/M65B093I20 | RDP4 |
| *rbsK* | MLSA5(49) | M65B035K07 ^d, l^ | 15 | ^f^ | ^f^ | ^f^ | 2458-^h^ | M65B141F14/M65B093I20 | RDP4 |
| *rbsK* | MLSA5(49) | M65B090N22 ^d^ | 20 | ^f^ | ^f^ | ^f^ | 2458-^h^ | M65B141F14/M65B093I20 | RDP4 |
| *rbsK* | MLSA5(49) | M65B107B14 ^d^ | 1 | A5-I(13-1) | 1 | ^j(j)^ | 2458-^h^ | M65B141F14/M65B093I20 | RDP4 |
| *rbsK* | MLSA5(49) | M65B111J13 ^d, l^ | 18 | ^f^ | ^f^ | ^f^ | 2458-^h^ | M65B141F14/M65B093I20 | RDP4 |
| *rbsK* | MLSA5(49) | M65B115H01 ^d, l^ | 21 | ^f^ | ^f^ | ^f^ | 2458-^h^ | M65B141F14/M65B093I20 | RDP4 |
| *rbsK* | MLSA5(49) | M65B115I21 ^d^ | 22 | ^f^ | ^f^ | ^f^ | 2458-^h^ | M65B141F14/M65B093I20 | RDP4 |
| *lepB, PK* | MLSA5(49) | M60B609H12^c^ | 12 | ^f^(6-^f^) | ^f^ | ^f(f)^ | 1242-2151^h^ | M65B104K09/Unk(M60B436G19) | RDP4 |
| *lepB, PK* | MLSA5(49) | M60B595E04^d^ | 13 | ^f^(2-^f^) | ^f^ | ^f^ | 1242-2151^h^ | M65B104K09/Unk(M60B436G19) | RDP4 |
| *CHP, lepB* | MLSA5(49) | M60B653P08 ^c^ | 14 | A5-i(9-5) | 5 | ^m^ | 710^h^-1432 | M65B078P18/Unk(M65B115I21) | RDP4 |
| *rbsK* | MLSA5(49) | M65B150M20 ^d^ | 17 | ^f^(8-2) | ^f^ | ^f^(27) | ^h^ | OSA/M65B053E10 | RDP4 |
| *rbsK* | MLSA7 | M60B504G12^c^ | 35 | ^f^(9-^f^) | ^f^ | ^f(f)^ | 207-358 | M60B595E04^g^ /M60B503C09 | RDP4/CF |
| *rbsK* | MLSA7 | M60B609H12^d^ | 34 | ^f^(9-^f^) | ^f^ | ^f(f)^ | 207-358 | M60B595E04 ^g^ /M60B503C09 | RDP4/CF |
| *rbsK* | MLSA7 | M60B715I02 ^d^ | 24 | ^f^(9-^f^) | ^f^ | ^f(f)^ | 207-358 | M60B595E04 ^g^ /M60B503C09 | RDP4/CF |
| *lepB* | MLSA7 | M60B595E04 | 27 | ^f^ | ^f^ | ^f(f)^ | ^m^ | ^m^ | SNP/CF |
| *lepB* | MLSA7 | M60B177G14 | 25 | ^f^ | ^f^ | ^f(f)^ | ^m^ | ^m^ | SNP/CF |
| *dnaG* | MLSA7 | M60B177G14 | 25 | ^f^ | ^f^ | ^f(f)^ | ^m^ | ^m^ | SNP/CF |
| *dnaG* | MLSA7 | M60B589H13c | 12 | A7-I(8-^f^) | ^1^ | 19^n^ | ^m^ | ^m^ | SNP/CF |
| *CHP, aroA* | MLSA7 | M60B755A11c | 11 | A7-II(4-^f^) | ^5^ | 20^n^ | 2319-3106^h^ | M60B183F17/Unk (M60B595E04) | RDP4/SNP/CF |
| *CHP, aroA* | MLSA7 | M60B725H17 | 18 | A7-II(4-^f^) | ^5^ | ^m(f)^ | 2319-3106^h^ | M60B183F17/Unk (M60B595E04) | RDP4/SNP/CF |
| *hisF, lepB* | MLSA7 | M60B177G14 | 27 | ^f^(2-^f^) | ^f^ | ^f(f)^ | ^h^ | M60B195D22/ Unk (M65B080L10) | RDP4/SNP/CF |
| *CHP, aroA* | MLSA7 | M60B177G14 | 27 | ^f^(2-^f^) | ^f^ | ^f(f)^ | 2319-3106^h^ | M60B183F17/Unk (M60B595E04) | RDP4/SNP/CF |
| *CHP, aroA* | MLSA7 | M60B503C09 | 29 | ^f^(3-^f^) | ^f^ | ^f(f)^ | 2319-3106^h^ | M60B183F17/Unk (M60B595E04) | RDP4/SNP/CF |
| *CHP, aroA* | MLSA7 | M60B478F03 | 30 | ^f^(3-^f^) | ^f^ | ^f(f)^ | 2319-3106^h^ | M60B183F17/Unk (M60B595E04) | RDP4/SNP/CF |
| *CHP, aroA* | MLSA7 | M60B443F23 | 21 | A7-II(4-^f^) | ^5^ | ^m(f)^ | 2319-3106^h^ | M60B183F17/Unk (M60B595E04) | RDP4/SNP/CF |
| *CHP, aroA* | MLSA7 | M60B463L24 | 45 | A7-II(4-^f^) | ^5^ | ^m(f)^ | 2319-3106^h^ | M60B183F17/Unk (M60B595E04) | RDP4/SNP/CF |
| *CHP, aroA* | MLSA7 | M60B541E20 | 46 | A7-II(4-^f^) | ^5^ | ^m(f)^ | 2319-3106^h^ | M60B183F17/Unk (M60B595E04) | RDP4/SNP/CF |
| *CHP, aroA* | MLSA7 | M60B563B21 | 31 | A7-II(4-^f^) | ^5^ | ^m(f)^ | 2319-3106^h^ | M60B183F17/Unk (M60B595E04) | RDP4/SNP/CF |
| *CHP, aroA* | MLSA7 | M60B195D22 | 19 | A7-II(4-^f^) | ^5^ | ^m(f)^ | 2319-3106^h^ | M60B183F17/Unk (M60B595E04) | RDP4/SNP/CF |
| *CHP, aroA* | MLSA7 | M60B653P08 | 7 | A7-II(4-^f^) | ^6^ | 20^n^ | 2319-3106^h^ | M60B183F17/Unk (M60B595E04) | RDP4/SNP/CF |
| *CHP, aroA* | MLSA7 | M60B733O24 | 7 | A7-II(4-^f^) | ^6^ | 20^n^ | 2319-3106^h^ | M60B183F17/Unk (M60B595E04) | RDP4/SNP/CF |
| *CHP, aroA* | MLSA7 | M60B399F15 | 5 | A7-II(4-^f^) | ^5^ | ^j(f)^ | 2319-3106^h^ | M60B183F17/Unk (M60B595E04) | RDP4/SNP/CF |
| *CHP, aroA* | MLSA7 | M60B461H19 | 5 | A7-II(4-^f^) | ^5^ | ^j(f)^ | 2319-3106^h^ | M60B183F17/Unk (M60B595E04) | RDP4/SNP/CF |

Supplementary Table 10 Continued…

| *CHP, aroA* | MLSA7 | M60B247G10 | 16 | ^f^(4-^f^) | ^f^ | ^f(f)^ | 2319-3106^h^ | M60B183F17/Unk (M60B595E04) | RDP4/SNP/CF |
| --- | --- | --- | --- | --- | --- | --- | --- | --- | --- |
| *CHP, aroA* | MLSA7 | M60B499M16 | 28 | ^f^(3-^f^) | ^f^ | ^f(f)^ | 2319-3106^h^ | M60B183F17/Unk (M60B595E04) | RDP4/SNP/CF |
| *CHP, aroA* | MLSA7 | M60B436G19 | 44 | ^f^(3-^f^) | ^f^ | ^f(f)^ | 2319-3106^h^ | M60B183F17/Unk (M60B595E04) | RDP4/SNP/CF |
| *rbsK-aroA* | MLSA7 | M60B589H13 | 12 | A7-I(8-^f^) | 1 | ^m(f)^ | ^h^ | Unk(M65B090N22)/ M60B559P01 | RDP4 |
| *PK* | MLSA7 | M60B626D13 | 15 | A7-I(7-1) | 1 | 11^n^ | ^m^ | ^m^ | SNP |
| *lepB* | MLSA7 | M60B740F14 | 4 | A7-I(1-^f^) | 1 | 13^n^ | ^h^ | M65B090N22/M60B177G14 | RDP4/SNP/CF |
| *hisF, CHP, aroA* | MLSA7 | M60B740F14 | 4 | A7-I(1-^f^) | 1 | 13^n^ | 1386-3382^h^ | M65B090N22/M60B177G14 | RDP4 |

*Abbreviations*: **MLSA5(145):** 5-locus A-like *Synechococcus*  population alignment of 145 sequences. **MLSA5(49):** 5-locus A-like *Synechococcus*  population alignment of 49 sequences. **MLSA7:** 7-locus A-like *Synechococcus*  population alignment. **BAC**: Bacterial Artificial Chromosome. **PE:** putative ecotype. **ST**: sequence type. **DV**: dominant variant. **CC**: clonal complex. **Cons**: Clonal complex consensus sequence type. **SNP**: single nucleotide polymorphism. **CF**: ClonalFrame analysis ([4](#_ENREF_4)).

^a^ Genome annotation available at: <http://cmr.jcvi.org/cgi-bin/CMR/GenomePage.cgi?org=gyma>

^b^ For corresponding ST/allelic profile tables: see Supplementary Table 8 (MLSA7), Supplementary Table 19 (MLSA5(49)) and Supplementary Table 20 (MLSA5(145)).

^c^ RDP4-defined recombinant

^d^ Sequence identified as having the evidence of the recombination event as an RDP4-defined recombinant

^e^ ST defined from 5-locus A-like *Synechococcus*  population analyses and does not equate with ST designation from the 7-

locus analysis. Please refer to ‘alignment analysis’ column and refer to footnote b.

^f^ Not found within a clonal complex or not found in a PE clade that contains a dominant variant

^g^ RDP4 cautions that the parent(s) and recombinant may be reversed.

^h^ RDP4 software unsure of beginning or ending breakpoint site of recombination

^i^  Sequence type represented by 1 sequence

^j^ Sequence type was the clonal complex consensus sequence or dominant PE-ST.

^k^ p-value cut off in RDP4 analysis, p < 0.05. P-values can be found in Supplementary Table 12.

^l^ Also shared *CHP/lepB* recombination signal with M60B653P08

^m^ Not applicable, not determined

^n^ Number of SNPs at locus only as compared to dominant variant or consensus group, see SNP maps; Main Text Fig 5 and Supplementary Figure 21.

^o^ As compared to consensus ST17

^p^ As compared to consensus ST18

^q^ All putative recombinants confirmed via RDP4 analysis or occur on terminal branches of phylogeny (main-text Fig 2) and confirmed via SNP and/or CF analysis.

## Supplementary Table 11. Analysis of recombination signals in B´-like *Synechococcus* BAC sequences.

| **Locus** | **Alignment**  **Analysis^b^** | **Recombinant BAC** | **ST^b^** | **CC**  **(PE-DVST)** | **CC Cons. ST** | **# total SNP differences with cons. ST and/or (dominant variant-PE)** | **Position** | **Parents** | **Evidence^k,q^** |
| --- | --- | --- | --- | --- | --- | --- | --- | --- | --- |
| *rbsK* | MLSA4 | M60B579B21^c^ | 46 | ^f^(26-^f^) | ^f^ | ^f(f)^ | 568-928 | M60B594L19/OSA | RDP4/CF |
| *rbsK* | MLSA4 | M60B085O17 ^d^ | 3 | B4-II(27 ^p^ -^f^) | 6 | ^m(f)^ | 568-928 | M60B594L19/OSA | RDP4/CF |
| *rbsK* | MLSA4 | M60B057N07 ^d^ | 3 | B4-II(27 ^p^ -^f^) | 6 | ^m(f)^ | 568-928 | M60B594L19/OSA | RDP4/CF |
| *rbsK* | MLSA4 | M60B626O23 ^d^ | 3 | B4-II(27 ^p^ -^f^) | 6 | ^m(f)^ | 568-928 | M60B594L19/OSA | RDP4/CF |
| *rbsK* | MLSA4 | M60B714K10 ^d^ | 3 | B4-II(27 ^p^ -^f^) | 6 | ^m(f)^ | 568-928 | M60B594L19/OSA | RDP4/CF |
| *rbsK* | MLSA4 | M60B700J15 ^d^ | 15 | ^f^ (28- ^f^) | ^f^ | ^f(f)^ | 568-928 | M60B594L19/OSA | RDP4/CF |
| *rbsK* | MLSA4 | M60B499C24^d,l^ | 35 | ^o^(25-^f^) | ^f^ | ^f(f)^ | 568-928 | M60B594L19/OSA | RDP4/CF |
| *rbsK* | MLSA4 | M60B129N02^d,l^ | 19 | ^f^(25-^f^) | ^f^ | ^f(f)^ | 568-928 | M60B594L19/OSA | RDP4/CF |
| *rbsK* | MLSA4 | M60B426N01^d,l^ | 36 | ^o^(25-^f^) | ^f^ | ^f(f)^ | 568-928 | M60B594L19/OSA | RDP4/CF |
| *rbsK* | MLSA4 | M60B403F16^d,l^ | 51 | ^o^(25-^f^) | ^f^ | ^f(f)^ | 568-928 | M60B594L19/OSA | RDP4/CF |
| *rbsK* | MLSA4 | M60B541D06^d^ | 13 | ^f^(3-^f^) | ^f^ | ^f(f)^ | 568-928 | M60B594L19/OSA | RDP4/CF |
| *rbsK* | MLSA4 | M60B769K22 ^e^ | 2 | ^f^(22 ^p^ -2) | ^f^ | ^f(f)^ | 568-928 | M60B594L19/OSA | RDP4 |
| *rbsK* | MLSA4 | M60B081O16^e^ | 48 | ^f^(23-^f^) | ^f^ | ^f(f)^ | 568-928 | M60B594L19/OSA | RDP4/CF |
| *rbsK* | MLSA4 | M60B046B18^e^ | 41 | ^f^(24-^f^) | ^f^ | ^f(f)^ | 568-928 | M60B594L19/OSA | RDP4 |
| *rbsK* | MLSA4 | M60B091H06^e^ | 2 | ^f^(22 ^p^ -2) | ^f^ | ^f(j)^ | 568-928 | M60B594L19/OSA | RDP4/CF |
| *rbsK* | MLSA4 | M60B648C08 ^e^ | 2 | ^f^(22 ^p^ -2) | ^f^ | ^f(f)^ | 568-928 | M60B594L19/OSA | RDP4 |
| *rbsK* | MLSA4 | M60B513G21 ^e^ | 2 | ^f^(22 ^p^ -2) | ^f^ | ^f(f)^ | 568-928 | M60B594L19/OSA | RDP4 |
| *rbsK* | MLSA4 | M60B186K12 ^e^ | 2 | ^f^(22 ^p^ -2) | ^f^ | ^f(f)^ | 568-928 | M60B594L19/OSA | RDP4 |
| *rbsK* | MLSA4 | M60B456A21^e^ | 17 | ^f^(22 ^p^ -2) | ^f^ | ^f^(3) | 568-928 | M60B594L19/OSA | RDP4/CF |
| *rbsK* | MLSA4 | M60B477G06^e^ | 49 | ^f^(20-^f^) | ^f^ | ^f(f)^ | 568-928 | M60B594L19/OSA | RDP4/CF |
| *rbsK* | MLSA4 | M60B397N23^e^ | 18 | ^f^(21-^f^) | ^f^ | ^f(f)^ | 568-928 | M60B594L19/OSA | RDP4/CF |
| *rbsK* | MLSA4 | M60B347P16 ^e^ | 2 | ^f^(22 ^p^ -2) | ^f^ | ^f(f)^ | 568-928 | M60B594L19/OSA | RDP4/CF |
| *rbsK* | MLSA4 | M60B250B15^e^ | 42 | ^f^(19-^f^) | ^f^ | ^f(f)^ | 568-928 | M60B594L19/OSA | RDP4 |
| *rbsK* | MLSA4 | M60B456P18^c^ | 30 | B4-III(15- ^f^) | 5 | ^m(f)^ | 636-1085 | M60B623B05/M60B075B13 | RDP4 |
| *rbsK* | MLSA4 | M60B041P24 ^e^ | 16 | ^f^(8- ^f^) | ^f^ | ^f(f)^ | 636-1085 | M60B623B05/M60B075B13 | RDP4 |
| *rbsK* | MLSA4 | M60B477G06 ^e^ | 49 | ^f^ (20- ^f^) | ^f^ | ^f(f)^ | 636-1085 | M60B623B05/M60B075B13 | RDP4 |
| *rbsK* | MLSA4 | M60B397N23 ^e^ | 18 | ^f^(21-^f^) | ^f^ | ^f(f)^ | 636-1085 | M60B623B05/M60B075B13 | RDP4 |
| *rbsK* | MLSA4 | M60B090B21 ^e^ | 39 | ^f^ (13- ^f^) | ^f^ | ^f(f)^ | 636-1085 | M60B623B05/M60B075B13 | RDP4 |
| *rbsK* | MLSA4 | M60B250B15 ^e^ | 42 | ^f^ (19- ^f^) | ^f^ | ^f(f)^ | 636-1085 | M60B623B05/M60B075B13 | RDP4 |
| *rbsK* | MLSA4 | M60B579B21 ^e^ | 46 | ^f^ (26- ^f^) | ^f^ | ^f(f)^ | 636-1085 | M60B623B05/M60B075B13 | RDP4 |
| *rbsK* | MLSA4 | M60B499C24 ^e^ | 35 | ^f^ (25- ^f^) | ^f^ | ^f(f)^ | 636-1085 | M60B623B05/M60B075B13 | RDP4 |
| *rbsK* | MLSA4 | M60B129N02 ^e^ | 19 | ^f^ (25- ^f^) | ^f^ | ^f(f)^ | 636-1085 | M60B623B05/M60B075B13 | RDP4 |
| *rbsK* | MLSA4 | M60B426N01 ^e^ | 36 | ^f^ (25- ^f^) | ^f^ | ^f(f)^ | 636-1085 | M60B623B05/M60B075B13 | RDP4 |
| *rbsK* | MLSA4 | M60B403F16 ^e^ | 51 | ^f^ (25- ^f^) | ^f^ | ^f(f)^ | 636-1085 | M60B623B05/M60B075B13 | RDP4 |
| *rbsK/pcrA* | MLSA4 | M60B115G12^c^ | 27 | B4-II(6 ^p^ -6) | 6 | ^m(f)^ | ^h^ | M60B700J15/OSA | RDP4 |
| *rbsK/pcrA* | MLSA4 | M60B455A17^d^ | 25 | B4-II(6 ^p^ -6) | 6 | ^m(f)^ | ^h^ | M60B700J15/OSA | RDP4 |
| *rbsK/pcrA* | MLSA4 | M60B554K06 ^d^ | 6 | B4-II(6 ^p^ -6) | 6 | ^j(f)^ | ^h^ | M60B700J15/OSA | RDP4 |
| *rbsK/pcrA* | MLSA4 | M60B420G12 ^d^ | 24 | B4-II(6 ^p^ -6) | 6 | ^m(f)^ | ^h^ | M60B700J15/OSA | RDP4 |

Supplementary Table 11 Continued…

| **Locus** | **Alignment**  **Analysis^b^** | **Recombinant BAC** | **ST^b^** | **CC**  **(PE-DVST)** | **CC Cons. ST** | **# total SNP differences with cons. ST and/or (dominant variant-PE)** | **Position** | **Parents** | **Evidence^k,q^** |
| --- | --- | --- | --- | --- | --- | --- | --- | --- | --- |
| *aroA* | MLSA4 | M60B421K10 | 50 | ^f^(14-^f^) | ^f^ | ^f(f)^ | ^m^ | ^m^ | SNP/CF |
| *rbsK/pcrA* | MLSA4 | M60B078P12 ^d^ | 6 | B4-II(6 ^p^ -6) | 6 | ^j(f)^ | ^h^ | M60B700J15/OSA | RDP4 |
| *rbsK/pcrA* | MLSA4 | M60B674F15 ^d^ | 26 | B4-II(6 ^p^ -6) | 6 | ^m(f)^ | ^h^ | M60B700J15/OSA | RDP4 |
| *rbsK/pcrA* | MLSA4 | M60B754M13 ^d^ | 28 | B4-II(6 ^p^ -6) | 6 | ^m(f)^ | ^h^ | M60B700J15/OSA | RDP4 |
| *rbsK/pcrA* | MLSA4 | M60B450N20 ^d^ | 29 | B4-II(6 ^p^-6) | 6 | ^m(f)^ | ^h^ | M60B700J15/OSA | RDP4 |
| *16SrRNA/ITS* | MLSA4 | M60B614C12 | 8 | B4-I(1-1) | 1 | 5^n^ | ^m^ | ^m^ | SNP |
| *16SrRNA/ITS* | MLSA4 | M60B018J02 | 9 | B4-I(2-^f^) | 1 | 24^n^ | ^m^ | ^m^ | SNP/CF |
| *16SrRNA/ITS* | MLSA4 | M60B041O05 | 10 | B4-I(2-^f^) | 1 | 13^n^ | ^m^ | ^m^ | SNP/CF |
| *16SrRNA/ITS* | MLSA4 | M60B100J22 | 11 | B4-I(2-^f^) | 1 | 5^n^ | ^m^ | ^m^ | SNP/CF |
| *pcrA* | MLSA4 | M60B541D06^c^ | 13 | ^f^(3-^f^) | ^f^ | ^f(f)^ | ^m^ | ^m^ | SNP/CF |
| *pcrA* | MLSA4 | M60B468M14 | 14 | ^f^ (4- ^f^) | ^f^ | ^f(f)^ | ^m^ | ^m^ | SNP/CF |
| *pcrA* | MLSA4 | M60B700J15 | 15 | ^f^ (28- ^f^) | ^f^ | ^f(f)^ | ^m^ | ^m^ | SNP |
| *rbsK* | MLSA4 | M60B041P24 | 16 | ^f^ (8- ^f^) | ^f^ | ^f(f)^ | ^m^ | ^m^ | SNP |
| *pcrA* | MLSA4 | M60B041P24 | 16 | ^f^ (8- ^f^) | ^f^ | ^f(f)^ | ^m^ | ^m^ | SNP |
| *pcrA* | MLSA4 | M60B397N23^d^ | 18 | ^f^(21-^f^) | ^f^ | ^f(f)^ | ^m^ | ^m^ | SNP/CF |
| *16SrRNA* | MLSA4 | M60B397N23^d^ | 18 | ^f^(21-^f^) | ^f^ | ^f(f)^ | ^m^ | ^m^ | SNP/CF |
| *16SrRNA* | MLSA4 | M60B129N02^e,l^ | 19 | ^f^(25-^f^) | ^f^ | ^f(f)^ | ^m^ | ^m^ | SNP/CF |
| *rbsK* | MLSA4 | M60B772E10 | 20 | ^f^(17-^f^) | ^f^ | ^f(f)^ | ^m^ | ^m^ | SNP/CF |
| *rbsK* | MLSA4 | M60B433E13 | 23 | ^f^(9-^f^) | ^f^ | ^f(f)^ | ^m^ | ^m^ | SNP/CF |
| *rbsK* | MLSA4 | M60B456P18 | 30 | B4-III(15-^f^) | 5 | 14^n^ | ^m^ | ^m^ | SNP/CF |
| *16SrRNA/ITS* | MLSA4 | M60B623N20 | 34 | B4-III(16-5) | 5 | 9^n^ | ^m^ | ^m^ | SNP/CF |
| *16SrRNA/ITS* | MLSA4 | M60B420G12 | 24 | B4-II(7- ^f^) | 6 | ^m(f)^ | 2252- ^h^ | M60B674F15/OSA | RDP4 |
| *16SrRNA/ITS* | MLSA4 | M60B397N23 | 18 | ^f^ (1-1) | ^f^ | ^f(m)^ | 2252- ^h^ | M60B674F15/OSA | RDP4 |
| *16SrRNA/ITS* | MLSA4 | M60B400M20 | 32 | B4-III (16-5) | 5 | ^m(m)^ | 2252-^h^ | M60B674F15/OSA | RDP4 |
| *16SrRNA* | MLSA4 | M60B081I21 | 38 | ^f^ (5- ^f^) | ^f^ | ^f(f)^ | ^m^ | ^m^ | SNP/CF |
| *16SrRNA* | MLSA4 | M60B046B18^d^ | 41 | ^f^(24-^f^) | ^f^ | ^f(f)^ | ^m^ | ^m^ | SNP/CF |
| *pcrA* | MLSA4 | M60B250B15^d^ | 42 | ^f^(19-^f^) | ^f^ | ^f(f)^ | ^m^ | ^m^ | SNP/CF |
| *16SrRNA* | MLSA4 | M60B015H24 | 43 | ^f^(29-^f^) | ^f^ | ^f(f)^ | ^m^ | ^m^ | SNP |
| *aroA* | MLSA4 | M60B015H24 | 43 | ^f^(29-^f^) | ^f^ | ^f(f)^ | ^m^ | ^m^ | SNP/CF |
| *aroA* | MLSA4 | M60B579B21^e^ | 46 | ^f^(26-^f^) | ^f^ | ^f(f)^ | ^m^ | ^m^ | SNP |
| *pcrA* | MLSA4 | M60B075B13 | 47 | ^f^(11-^f^) | ^f^ | ^f(f)^ | ^m^ | ^m^ | SNP/CF |
| *aroA* | MLSA4 | M60B075B13 | 47 | ^f^(11-^f^) | ^f^ | ^f(f)^ | ^m^ | ^m^ | SNP/CF |

*Abbreviations*: **MLSA4:** 4-locus *Synechococcus*  sp. B´-like population alignment. BAC: Bacterial Artificial Chromosome. **PE:** putative ecotype. **ST**: sequence type. **DV**: dominant variant. **CC**: clonal complex. **Cons**: Clonal complex consensus sequence type. **SNP**: single nucleotide polymorphism. **CF**: ClonalFrame analysis ([4](#_ENREF_4)).

^a^ Genome annotation available at: <http://cmr.jcvi.org/cgi-bin/CMR/GenomePage.cgi?org=gymb>.

^b^ For corresponding ST table: see Supplementary Table 9.

^c^ RDP4-defined recombinant

^d^ Sequence identified as having the same recombination event as an RDP4-defined recombinant

^e^ Sequence identified as having partial or trace evidence of recombination event as an RDP4-defined recombinant.

^f^ Not found within a clonal complex or not found in a PE clade that contains a dominant variant

^g^ RDP4 cautions that the parent(s) and recombinant may be reversed.

^h^ RDP4 software unsure of beginning and/or ending breakpoint site of recombination

^i^  Sequence type represented by 1 sequence

^j^ Sequence type was the clonal complex consensus sequence or dominant PE-ST.

^k^ p-value cut off in RDP4 analysis, p < 0.05. P-values can be found in Supplementary Table 13.

^l^ Also contained another significant *rbsK* recombination signal at position 215-364, shared with RDP4-defined recombinant M60B541D06, parents M60B400M20/M60B700J15.

^m^ Not applicable, not determined

^n^ Number of SNPs at locus only as compared to dominant variant or consensus group, see SNP maps; Supplementary Figures 12 and 13.

^o^ Would be contained within a clonal complex if criteria were relaxed to allow for 2 single locus variants. See Supplementary Figure 11.

^p^ All variants within PE contain putative recombination event.

^q^ All putative recombinants confirmed via RDP4 analysis or occur on terminal branches of phylogeny (Supplementary Figure 10) and confirmed via SNP and/or CF analysis.

## Supplementary Table 12. P-values for RDP4 analysis of recombinants for A-like *Synechococcus* BACs^++^. Corresponds to Supplementary Table 10.

| **Locus** | **Alignment**  **Analysis** | **Recombinant BAC** | **MaxChi** | **GENECONV** | **RDP** | **Chimaera** | **Siscan** | **3Seq** | **LARD** |
| --- | --- | --- | --- | --- | --- | --- | --- | --- | --- |
| *rbsK, PK, aroA* | MLSA5(145) | M60B177G14 | 0.6 | 0.09 | 0.03 | 0.008 | * | 0.002 | * |
| *rbsK* | MLSA5(145) | M65B141F14 | * | * | * | * | * | * | * |
| *rbsK* | MLSA5(145) | M60B504G12 | * | * | * | * | * | * | * |
| *rbsK* | MLSA5(145) | M60B609H12 | * | * | * | * | * | * | * |
| *rbsK* | MLSA5(145) | M60B580J12 | * | * | * | * | * | * | * |
| *rbsK* | MLSA5(145) | M60B715I02 | * | * | * | * | * | * | * |
| *rbsK* | MLSA5(145) | M60B730H18 | * | * | * | * | * | * | * |
| *rbsK* | MLSA5(145) | M65B053E10 | * | * | * | * | * | * | * |
| *rbsK* | MLSA5(145) | M65B155E13 | * | * | * | * | * | * | * |
| *rbsK* | MLSA5(145) | M60B071F22 | * | * | * | * | * | * | * |
| *rbsK* | MLSA5(145) | M60B207O10 | * | * | * | * | * | * | * |
| *rbsK* | MLSA5(145) | M60B349M23 | * | * | * | * | * | * | * |
| *rbsK* | MLSA5(145) | M60B547N16 | * | * | * | * | * | * | * |
| *rbsK* | MLSA5(145) | M60B691E09 | * | * | * | * | * | * | * |
| *rbsK* | MLSA5(145) | M65B038L09 | * | * | * | * | * | * | * |
| *rbsK* | MLSA5(145) | M65B063K13 | * | * | * | * | * | * | * |
| *rbsK* | MLSA5(145) | M65B080L10 | * | * | * | * | * | * | * |
| *rbsK* | MLSA5(145) | M65B102K09 | * | * | * | * | * | * | * |
| *rbsK* | MLSA5(145) | M65B078P18 | * | * | * | * | * | * | * |
| *rbsK, PK, CHP, aroA* | MLSA5(145) | M60B626D13 | 0.47 | 0.69 | >1.0 | >1.0 | * | 0.002 | * |
| *lepB, CHP, aroA* | MLSA5(145) | M65B150M20 | * | * | * | * | * | * | * |
| *rbsK* | MLSA5(145) | M65B150M20 | * | 0.27 | 0.004 | * | * | * | * |
| *lepB, CHP* | MLSA5(145) | M60B573N02 | * | * | * | * | * | * | * |
| *rbsK* | MLSA5(145) | M60B573N02 | * | * | * | * | * | * | * |
| *rbsK* | MLSA5(49) | M65B149H05 | * | 0.02 | 0.009 | * | * | * | * |
| *rbsK* | MLSA5(49) | M65B093I20 | * | 0.02 | 0.009 | * | * | * | * |
| *rbsK* | MLSA5(49) | M65B134I01 | * | 0.02 | 0.009 | * | * | * | * |
| *rbsK* | MLSA5(49) | M65B145I07 | * | 0.02 | 0.009 | * | * | * | * |
| *rbsK* | MLSA5(49) | M65B155E13 | * | 0.02 | 0.009 | * | * | * | * |
| *rbsK* | MLSA5(49) | M65B150M20 | * | 0.02 | 0.009 | * | * | * | * |
| *rbsK* | MLSA5(49) | M60B065P14 | * | * | * | * | * | * | * |
| *rbsK* | MLSA5(49) | M60B166J20 | * | * | * | * | * | * | * |
| *rbsK* | MLSA5(49) | M60B183F17 | * | * | * | * | * | * | * |
| *rbsK* | MLSA5(49) | M60B373K19 | * | * | * | * | * | * | * |
| *rbsK* | MLSA5(49) | M60B374C09 | * | * | * | * | * | * | * |
| *rbsK* | MLSA5(49) | M60B405K13 | * | * | * | * | * | * | * |
| *rbsK* | MLSA5(49) | M60B436G19 | * | * | * | * | * | * | * |
| *rbsK* | MLSA5(49) | M60B443F23 | * | * | * | * | * | * | * |
| *rbsK* | MLSA5(49) | M60B468P07 | * | * | * | * | * | * | * |
| *rbsK* | MLSA5(49) | M60B491M01 | * | * | * | * | * | * | * |
| *rbsK* | MLSA5(49) | M60B501M02 | * | * | * | * | * | * | * |
| *rbsK* | MLSA5(49) | M60B514A21 | * | * | * | * | * | * | * |
| *rbsK* | MLSA5(49) | M60B527I20 | * | * | * | * | * | * | * |
| *rbsK* | MLSA5(49) | M60B559P01 | * | * | * | * | * | * | * |
| *rbsK* | MLSA5(49) | M60B560H17 | * | * | * | * | * | * | * |
| *rbsK* | MLSA5(49) | M60B563B21 | * | * | * | * | * | * | * |
| *rbsK* | MLSA5(49) | M60B574L23 | * | * | * | * | * | * | * |
| *rbsK* | MLSA5(49) | M60B651G15 | * | * | * | * | * | * | * |
| *rbsK* | MLSA5(49) | M60B653P08 | * | * | * | * | * | * | * |
| *rbsK* | MLSA5(49) | M60B689K07 | * | * | * | * | * | * | * |
| *rbsK* | MLSA5(49) | M60B773C01 | * | * | * | * | * | * | * |
| *rbsK* | MLSA5(49) | M60B788A07 | * | * | * | * | * | * | * |
| *rbsK* | MLSA5(49) | M65B009D20 | * | * | * | * | * | * | * |
| *rbsK* | MLSA5(49) | M65B010I14 | * | * | * | * | * | * | * |
| *rbsK* | MLSA5(49) | M65B025J23 | * | * | * | * | * | * | * |
| *rbsK* | MLSA5(49) | M65B032B16 | * | * | * | * | * | * | * |
| *rbsK* | MLSA5(49) | M65B035K07 | * | * | * | * | * | * | * |
| *rbsK* | MLSA5(49) | M65B090N22 | * | * | * | * | * | * | * |
| *rbsK* | MLSA5(49) | M65B107B14 | * | * | * | * | * | * | * |
| *rbsK* | MLSA5(49) | M65B111J13 | * | * | * | * | * | * | * |
| *rbsK* | MLSA5(49) | M65B115H01 | * | * | * | * | * | * | * |

Supplementary Table 12 Continued…

| **Locus** | **Alignment**  **Analysis** | **Recombinant BAC** | **MaxChi** | **GENECONV** | **RDP** | **Chimaera** | **Siscan** | **3Seq** | **LARD** |
| --- | --- | --- | --- | --- | --- | --- | --- | --- | --- |
| *rbsK* | MLSA5(49) | M65B115I21 | * | * | * | * | * | * | * |
| *lepB, PK* | MLSA5(49) | M60B609H12 | 0.002 | * | * | 0.002 | * | * | * |
| *lepB, PK* | MLSA5(49) | M60B595E04 | 0.002 | * | * | 0.002 | * | * | * |
| *CHP, lepB* | MLSA5(49) | M60B653P08 c | * | * | * | * | * | * | * |
| *rbsK* | MLSA7 | M60B504G12 | * | 0.008 | * | * | * | * | * |
| *rbsK* | MLSA7 | M60B609H12 | * | 0.008 | * | * | * | * | * |
| *rbsK* | MLSA7 | M60B715I02 | * | 0.008 | * | * | * | * | * |
| *hisF, lepB, CHP, aroA* | MLSA7 | M60B740F14 | 0.03 | 0.01 | 0.005 | 0.02 | * | * | * |
| *CHP, aroA* | MLSA7 | M60B755A11c | * | * | * | * | 0.001 | * | * |
| *CHP, aroA* | MLSA7 | M60B725H17 | * | * | * | * | 0.001 | * | * |
| *hisF, lepB* | MLSA7 | M60B177G14 | 0.006 | * | * | 0.045 | * | * | * |
| *CHP, aroA* | MLSA7 | M60B177G14 | * | * | * | * | 0.001 | * | * |
| *CHP, aroA* | MLSA7 | M60B503C09 | * | * | * | * | 0.001 | * | * |
| *CHP, aroA* | MLSA7 | M60B478F03 | * | * | * | * | 0.001 | * | * |
| *CHP, aroA* | MLSA7 | M60B443F23 | * | * | * | * | 0.001 | * | * |
| *CHP, aroA* | MLSA7 | M60B463L24 | * | * | * | * | 0.001 | * | * |
| *CHP, aroA* | MLSA7 | M60B541E20 | * | * | * | * | 0.001 | * | * |
| *CHP, aroA* | MLSA7 | M60B563B21 | * | * | * | * | 0.001 | * | * |
| *CHP, aroA* | MLSA7 | M60B195D22 | * | * | * | * | 0.001 | * | * |
| *CHP, aroA* | MLSA7 | M60B653P08 | * | * | * | * | 0.001 | * | * |
| *CHP, aroA* | MLSA7 | M60B733O24 | * | * | * | * | 0.001 | * | * |
| *CHP, aroA* | MLSA7 | M60B399F15 | * | * | * | * | 0.001 | * | * |
| *CHP, aroA* | MLSA7 | M60B461H19 | * | * | * | * | 0.001 | * | * |
| *CHP, aroA* | MLSA7 | M60B247G10 | * | * | * | * | 0.001 | * | * |
| *CHP, aroA* | MLSA7 | M60B499M16 | * | * | * | * | 0.001 | * | * |
| *CHP, aroA* | MLSA7 | M60B436G19 | * | * | * | * | 0.001 | * | * |
| *rbsK-aroA* | MLSA7 | M60B589H13 | 0.005 | >1.0 | >1.0 | 0.049 | >1.0 | 0.001 | * |

*P-value < 0.001

^++^Genome annotation available at: <http://cmr.jcvi.org/cgibin/CMR/GenomePage.cgi?org=gymba>

## Supplementary Table 13. P-values for RDP4 analysis of recombinants for B´-like *Synechococcus* BACs^++^. Corresponds to Supplementary Table 11.

| **Locus** | **Alignment**  **Analysis** | **Recombinant**  **BAC** | **MaxChi** | **GENECONV** | **Bootscan** | **Chimaera** | **Siscan** | **3Seq** | **LARD** |
| --- | --- | --- | --- | --- | --- | --- | --- | --- | --- |
| *rbsK* | MLSA4 | M60B579B21 | * | * | * | * | * | * | * |
| *rbsK* | MLSA4 | M60B085O17 | * | * | * | * | * | * | * |
| *rbsK* | MLSA4 | M60B057N07 | * | * | * | * | * | * | * |
| *rbsK* | MLSA4 | M60B626O23 | * | * | * | * | * | * | * |
| *rbsK* | MLSA4 | M60B714K10 | * | * | * | * | * | * | * |
| *rbsK* | MLSA4 | M60B700J15 | * | * | * | * | * | * | * |
| *rbsK* | MLSA4 | M60B499C24 | * | * | * | * | * | * | * |
| *rbsK* | MLSA4 | M60B129N02 | * | * | * | * | * | * | * |
| *rbsK* | MLSA4 | M60B426N01 | * | * | * | * | * | * | * |
| *rbsK* | MLSA4 | M60B403F16 | * | * | * | * | * | * | * |
| *rbsK* | MLSA4 | M60B541D06 | * | * | * | * | * | * | * |
| *rbsK* | MLSA4 | M60B769K22 | * | * | * | * | * | * | * |
| *rbsK* | MLSA4 | M60B081O16 | * | * | * | * | * | * | * |
| *rbsK* | MLSA4 | M60B046B18 | * | * | * | * | * | * | * |
| *rbsK* | MLSA4 | M60B091H06 | * | * | * | * | * | * | * |
| *rbsK* | MLSA4 | M60B648C08 | * | * | * | * | * | * | * |
| *rbsK* | MLSA4 | M60B513G21 | * | * | * | * | * | * | * |
| *rbsK* | MLSA4 | M60B186K12 | * | * | * | * | * | * | * |
| *rbsK* | MLSA4 | M60B456A21 | * | * | * | * | * | * | * |
| *rbsK* | MLSA4 | M60B477G06 | * | * | * | * | * | * | * |
| *rbsK* | MLSA4 | M60B397N23 | * | * | * | * | * | * | * |
| *rbsK* | MLSA4 | M60B347P16 | * | * | * | * | * | * | * |
| *rbsK* | MLSA4 | M60B250B15 | * | * | * | * | * | * | * |
| *rbsK/pcrA* | MLSA4 | M60B115G12 | 0.004 | 0.004 | 0.002 | >1.0 | >1.0 | 0.2 | * |
| *16SrRNA/ITS* | MLSA4 | M60B420G12 | >1.0 | * | * | >1.0 | 0.01 | * | * |
| *16SrRNA/ITS* | MLSA4 | M60B397N23 | >1.0 | * | * | >1.0 | 0.01 | * | * |
| *16SrRNA/ITS* | MLSA4 | M60B400M20 | >1.0 | * | * | >1.0 | 0.01 | * | * |

*P-value < 0.001

^++^Genome annotation available at: [http://cmr.jcvi.org/cgibin/CMR/GenomePage .cgi?org=gymb](http://cmr.jcvi.org/cgi-bin/CMR/GenomePage.cgi?org=gymb)

## Supplementary Table 14. Sequence datasets used to infer between A- and B´-like *Synechococcus* BAC recombinants (R) using RDP4.^a^

| **Gene** | **Dataset** | **No. Sequences** | **Alignment Length** | **Recombination Events** | **Clones/STs involved** | **Evidence, p value (p)^c^** | **Notes** |
| --- | --- | --- | --- | --- | --- | --- | --- |
| 16S rRNA | *Synechococcus* A/ B´-like combined – 60^o^C and 65^o^C | 161 | 505 | 0 | 0 | n/a |  |
| *rbsK* | *Synechococcus* A/ B´-like combined – 60^o^C and 65^o^C | 123 | 511 | 3^b^ | 1. SynBM60BST13 (R), SynAM65BST36 (parent) 2. SynBM60BST42 (R) ***or*** SynBM60BST45 (R), SynAM60BST56 (parent) 3. SynBM60BST3 (R), SynAM60BST63 (parent) | 1. All methods p<.001 2. All methods p<.001 3. GENECONV .026, Bootscan .002, MaxChi/LARD/ Siscan <.001 | 1. No beginning breakpoint identified 2. No beginning breakpoint identified 3. No beginning breakpoint identified |

^a^ Dataset was combined A/B´ sequences ‘normalized’ meaning the same number of sequences and same lengths of nucleotides analyzed.

^b^ Total recombination event detecteds was 8, but only unique recombination events between A- and B´-like BAC clones are noted in table.

^c^ RDP4 methods: RDP, GENECONV, MaxChi, Chimera, Bootscan, Siscan, 3Seq and LARD ([5](#_ENREF_5))

## Supplementary Table 15. Linkage disequilibrium and per site rho and theta results from analysis of concatenated sequence data sets using LIAN, LDHat and Clonal Frame analysis.

|  | | | | | | | | | | | **LDHat^m^** | | **Clonal Frame^n^** | |
| --- | --- | --- | --- | --- | --- | --- | --- | --- | --- | --- | --- | --- | --- | --- |
| **Organism** | **Sample Size** | **No. of Loci** | **No. Nts** | **Mean Allelic Diversity^a^** | **V_D_^b^** | **V_e_^c^** | **L_MC_^d^** | **L_PARA_^e^** | **I_A_^S f^** | **Linkage Detected^g^** | **θ/site** | **R/site** | **θ/site** | **R/site** |
| *Synechococcus* A-like | 50 STs | 7^j^ | 4008 | 0.389 + 0.091 | 2.13 | 1.32 | 1.60 | 1.56 | 0.103 | Yes | 0.01 | 0.0004-0.01 | 0.008 | 0.003-0.008 |
| *Synechococcus* A-like^h^ | 27 STs | 5 ^k^ | 2711 | 0.63 + 0.073 | 2.32 | 1.07 | 1.26 | 1.24 | 0.30 | Yes | NC | NC | NC | NC |
| *Synechococcus* A-like^i^ | 69 STs | 5 ^k^ | 2760 | 0.54 + 0.084 | 2.17 | 1.10 | 1.22 | 1.23 | 0.242 | Yes | 0.017 | 0.001-0.016 | 0.015 | 0.006-0.013 |
| *Synechococcus* B´-like | 51 STs | 4 ^l^ | 2448 | 0.859 + 0.022 | 0.98 | 0.48 | 0.51 | 0.51 | 0.353 | Yes | 0.033 | 0-0.034 | 0.026 | 0.006-0.014 |

NC: not calculated

^a^ The number of base substitutions per site from mean diversity calculations for the entire population are shown (see eq. 12.73 in ref. ([6](#_ENREF_6))). Analyses were conducted using the Maximum Composite Likelihood model ([7](#_ENREF_7)). Codon positions included were 1st+2nd+3rd+Noncoding. All positions containing gaps and missing data were eliminated. Evolutionary analyses were conducted in MEGA4 ([8](#_ENREF_8)).

^b^ V_D_: The observed mismatch variance.

^c^ V_e_: The expected mismatch variance at equilibrium.

^d^ L_MC_: Simulated 5% critical value.

^e^ L_PARA_: Calculated 5% critical value assuming a normal distribution:

L = V_e_ + 1.654√Var(V_D_) ([9](#_ENREF_9)).

^f^ I_A_^S^: Standardized index of association.

^g^ Detected at a p-value < 0.01

^h^ Based on analysis of 49 A-like BACs from 60^o^C and 65^o^C samples.

^i^ Based on analysis of 145 A-like BACs from 60^o^C and 65^o^C samples.

^j^  *rbsK, PK, lepB, aroA, CHP, hisF, dnaG*

^k^ *rbsK, PK, lepB, aroA, CHP*

^l^ *pcrA, rbsK, aroA*, 16S rRNA

^m^ Calculations based on main-text Tables 1 and 2. Range of ‘R’ taken from range across methods using LDHat analysis.

^n^ Calculations based on main-text Tables 1 and 2. Range of ‘R’ taken from range across of ‘R’ confidence interval (CI) for Clonal Frame amalysis.

## Supplementary Table 16. Ecotype Simulation and eBURST output for B´-like *Synechococcus* BACs.

|  |  | **Ecotype Simulation (ES)** | | | | **eBURST^d^** | |
| --- | --- | --- | --- | --- | --- | --- | --- |
| **Locus** | **Estimated Evolutionary Divergence (BACs)^a^** | **PEs Demarcated-ES (95% CI)** | **Omega**  **(95% CI)** | **Sigma**  **(95% CI)** | **Sample- Specific PEs** | **No. of Alleles or Sequence Types** | **No. of Clonal Complexes** |
| *aroA* | 0.023 | 9 (5-46) | 0.17 (0.04-0.52) | 15 (0.7-100) | ^c^ | 17 | ^d^ |
| *rbsK* | 0.066 | 22 (11-36) | 0.08 (0.04-0.11) | 93.5 (0.6-100) | ^c^ | 21 | ^d^ |
| *pcrA* | 0.014 | 13 (7-30) | 0.165 (0.051-0.37) | 102 (1.02->100) | ^c^ | 17 | ^d^ |
| 16S rRNA /ITS | 0.005 | 8 (6-47) | 0.28 (0.12-0.39) | 66 (0.42-100) | ^c^ | 29 | ^d^ |
| Concatenation  with 16S/ITS^b^ | 0.024 | 29 (11-68) | 0.051 (0.034-0.08) | 0.66 (0.42-100) | ^c^ | 51 | 3 |
| Concatenation  Without 16S/ITS**^b^** | 0.036 | 25 (22-43) | 0.05 (0.04-0.08) | 24.4 (2.4-100) | ^c^ | 33 | 1 |

^a^ The number of base substitutions per site from averaging over all sequence pairs are shown. Analyses were conducted using the Maximum Composite Likelihood model ([6](#_ENREF_6)). Codon positions included were 1st+2nd+3rd+Noncoding. All positions containing gaps and missing data were eliminated. Evolutionary analyses were conducted in MEGA4 ([8](#_ENREF_8)).

^b^ Calculated from concatenated sequence datasets only.

^c^ *Synechococcus* B´-like BACs were only retrieved from one temperature site, 60^o^C.

^d^ Clonal complexes only apply to concatenated sequence datasets.

## Supplementary Table 17. Comparison of singleton STs surrounding dominant variants of PEs and consensus sequences of clonal complexes observed in 4-locus MLSA of B´-like *Synechococcus* BACs. STs common to a PE and a corresponding clonal complex are highlighted in grey.

| **B´-Like *Synechococcus* 4 Locus MLSA** | | | | | | | |
| --- | --- | --- | --- | --- | --- | --- | --- |
| **PE** | **DV-**  **ST** | **No. of Sequences of DV/sDV-ST** | **Variant STs within PE** | **Clonal Complex** | **Consensus ST** | **No. of Sequences with consensus ST** | **STs (SLVs) within a Clonal complex** |
| 1 | 1 | 11 | 7, 8, 12 | B4-I | 1 | 11 | 7, 8, 9, 10, 11, 12 |
| 2 | ^a^ | ^a^ | 9, 10, 11 | ^d^ | ^d^ | ^d^ | ^d^ |
| 5 | ^a^ | ^a^ | 37, 38 | ^d^ | ^d^ | ^d^ | ^d^ |
| 6 | 6 | 2 | 25, 26, 27, 28, 29 | B4-II | 6 | 2 | 24, 25, 26, 27, 28, 29 |
| 13 | ^a^ | ^a^ | 39, 40 | ^d^ | ^d^ | ^d^ | ^d^ |
| 16 | 5 | 2 | 31, 32, 33, 34 | B4-III | 5 | 2 | 30, 31, 32, 33, 34 |
| 18 | 4 | 3 | 21, 22 | ^b^ | 4 | 3 | 21, 22 |
| 22 | 2 | 6 | 17 | ^d^ | ^d^ | ^d^ | ^d^ |
| 25 | ^a^ | ^a^ | 19, 35, 36, 51 | ^b^ | 35 | 1 | 36, 51 |
| 27 | 3 | 4 | ^c^ | ^d^ | ^d^ | ^d^ | ^d^ |

**DV: dominant variant**

**sDV:** subdominant variant.

**SLV:** single locus variant

^a^ PE did not contain a dominant variant.

^b^ PE ST variants appear within a sub-clonal complex (relaxed criterion of 2 single-locus variants).

^c^ PE did not contain any other variants.

^d^ PE ST variants do not appear within a clonal complex.

## Supplementary Table 18. eBURST analysis of clonal complexes for the 4-locus MLSA of B´-like *Synechococcus* BACs. The number of BACs within STs is in parentheses when greater than 1. Superscripts next to the allele number denote number of nucleotide differences compared to the consensus sequence.

| **Clonal Complex B4-I** | | | | | | | | | | | |
| --- | --- | --- | --- | --- | --- | --- | --- | --- | --- | --- | --- |
| Locus | Consensus ST1 (11) | ST7 | ST8 | | ST9 | | ST10 | | ST11 | | ST12 |
| *rbsK* | 1 | 1 | 1 | | 1 | | 1 | | 1 | | 16^5^ |
| *pcrA* | 1 | 1 | 1 | | 1 | | 1 | | 1 | | 1 |
| *aroA* | 1 | 1 | 1 | | 1 | | 1 | | 1 | | 1 |
| 16S rRNA/ITS | 1 | 5^1^ | 13^5^ | | 17^24^ | | 20^13^ | | 26^5^ | | 1 |
| **Clonal Complex B4-II** | | | | | | | | | | | |
| Locus | Consensus ST6 (2) | ST24 | ST25 | | ST26 | | ST27 | | ST28 | | ST29 |
| *rbsK* | 5 | 5 | 5 | | 5 | | 5 | | 14^1^ | | 5 |
| *pcrA* | 2 | 2 | 2 | | 2 | | 2 | | 2 | | 13^2^ |
| *aroA* | 3 | 3 | 3 | | 3 | | 3 | | 3 | | 3 |
| 16S rRNA/ITS | 3 | 8^14^ | 10^4^ | | 15^5^ | | 28^3^ | | 3 | | 3 |
| **Clonal Complex B4-III** | | | | | | | | | | | |
| Locus | Consensus ST5 (2) | ST30 | | ST31 | | ST32 | | ST33 | | ST34 | |
| *rbsK* | 4 | 3^14^ | | 4 | | 4 | | 4 | | 4 | |
| *pcrA* | 5 | 5 | | 5 | | 5 | | 5 | | 5 | |
| *aroA* | 4 | 4 | | 4 | | 4 | | 4 | | 4 | |
| 16S rRNA/ITS | 4 | 4 | | 1^1^ | | 6^15^ | | 7^1^ | | 14^9^ | |

## Supplementary Table 19. Allelic profiles generated from analysis of single nucleotide polymorphisms in 49 A-like *Synechococcus* BACs for protein-encoding datasets of 5 loci.

| **Clone^a^** | **ST^b^** | ***aroA*** | ***CHP*** | ***lepB*** | ***PK*** | ***rbsK*** |
| --- | --- | --- | --- | --- | --- | --- |
| M60B166J20 | 1 | 1 | 1 | 1 | 1 | 1 |
| M60B374C09 | 1 | 1 | 1 | 1 | 1 | 1 |
| M60B405K13 | 1 | 1 | 1 | 1 | 1 | 1 |
| M60B501M02 | 1 | 1 | 1 | 1 | 1 | 1 |
| M60B514A21 | 1 | 1 | 1 | 1 | 1 | 1 |
| M60B559P01 | 1 | 1 | 1 | 1 | 1 | 1 |
| M60B574L23 | 1 | 1 | 1 | 1 | 1 | 1 |
| M60B651G15 | 1 | 1 | 1 | 1 | 1 | 1 |
| M60B689K07 | 1 | 1 | 1 | 1 | 1 | 1 |
| M60B773C01 | 1 | 1 | 1 | 1 | 1 | 1 |
| M60B788A07 | 1 | 1 | 1 | 1 | 1 | 1 |
| M65B010I14 | 1 | 1 | 1 | 1 | 1 | 1 |
| M65B107B14 | 1 | 1 | 1 | 1 | 1 | 1 |
| M65B093I20 | 2 | 3 | 3 | 3 | 4 | 6 |
| M65B134I01 | 2 | 3 | 3 | 3 | 4 | 6 |
| M65B145I07 | 2 | 3 | 3 | 3 | 4 | 6 |
| M65B149H05 | 2 | 3 | 3 | 3 | 4 | 6 |
| M65B155E13 | 2 | 3 | 3 | 3 | 4 | 6 |
| M60B183F17 | 3 | 1 | 1 | 1 | 2 | 5 |
| M60B527I20 | 3 | 1 | 1 | 1 | 2 | 5 |
| M65B009D20 | 3 | 1 | 1 | 1 | 2 | 5 |
| M65B025J23 | 3 | 1 | 1 | 1 | 2 | 5 |
| M60B065P14 | 4 | 1 | 1 | 2 | 1 | 1 |
| M60B560H17 | 4 | 1 | 1 | 2 | 1 | 1 |
| M60B443F23 | 5 | 1 | 2 | 1 | 1 | 2 |
| M60B563B21 | 5 | 1 | 2 | 1 | 1 | 2 |
| M60B468P07 | 6 | 1 | 1 | 1 | 1 | 8 |
| M60B491M01 | 6 | 1 | 1 | 1 | 1 | 8 |
| M65B141F14 | 7 | 1 | 1 | 1 | 2 | 3 |
| M60B373K19 | 8 | 1 | 1 | 1 | 1 | 10 |
| M65B078P18 | 9 | 1 | 1 | 1 | 8 | 12 |
| M65B053E10 | 10 | 1 | 1 | 2 | 6 | 7 |
| M65B151I23 | 11 | 1 | 1 | 3 | 2 | 5 |
| M60B609H12 | 12 | 1 | 1 | 5 | 4 | 7 |
| M60B595E04 | 13 | 1 | 1 | 5 | 5 | 17 |
| M60B653P08 | 14 | 1 | 2 | 1 | 1 | 9 |
| M65B035K07 | 15 | 1 | 2 | 1 | 2 | 14 |
| M65B032B16 | 16 | 1 | 4 | 1 | 1 | 2 |
| M65B150M20 | 17 | 1 | 4 | 3 | 4 | 7 |
| M65B111J13 | 18 | 1 | 6 | 4 | 1 | 4 |
| M65B080L10 | 19 | 2 | 1 | 1 | 3 | 3 |
| M65B090N22 | 20 | 2 | 1 | 1 | 3 | 16 |

Supplementary Table 19 Continued…

| **Clone^a^** | **ST^b^** | | ***rbsK*** | ***pk*** | ***lepB*** | ***chp*** | ***aroA*** |
| --- | --- | --- | --- | --- | --- | --- | --- |
| M65B115H01 | | 21 | 2 | 4 | 1 | 3 | 4 |
| M65B115I21 | | 22 | 3 | 1 | 1 | 1 | 4 |
| M65B104K09 | | 23 | 3 | 1 | 4 | 1 | 11 |
| M65B063K13 | | 24 | 3 | 1 | 4 | 5 | 13 |
| *Synechococcus* strain A | | 25 | 3 | 2 | 2 | 7 | 3 |
| M60B436G19 | | 26 | 4 | 2 | 4 | 2 | 4 |
| M65B019P07 | | 27 | 5 | 5 | 2 | 1 | 15 |

^a^ Clone names were compiled from spring (M = Mushroom), temperature (60 or 65), BAC library plate (1-792 for 60^o^C; 1-168 for 65^o^C) and specific well of that plate (A-P and 1-24). Example: M65134I01, Mushroom Spring, 65°C library, plate #134 well I-01.

^b^ ST = sequence type.

## Supplementary Table 20. Allelic profiles generated from analysis of single nucleotide polymorphisms in 145 A-like *Synechococcus* BACs for protein-encoding datasets of 5 loci.

| **Clone^a^** | **ST^b^** | ***rbsK*** | ***pk*** | ***lepB*** | ***CHP*** | ***aroA*** |
| --- | --- | --- | --- | --- | --- | --- |
| M60B102F16 | 1 | 1 | 1 | 1 | 1 | 1 |
| M60B120F08 | 1 | 1 | 1 | 1 | 1 | 1 |
| M60B130N03 | 1 | 1 | 1 | 1 | 1 | 1 |
| M60B166J20 | 1 | 1 | 1 | 1 | 1 | 1 |
| M60B180B19 | 2 | 2 | 1 | 1 | 1 | 1 |
| M60B183F17 | 2 | 2 | 1 | 1 | 1 | 1 |
| M60B187L01 | 1 | 1 | 1 | 1 | 1 | 1 |
| M60B360H08 | 1 | 1 | 1 | 1 | 1 | 1 |
| M60B374C09 | 1 | 1 | 1 | 1 | 1 | 1 |
| M60B384M22 | 1 | 1 | 1 | 1 | 1 | 1 |
| M60B390N18 | 1 | 1 | 1 | 1 | 1 | 1 |
| M60B405K13 | 1 | 1 | 1 | 1 | 1 | 1 |
| M60B413O21 | 1 | 1 | 1 | 1 | 1 | 1 |
| M60B414C18 | 1 | 1 | 1 | 1 | 1 | 1 |
| M60B457N03 | 1 | 1 | 1 | 1 | 1 | 1 |
| M60B471D04 | 1 | 1 | 1 | 1 | 1 | 1 |
| M60B494K10 | 1 | 1 | 1 | 1 | 1 | 1 |
| M60B501M02 | 1 | 1 | 1 | 1 | 1 | 1 |
| M60B502F22 | 1 | 1 | 1 | 1 | 1 | 1 |
| M60B514A21 | 1 | 1 | 1 | 1 | 1 | 1 |
| M60B515B22 | 1 | 1 | 1 | 1 | 1 | 1 |
| M60B516C08 | 1 | 1 | 1 | 1 | 1 | 1 |
| M60B559P01 | 1 | 1 | 1 | 1 | 1 | 1 |
| M60B574L23 | 1 | 1 | 1 | 1 | 1 | 1 |
| M60B588E12 | 1 | 1 | 1 | 1 | 1 | 1 |
| M60B589H13 | 1 | 1 | 1 | 1 | 1 | 1 |
| M60B651G15 | 1 | 1 | 1 | 1 | 1 | 1 |
| M60B658F14 | 1 | 1 | 1 | 1 | 1 | 1 |
| M60B663O15 | 1 | 1 | 1 | 1 | 1 | 1 |
| M60B681M12 | 1 | 1 | 1 | 1 | 1 | 1 |
| M60B689K07 | 1 | 1 | 1 | 1 | 1 | 1 |
| M60B701L08 | 1 | 1 | 1 | 1 | 1 | 1 |
| M60B720O05 | 1 | 1 | 1 | 1 | 1 | 1 |
| M60B729E01 | 1 | 1 | 1 | 1 | 1 | 1 |
| M60B773C01 | 1 | 1 | 1 | 1 | 1 | 1 |
| M60B788A07 | 1 | 1 | 1 | 1 | 1 | 1 |
| M65B010I14 | 1 | 1 | 1 | 1 | 1 | 1 |
| M65B107B14 | 1 | 1 | 1 | 1 | 1 | 1 |

Supplementary Table 20 Continued…

| **Clone^a^** | **ST^b^** | ***rbsK*** | ***pk*** | ***lepB*** | ***CHP*** | ***aroA*** |
| --- | --- | --- | --- | --- | --- | --- |
| M60B003C20 | 6 | 1 | 1 | 1 | 2 | 1 |
| M60B065P14 | 5 | 1 | 1 | 2 | 1 | 1 |
| M60B071F22 | 10 | 10 | 1 | 1 | 1 | 1 |
| M60B116E07 | 5 | 1 | 1 | 2 | 1 | 1 |
| M60B144H03 | 5 | 1 | 1 | 2 | 1 | 1 |
| M60B205B12 | 2 | 2 | 1 | 1 | 1 | 1 |
| M60B207O10 | 9 | 8 | 5 | 3 | 1 | 4 |
| M60B349M23 | 9 | 8 | 5 | 3 | 1 | 4 |
| M60B389C22 | 2 | 2 | 1 | 1 | 1 | 1 |
| M60B393F20 | 10 | 10 | 1 | 1 | 1 | 1 |
| M60B399F15 | 3 | 3 | 1 | 1 | 2 | 1 |
| M60B405I18 | 7 | 2 | 4 | 1 | 1 | 1 |
| M60B419M11 | 8 | 6 | 1 | 1 | 1 | 1 |
| M60B441D14 | 2 | 2 | 1 | 1 | 1 | 1 |
| M60B443F23 | 3 | 3 | 1 | 1 | 2 | 1 |
| M60B461H19 | 3 | 3 | 1 | 1 | 2 | 1 |
| M60B468P07 | 11 | 12 | 1 | 1 | 1 | 1 |
| M60B491M01 | 11 | 12 | 1 | 1 | 1 | 1 |
| M60B527I20 | 2 | 2 | 1 | 1 | 1 | 1 |
| M60B533H13 | 5 | 1 | 1 | 2 | 1 | 1 |
| M60B533K05 | 7 | 2 | 4 | 1 | 1 | 1 |
| M60B541E20 | 6 | 1 | 1 | 1 | 2 | 1 |
| M60B563B21 | 3 | 3 | 1 | 1 | 2 | 1 |
| M60B582M12 | 3 | 3 | 1 | 1 | 2 | 1 |
| M60B589J13 | 2 | 2 | 1 | 1 | 1 | 1 |
| M60B616K18 | 13 | 2 | 9 | 1 | 1 | 1 |
| M60B634E12 | 8 | 6 | 1 | 1 | 1 | 1 |
| M60B645L03 | 3 | 3 | 1 | 1 | 2 | 1 |
| M60B649P18 | 8 | 6 | 1 | 1 | 1 | 1 |
| M60B690I15 | 8 | 6 | 1 | 1 | 1 | 1 |
| M60B696K05 | 2 | 2 | 1 | 1 | 1 | 1 |
| M60B740F14 | 12 | 1 | 1 | 7 | 1 | 1 |
| M60B755A11 | 6 | 1 | 1 | 1 | 2 | 1 |
| M60B760D02 | 2 | 2 | 1 | 1 | 1 | 1 |
| M60B761P11 | 12 | 1 | 1 | 7 | 1 | 1 |
| M60B788B16 | 7 | 2 | 4 | 1 | 1 | 1 |
| M65B009D20 | 2 | 2 | 1 | 1 | 1 | 1 |
| M65B025J23 | 2 | 2 | 1 | 1 | 1 | 1 |
| M65B093I20 | 4 | 7 | 3 | 4 | 4 | 3 |
| M65B134I01 | 4 | 7 | 3 | 4 | 4 | 3 |
| M65B145I07 | 4 | 7 | 3 | 4 | 4 | 3 |
| M65B149H05 | 4 | 7 | 3 | 4 | 4 | 3 |
| M65B155E13 | 4 | 7 | 3 | 4 | 4 | 3 |

Supplementary Table 20 Continued…

| **Clone^a^** | **ST^b^** | ***rbsK*** | ***pk*** | ***lepB*** | ***CHP*** | ***aroA*** |
| --- | --- | --- | --- | --- | --- | --- |
| M60B030P24 | 32 | 2 | 6 | 1 | 1 | 1 |
| M60B037J04 | 22 | 1 | 6 | 2 | 1 | 1 |
| M60B051K07 | 16 | 4 | 2 | 1 | 3 | 2 |
| M60B066K08 | 28 | 2 | 2 | 1 | 1 | 1 |
| M60B083K10 | 29 | 2 | 4 | 1 | 1 | 2 |
| M60B086E07 | 30 | 2 | 4 | 1 | 6 | 5 |
| M60B092K15 | 42 | 4 | 2 | 2 | 2 | 2 |
| M60B168G06 | 15 | 4 | 2 | 1 | 2 | 2 |
| M60B177G14 | 43 | 4 | 2 | 8 | 2 | 2 |
| M60B195D22 | 14 | 3 | 1 | 1 | 3 | 1 |
| M60B198G06 | 20 | 1 | 1 | 1 | 5 | 1 |
| M60B247G10 | 34 | 3 | 1 | 2 | 2 | 1 |
| M60B410O11 | 48 | 6 | 1 | 1 | 1 | 6 |
| M60B467J19 | 18 | 5 | 7 | 1 | 1 | 1 |
| M60B478F03 | 15 | 4 | 2 | 1 | 2 | 2 |
| M60B499M16 | 41 | 4 | 2 | 1 | 7 | 2 |
| M60B503C09 | 40 | 4 | 2 | 1 | 2 | 1 |
| M60B504G12 | 17 | 5 | 3 | 1 | 1 | 1 |
| M60B575O18 | 25 | 2 | 1 | 2 | 1 | 1 |
| M60B609H12 | 46 | 5 | 3 | 6 | 1 | 1 |
| M60B626D13 | 24 | 1 | 13 | 1 | 1 | 1 |
| M60B626L24 | 37 | 3 | 9 | 1 | 2 | 1 |
| M60B648A16 | 13 | 2 | 9 | 1 | 1 | 1 |
| M60B653P08 | 19 | 6 | 1 | 1 | 2 | 1 |
| M60B680J12 | 18 | 5 | 7 | 1 | 1 | 1 |
| M60B690F12 | 21 | 1 | 1 | 2 | 5 | 1 |
| M60B715I02 | 45 | 5 | 3 | 5 | 1 | 1 |
| M60B725H17 | 35 | 3 | 1 | 5 | 2 | 1 |
| M60B730H18 | 17 | 5 | 3 | 1 | 1 | 1 |
| M60B733O24 | 19 | 6 | 1 | 1 | 2 | 1 |
| M60B753F06 | 31 | 2 | 4 | 5 | 1 | 1 |
| M60B782C12 | 27 | 2 | 1 | 5 | 1 | 2 |
| M65B032B16 | 14 | 3 | 1 | 1 | 3 | 1 |
| M65B035K07 | 49 | 25 | 1 | 1 | 1 | 1 |
| M65B053E10 | 47 | 5 | 7 | 2 | 1 | 1 |
| M65B078P18 | 36 | 26 | 7 | 1 | 1 | 1 |
| M65B090N22 | 33 | 27 | 2 | 1 | 1 | 2 |
| M65B104K09 | 23 | 28 | 1 | 3 | 5 | 1 |
| M65B111J13 | 39 | 4 | 1 | 3 | 12 | 1 |
| M65B115H01 | 16 | 4 | 2 | 1 | 3 | 2 |
| M65B115I21 | 38 | 4 | 1 | 1 | 1 | 3 |
| M65B150M20 | 44 | 5 | 3 | 4 | 3 | 1 |
| M65B151I23 | 26 | 2 | 1 | 4 | 1 | 1 |

Supplementary Table 20 Continued…

| **Clone^a^** | **ST^b^** | ***rbsK*** | ***pk*** | ***lepB*** | ***CHP*** | ***aroA*** |
| --- | --- | --- | --- | --- | --- | --- |
| M60B373K19 | 58 | 13 | 1 | 1 | 1 | 1 |
| M60B379G20 | 56 | 11 | 5 | 1 | 1 | 1 |
| M60B436G19 | 59 | 14 | 1 | 3 | 2 | 7 |
| M60B463L24 | 60 | 15 | 1 | 1 | 2 | 1 |
| M60B534F12 | 61 | 16 | 11 | 1 | 1 | 1 |
| M60B535B15 | 62 | 17 | 6 | 1 | 1 | 1 |
| M60B547N16 | 63 | 18 | 5 | 8 | 1 | 4 |
| M60B553A15 | 64 | 19 | 1 | 1 | 1 | 1 |
| M60B573N02 | 65 | 20 | 6 | 1 | 8 | 8 |
| M60B591D09 | 66 | 21 | 12 | 1 | 1 | 1 |
| M60B595A23 | 67 | 22 | 1 | 3 | 1 | 2 |
| M60B595E04 | 57 | 11 | 5 | 6 | 1 | 1 |
| M60B605H09 | 68 | 23 | 3 | 1 | 2 | 1 |
| M60B691E09 | 51 | 8 | 14 | 3 | 9 | 3 |
| M65B019P07 | 69 | 24 | 1 | 2 | 10 | 5 |
| M65B038L09 | 53 | 9 | 1 | 1 | 11 | 1 |
| M65B063K13 | 50 | 8 | 5 | 3 | 1 | 2 |
| M65B080L10 | 54 | 9 | 2 | 1 | 1 | 2 |
| M65B141F14 | 52 | 9 | 1 | 1 | 1 | 1 |
| *Synechococcus* sp. A | 55 | 9 | 15 | 2 | 2 | 3 |

^a^ Clone names were compiled from spring (M = Mushroom), temperature (60 or 65), BAC library plate (1-792 for 60^o^C; 1-168 for 65^o^C) and specific well of that plate (A-P and 1-24). Example: M65134I01, Mushroom Spring, 65°C library, plate #134 well I-01.

^b^ ST = sequence type.

##

## Supplementary Table 21. Ecotype Simulation and eBURST output for A-like *Synechococcus* BACs, 5-locus study of 49 sequences.

| **Locus** | **Average Evolutionary Divergence (BACs)^a^** | **Ecotype Simulation (ES)** | | | | **eBURST^c^** | |
| --- | --- | --- | --- | --- | --- | --- | --- |
|  |  | **PEs Demarcated-ES (95% CI)** | **Omega**  **(95% CI)** | **Sigma**  **(95% CI)** | **Sample- Specific PEs** | **No. of Alleles or Sequence Types** | **No. of Clonal Complexes** |
| *rbsK* | 0.051 | 9 (5-20) | 0.072  (0.02-0.2) | 6.56  (0.02-100) | Yes | 17 | ^c^ |
| *PK* | 0.003 | 4 (3-27) | 0.628  0.19-2.98 | 64.2  1.92-100 | Yes | 8 | ^c^ |
| *lepB* | 0.004 | 2 (2-4) | 0.06  0.002-0.3 | 15.6  1.06-100 | No | 5 | ^c^ |
| *CHP* | 0.003 | 2 (2-7) | 0.097  0.006-0.3 | 13.3  (1.31-100) | No | 6 | ^c^ |
| *aroA* | 0.001 | 1 (1-49) | 9.2  (2e-7-100) | 8.7  (0.1->100) | No | 5 | ^c^ |
| Concatenation^b^ | 0.012 | 13 (8-39) | 0.071  3.6e-2-0.12 | 9.22  (1.87-100) | Yes | 27 | 1^d^ |

^a^ The number of base substitutions per site from averaging over all sequence pairs are shown. Analyses were conducted using the Maximum Composite Likelihood model ([6](#_ENREF_6)). Codon positions included were 1st+2nd+3rd+Noncoding. All positions containing gaps and missing data were eliminated. Evolutionary analyses were conducted in MEGA4 ([8](#_ENREF_8)).

^b^ Calculated from concatenated sequence datasets only.

^c^ Clonal complexes only apply to concatenated sequence datasets.

^d^ Three clonal complexes were visualized if the criterion was relaxed to allow 2 single-locus variants to define a clonal complex. See Supplementary Figure 17.

## Supplementary Table 22. eBURST analysis of clonal complexes for the 5-locus MLSA of A-like *Synechococcus* BACs (49 sequences). The number of BACs within an ST is in parentheses when greater than 1. Superscripts next to the allele number denote number of nucleotide differences compared to the consensus sequence. Corresponding to Supplementary Figure 17.

| **Clonal Complex A5-I** | | | | | |
| --- | --- | --- | --- | --- | --- |
| **Locus** | **Consensus ST1 (13)** | **ST4 (2)** | **ST6 (2)** | | **ST8** |
| *aroA* | 1 | 1 | 1 | | 1 |
| *CHP* | 1 | 1 | 1 | | 1 |
| *lepB* | 1 | 1 | 2^1^ | | 1 |
| *PK* | 1 | 1 | 1 | | 1 |
| *rbsK* | 1 | 8^1^ | 1 | | 10^3^ |
| **Sub-Clonal Complex A5-i** | | | | | |
| **Locus** | **Consensus ST5 (2)** | **ST14** | | **ST16** | |
| *aroA* | 1 | 1 | | 1 | |
| *CHP* | 2 | 4^1^ | | 2 | |
| *lepB* | 1 | 1 | | 1 | |
| *PK* | 1 | 1 | | 1 | |
| *rbsK* | 2 | 2 | | 9^9^ | |
| **Sub-Clonal Complex A5-ii** | | | | | |
| **Locus** | **Consensus ST3 (4)** | **ST11** | | **ST7** | |
| *aroA* | 1 | 1 | | 1 | |
| *CHP* | 1 | 1 | | 1 | |
| *lepB* | 1 | 3^1^ | | 1 | |
| *PK* | 2 | 2 | | 2 | |
| *rbsK* | 5 | 5 | | 3^47^ | |

## Supplementary Table 23. Comparison of singleton STs surrounding dominant variants of PEs and consensus sequences of clonal complexes observed in 7-locus MLSA of A-like *Synechococcus* BACs. STs common to a PE and a corresponding clonal complex are highlighted in bold.

| **A-Like *Synechococcus* 7 Locus MLSA^e^ (no. of BACs in analysis = 71)** | | | | | | | |
| --- | --- | --- | --- | --- | --- | --- | --- |
| **PE** | **DV-ST** | **No. of Sequences of DV/sDV-ST** | **Variant STs within PE** | **Clonal Complex** | **Consensus ST** | **No. of Sequences with consensus ST** | **STs (SLVs) within a Clonal complex** |
| 1 | 4 | 2 | ^c^ | A7-I | 1 | 14 | 2, 3, 4, 6, 8, 11, 12, 14, 15, 33, 36, 39, 41, 43, 48, 49 |
| 3 | ^a^ | ^a^ | 28.29.30.44 | ^d^ | ^d^ | ^d^ | ^d^ |
| 4 | 5^b^ | 2 | 7, **11,** 16, **18, 19, 21, 31,**  **45, 46** | A7-II | 5 | 2 | **11, 18, 19, 21, 31, 45,** **46** |
| 4 | 7^b^ | 2 | 5, 11, 16, 18, 19, 21, 31,  45, 46 | A7-III | 6 | 2 | 1, 7, 22, 23 |
| 5 | 9 | 2 | 17, 20, 26, 32 | ^d^ | ^d^ | ^d^ | ^d^ |
| 6 | 8 | 2 | **36,** 37,38, **39**, **48,** 50 | A7-I | 1 | 14 | 2, 3, 4, 6, 8, 11, 12, 14, 15, 33, **36,** **39**, 41, 43, **48,** 49 |
| 7 | 1 | 14 | **2, 3, 6, 14, 15,** 22, 23, **33,** **41, 43, 49** | A7-I | 1 | 14 | **2, 3,** 4, **6,** 8, 11, 12, **14, 15,** **33,** 36, 39, **41, 43,** 48, **49** |
| 7 | 2^b^ | 2 | 1**, 3, 6, 14, 15,** 22, 23, **33,** **41, 43, 49** | A7-I | 1 | 14 | 2, **3,** 4, **6,** 8, 11, 12, **14,** **15,** **33,** 36, 39, **41, 43,** 48, **49** |
| 7 | 3^b^ | 2 | 1, **2, 6, 14, 15,** 22, 23, **33,** **41, 43, 49** | A7-I | 1 | 14 | **2,** 3, 4, **6,** 8, 11, 12, **14, 15,** **33,** 36, 39, **41, 43,** 48, **49** |
| 7 | 6^b^ | 2 | 1, **2, 3, 14, 15,** 22, 23, **33,** **41, 43, 49** | A7-I | 1 | 14 | **2, 3,** 4, 6, 8, 11, 12, **14, 15, 33,** 36, 39, **41, 43**, 48, **49** |
| 9 | ^a^ | ^a^ | 24, 34, 35 | ^d^ | ^d^ | ^d^ | ^d^ |
| 12 | ^a^ | ^a^ | 13, 40, 47 | ^d^ | ^d^ | ^d^ | ^d^ |

**DV:** dominant variant

**sDV:** subdominant variant

**SLV:** single locus variant

^a^ PE did not contain a dominant variant.

^b^subdominant variant of PE.

^c^ PE did not contain any other variants.

^d^ PE ST variants do not appear within a clonal complex

## Supplementary Table 24. Comparison of singleton STs surrounding dominant variants of PEs and consensus sequences of clonal complexes observed in 5-locus MLSA of A-like *Synechococcus* BACs (49 sequences). STs common to a PE and a corresponding clonal complex are highlighted in bold. Note: STs designated in the A-like *Synechococcus* population 5 locus MLSA *do not* correspond to the STs in the 7 locus MLSA.

| **PE** | **DV-ST** | **No. of Sequences of DV/sDV-ST** | **Variant STs within PE** | **Clonal Complex** | **Consensus ST** | **No. of Sequences with consensus ST** | **STs (SLVs) within a Clonal complex** |
| --- | --- | --- | --- | --- | --- | --- | --- |
| 1 | ^a^ | ^a^ | 25 | ^d^ | ^d^ | ^d^ | ^d^ |
| 2 | ^a^ | ^a^ | 13 | ^d^ | ^d^ | ^d^ | ^d^ |
| 3^b^ | ^a^ | ^a^ | 7, 19, 24 | ^d^ | ^d^ | ^d^ | ^d^ |
| 4 | ^a^ | ^a^ | 9 | ^d^ | ^d^ | ^d^ | ^d^ |
| 5 | ^a^ | ^a^ | 23 | ^d^ | ^d^ | ^d^ | ^d^ |
| 6 | ^a^ | ^a^ | 12 | ^d^ | ^d^ | ^d^ | ^d^ |
| 7 | ^a^ | ^a^ | 10 | ^d^ | ^d^ | ^d^ | ^d^ |
| 8^b^ | 2 | 5 | e | ^d^ | ^d^ | ^d^ | ^d^ |
| 9 | 5 | 2 | **14,** 15, **16,** 18, 21, 26 | A5-i | 5 | 2 | **14, 16** |
| 10 | 3 | 4 | **11** | A5-ii | 3 | 4 | 7, **11** |
| 11 | ^a^ | ^a^ | 27 | ^d^ | ^d^ | ^d^ | ^d^ |
| 12^b^ | ^a^ | ^a^ | 20, 22 | ^d^ | ^d^ | ^d^ | ^d^ |
| 13^c^ | 1 | 13 | **4, 6** | A5-I | 1 | 13 | **4, 6** |
| 13^c^ | 4^f^ | 2 | 1, **6** | A5-I | 1 | 13 | 4, **6** |
| 13^c^ | 6^f^ | 2 | 1, **4** | A5-I | 1 | 13 | **4,** 6 |

**sDV:** subdominant variant

**SLV:** single locus variant

^a^ PE did not contain a dominant variant.

^b^ 65^o^C -specific PE

^c^ PE13 contained 1 dominant variant representing 13 sequences and 2 subdominant variants represented by 2 sequences each.

^d^ PE ST variants do not appear within a clonal complex.

^e^PE ST variants appear within a sub-clonal complex (relaxed criterion of 2 single-locus variants).

^f^ sDV of PE13.

Supplementary Table 25. Pairwise percent nucleotide identity comparisons among A-like *Synechococcus* isolate genomes (see ref ([10](#_ENREF_10)) for details). Above the diagonal is the percent nucleotide identity for each pair of genomes calculated from an 1136 orthologous gene concatenation. Below the diagonal is the percent nucleotide identity for each pair of genomes calculated from the whole genome using reciprocal best hits. Bold text indicates within-PE comparisons.

|  | A1-OS | A1-MS | A4 | A6-63 | A14-60 |
| --- | --- | --- | --- | --- | --- |
| A1-OS | - | **99.32%** | 98.96% | 99.02% | 98.87% |
| A1-MS | **99.32%** | - | 98.92% | 98.99% | 98.84% |
| A4 | 98.35% | 98.72% | - | 99.53% | 99.50% |
| A6-63 | 98.49% | 98.39% | 99.72% | - | 99.57% |
| A14-60 | 98.46% | 98.41% | 99.05% | 99.11% | - |

**References**

1. Melendrez MC, Lange RK, Cohan FM, Ward DM. Influence of molecular resolution on sequence-based discovery of ecological diversity among *Synechococcus* populations in an alkaline siliceous hot spring microbial mat. Appl Environ Microb. 2011;77(4):1359-67.

2. Klatt CG, Wood JM, Rusch DB, Bateson MM, Hamamura N, Heidelberg JF, et al. Community ecology of hot spring cyanobacterial mats: predominant populations and their functional potential. Isme J. 2011;5(8):1262-78.

3. Rusch DB, Halpern AL, Sutton G, Heidelberg KB, Williamson S, Yooseph S, et al. The Sorcerer II global ocean sampling expedition: Northwest Atlantic through Eastern Tropical Pacific. PLoS biology. 2007;5(3):398-431.

4. Didelot X, Falush D. Inference of bacterial microevolution using multilocus sequence data. Genetics. 2007;175(3):1251-66.

5. Martin DP, Lemey P, Lott M, Moulton V, Posada D, Lefeuvre P. RDP3: a flexible and fast computer program for analyzing recombination. Bioinformatics. 2010;26(19):2462-3.

6. Nei M, Kumar S. Molecular Evolution and Phylogenetics. New York: Oxford University Press; 2000.

7. Tamura K, Nei M, Kumar S. Prospects for inferring very large phylogenies by using the neighbor-joining method. P Natl Acad Sci USA. 2004;101(30):11030-5.

8. Tamura K, Dudley J, Nei M, Kumar S. MEGA4: Molecular evolutionary genetics analysis (MEGA) software version 4.0. Mol Biol Evol. 2007;24(8):1596-9.

9. Haubold B, Hudson RR. LIAN 3.0: detecting linkage disequilibrium in multilocus data. Linkage Analysis. Bioinformatics. 2000;16(9):847-8.

10. Olsen MT, Nowack S, Wood JM, Becraft ED, LaButti K, Lipzen A, et al. The molecular dimension of microbial species: 3. Comparative genomics of *Synechoccocus* isolates with different light responses and *in situ* diel transcription patterns of associated putative ecotypes in the Mushroom Spring microbial mat. Frontiers in Microbiology. 2015;6:604.
