# Supplementary material for: Recombination Does Not Hinder Formation or Detection of Ecological Species of Synechococcus Inhabiting a Hot Spring Cyanobacterial Mat
Source: Front Microbiol. 2016 Jan 14;6:1540. doi: 10.3389/fmicb.2015.01540 (PMC4712262; doi:10.3389/fmicb.2015.01540)
Supplement: Supplementary file 2 [file Presentation1.pptx]

## Slide 1
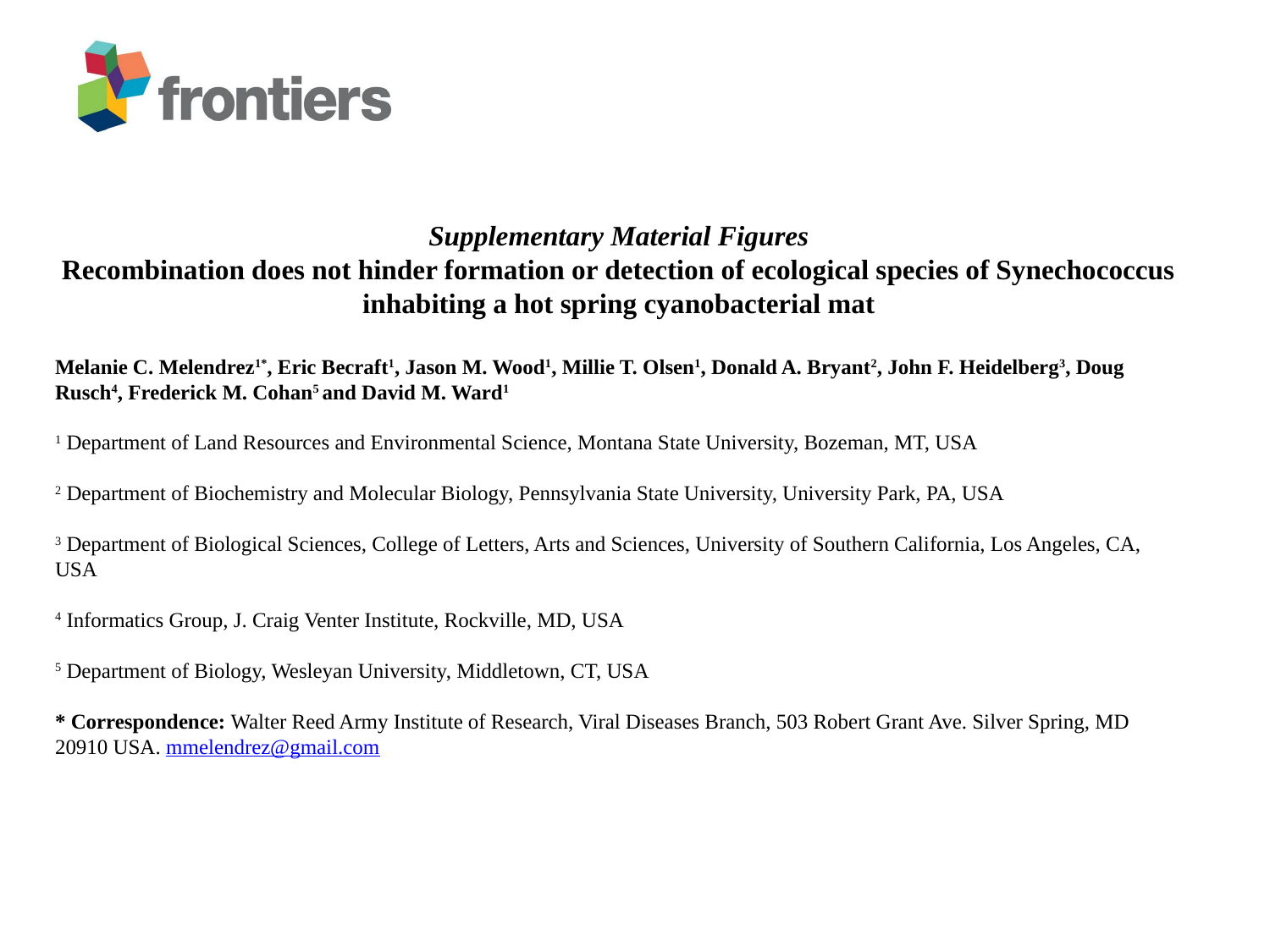

Supplementary Material Figures
Recombination does not hinder formation or detection of ecological species of Synechococcus inhabiting a hot spring cyanobacterial mat
Melanie C. Melendrez1*, Eric Becraft1, Jason M. Wood1, Millie T. Olsen1, Donald A. Bryant2, John F. Heidelberg3, Doug Rusch4, Frederick M. Cohan5 and David M. Ward1
1 Department of Land Resources and Environmental Science, Montana State University, Bozeman, MT, USA
2 Department of Biochemistry and Molecular Biology, Pennsylvania State University, University Park, PA, USA
3 Department of Biological Sciences, College of Letters, Arts and Sciences, University of Southern California, Los Angeles, CA, USA
4 Informatics Group, J. Craig Venter Institute, Rockville, MD, USA
5 Department of Biology, Wesleyan University, Middletown, CT, USA
* Correspondence: Walter Reed Army Institute of Research, Viral Diseases Branch, 503 Robert Grant Ave. Silver Spring, MD 20910 USA. mmelendrez@gmail.com

## Slide 2
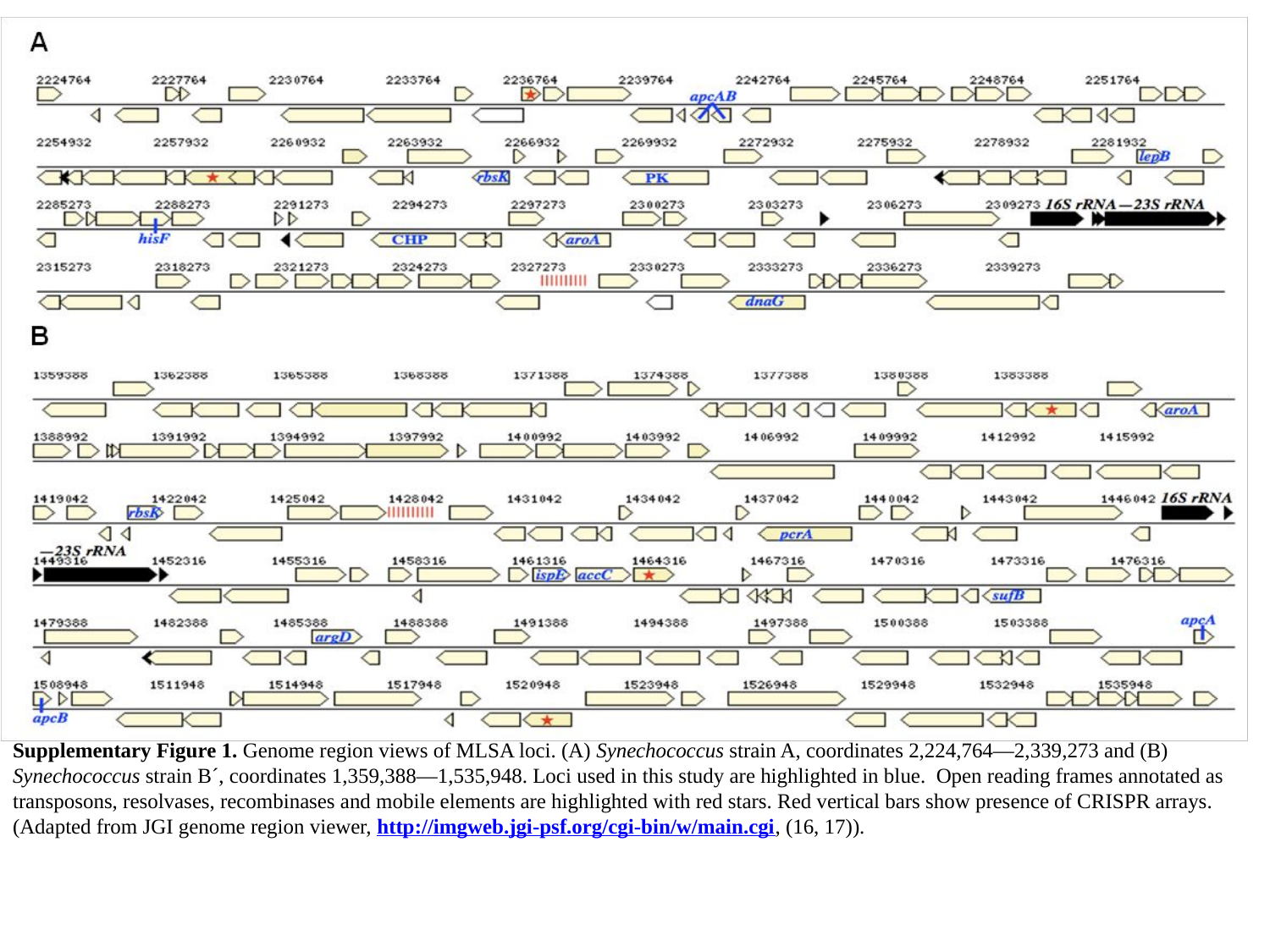

Supplementary Figure 1. Genome region views of MLSA loci. (A) Synechococcus strain A, coordinates 2,224,764—2,339,273 and (B) Synechococcus strain B´, coordinates 1,359,388—1,535,948. Loci used in this study are highlighted in blue. Open reading frames annotated as transposons, resolvases, recombinases and mobile elements are highlighted with red stars. Red vertical bars show presence of CRISPR arrays. (Adapted from JGI genome region viewer, http://imgweb.jgi-psf.org/cgi-bin/w/main.cgi, (16, 17)).

## Slide 3
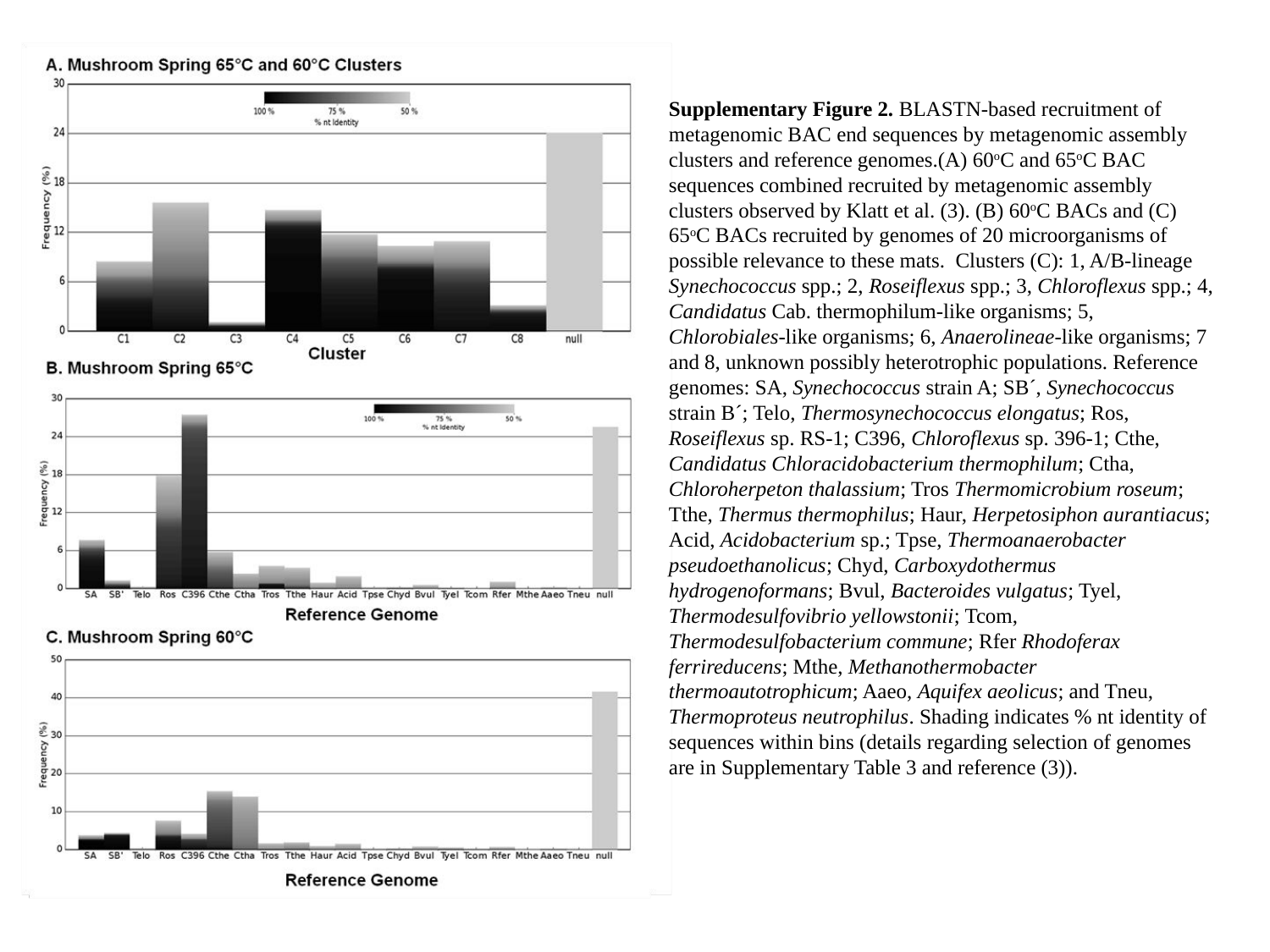

Supplementary Figure 2. BLASTN-based recruitment of metagenomic BAC end sequences by metagenomic assembly clusters and reference genomes.(A) 60oC and 65oC BAC sequences combined recruited by metagenomic assembly clusters observed by Klatt et al. (3). (B) 60oC BACs and (C) 65oC BACs recruited by genomes of 20 microorganisms of possible relevance to these mats. Clusters (C): 1, A/B-lineage Synechococcus spp.; 2, Roseiflexus spp.; 3, Chloroflexus spp.; 4, Candidatus Cab. thermophilum-like organisms; 5, Chlorobiales-like organisms; 6, Anaerolineae-like organisms; 7 and 8, unknown possibly heterotrophic populations. Reference genomes: SA, Synechococcus strain A; SB´, Synechococcus strain B´; Telo, Thermosynechococcus elongatus; Ros, Roseiflexus sp. RS-1; C396, Chloroflexus sp. 396-1; Cthe, Candidatus Chloracidobacterium thermophilum; Ctha, Chloroherpeton thalassium; Tros Thermomicrobium roseum; Tthe, Thermus thermophilus; Haur, Herpetosiphon aurantiacus; Acid, Acidobacterium sp.; Tpse, Thermoanaerobacter pseudoethanolicus; Chyd, Carboxydothermus hydrogenoformans; Bvul, Bacteroides vulgatus; Tyel, Thermodesulfovibrio yellowstonii; Tcom, Thermodesulfobacterium commune; Rfer Rhodoferax ferrireducens; Mthe, Methanothermobacter thermoautotrophicum; Aaeo, Aquifex aeolicus; and Tneu, Thermoproteus neutrophilus. Shading indicates % nt identity of sequences within bins (details regarding selection of genomes are in Supplementary Table 3 and reference (3)).

## Slide 4
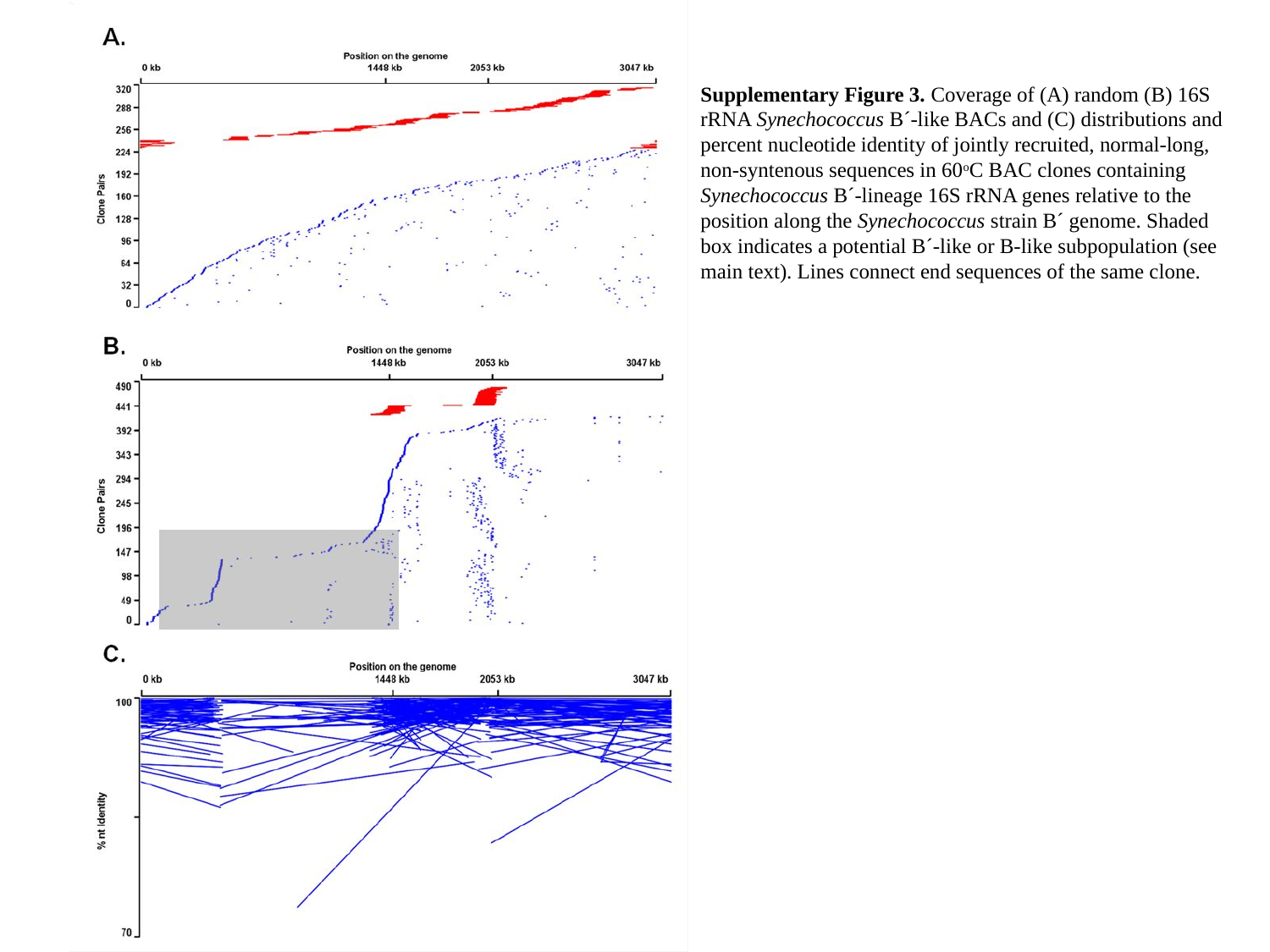

Supplementary Figure 3. Coverage of (A) random (B) 16S rRNA Synechococcus B´-like BACs and (C) distributions and percent nucleotide identity of jointly recruited, normal-long, non-syntenous sequences in 60oC BAC clones containing Synechococcus B´-lineage 16S rRNA genes relative to the position along the Synechococcus strain B´ genome. Shaded box indicates a potential B´-like or B-like subpopulation (see main text). Lines connect end sequences of the same clone.

## Slide 5
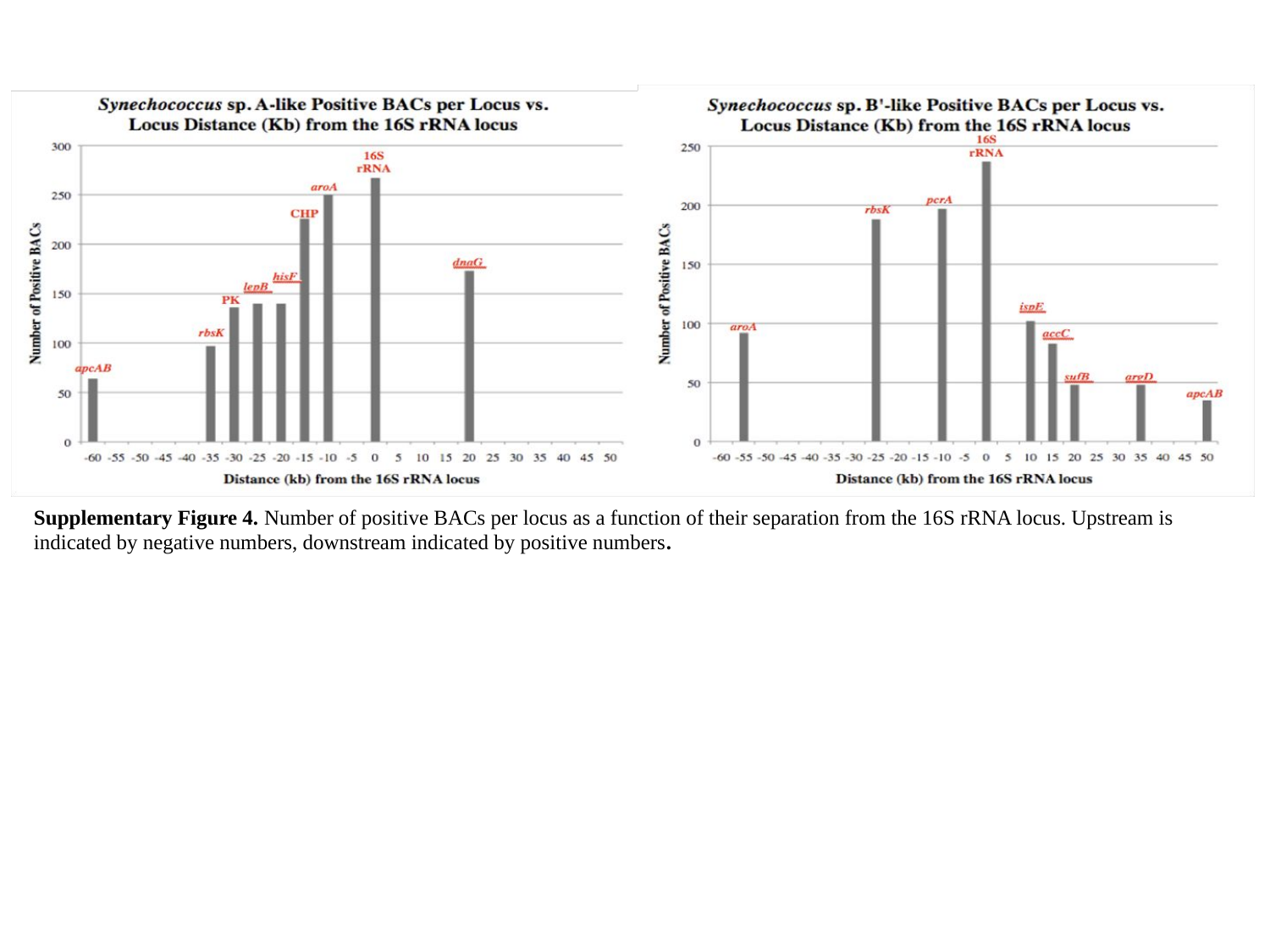

Supplementary Figure 4. Number of positive BACs per locus as a function of their separation from the 16S rRNA locus. Upstream is indicated by negative numbers, downstream indicated by positive numbers.

## Slide 6
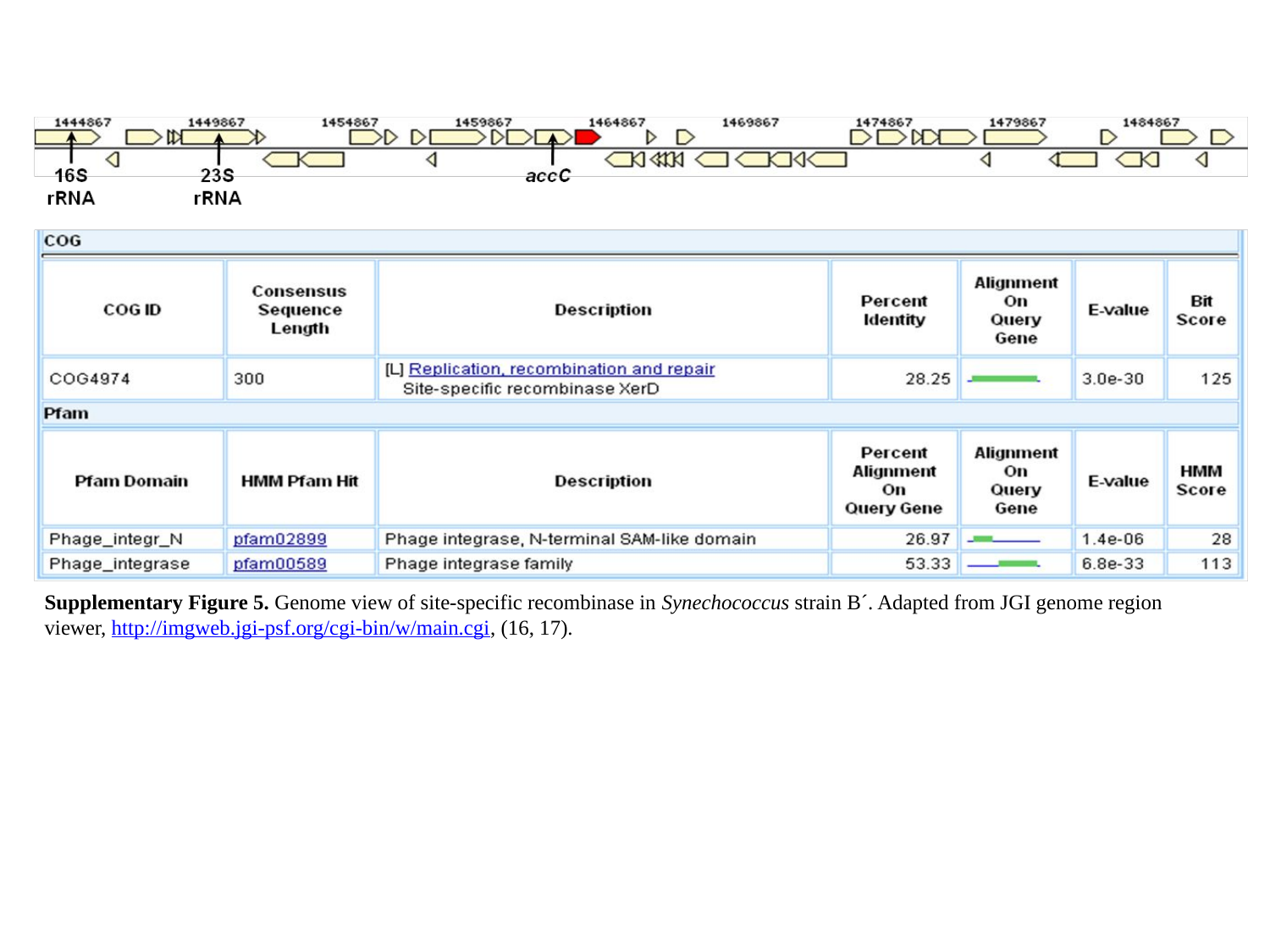

Supplementary Figure 5. Genome view of site-specific recombinase in Synechococcus strain B´. Adapted from JGI genome region viewer, http://imgweb.jgi-psf.org/cgi-bin/w/main.cgi, (16, 17).

## Slide 7
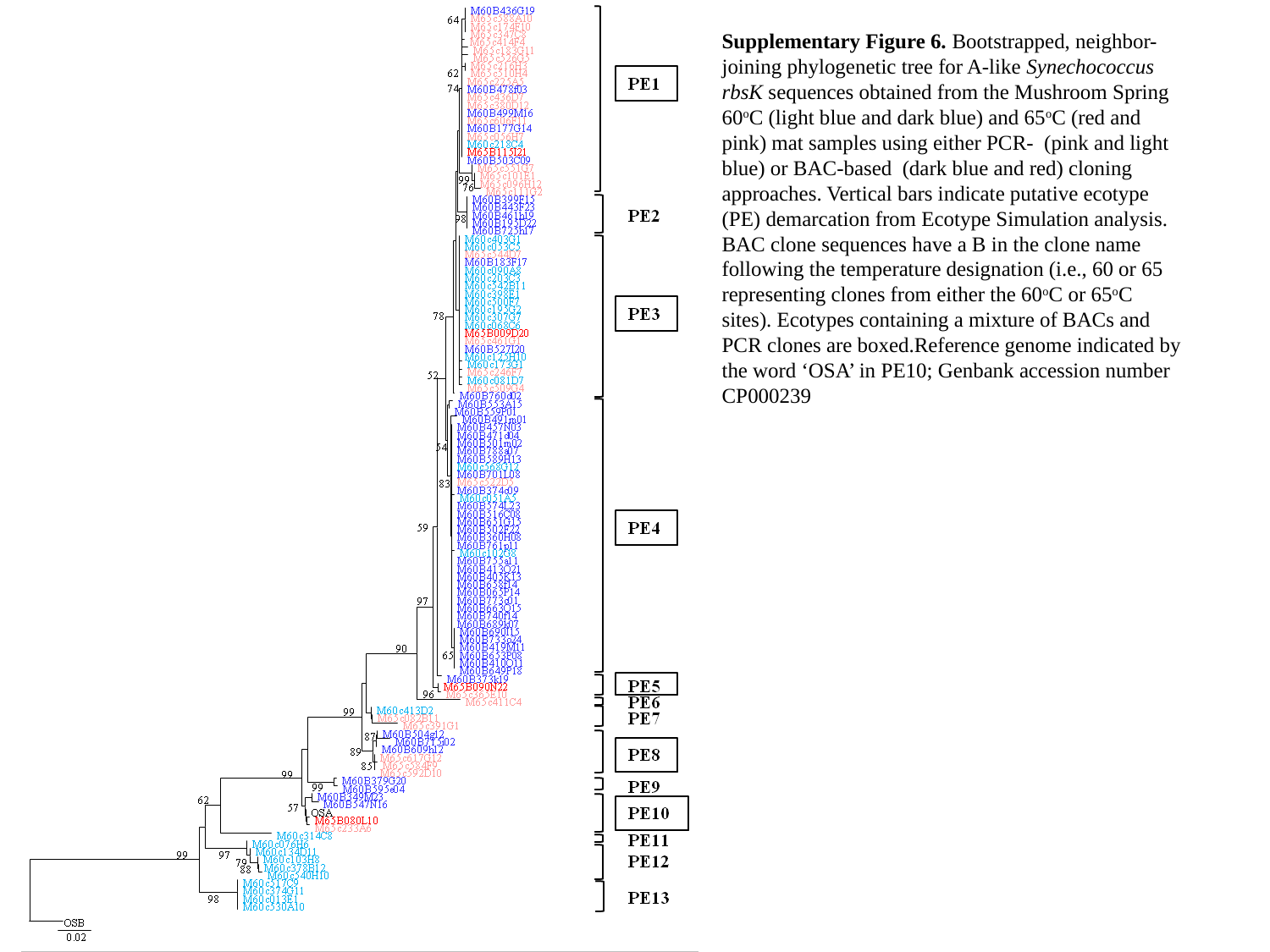

Supplementary Figure 6. Bootstrapped, neighbor-joining phylogenetic tree for A-like Synechococcus rbsK sequences obtained from the Mushroom Spring 60oC (light blue and dark blue) and 65oC (red and pink) mat samples using either PCR- (pink and light blue) or BAC-based (dark blue and red) cloning approaches. Vertical bars indicate putative ecotype (PE) demarcation from Ecotype Simulation analysis. BAC clone sequences have a B in the clone name following the temperature designation (i.e., 60 or 65 representing clones from either the 60oC or 65oC sites). Ecotypes containing a mixture of BACs and PCR clones are boxed.Reference genome indicated by the word ‘OSA’ in PE10; Genbank accession number CP000239

## Slide 8
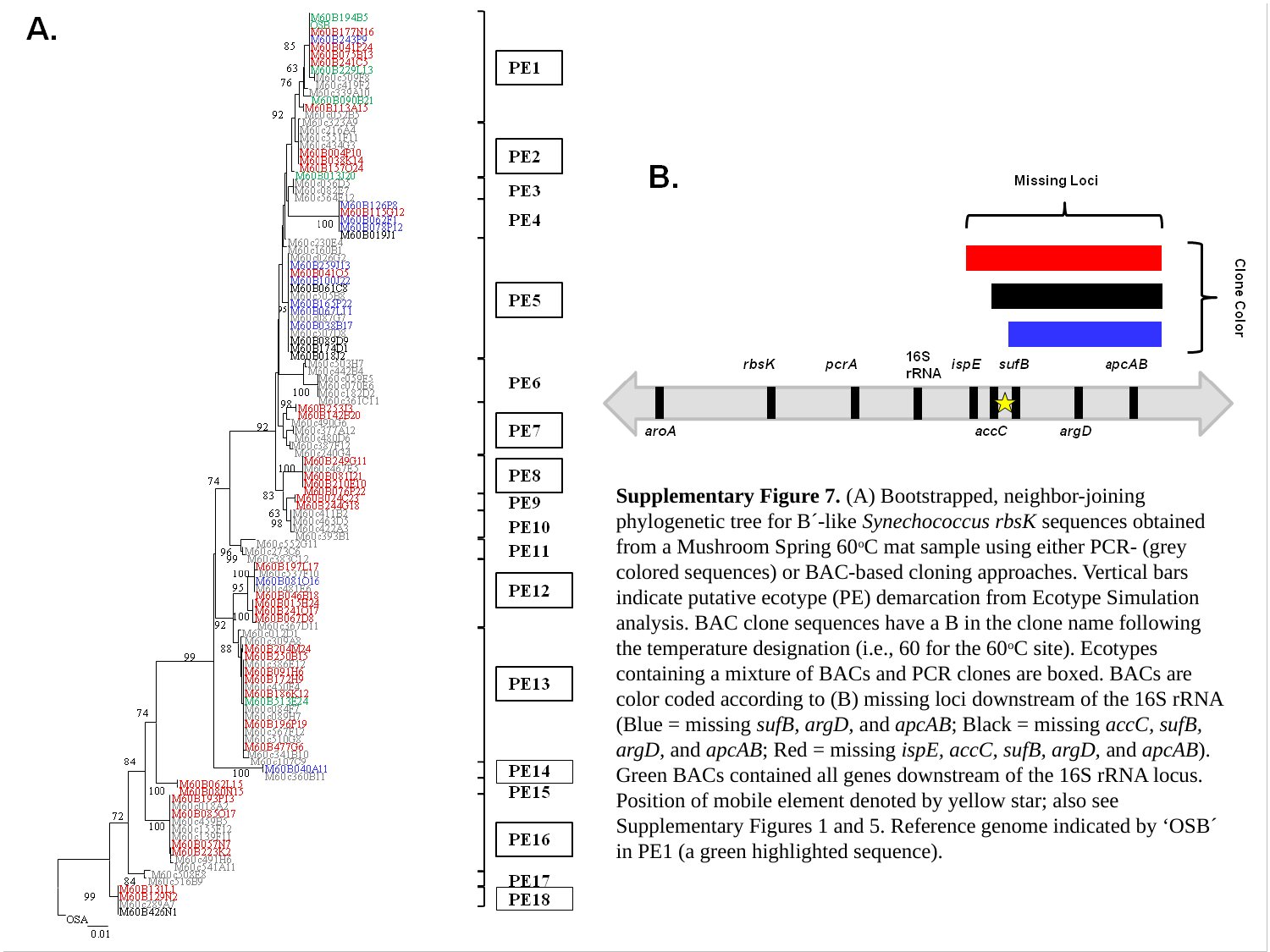

Supplementary Figure 7. (A) Bootstrapped, neighbor-joining phylogenetic tree for B´-like Synechococcus rbsK sequences obtained from a Mushroom Spring 60oC mat sample using either PCR- (grey colored sequences) or BAC-based cloning approaches. Vertical bars indicate putative ecotype (PE) demarcation from Ecotype Simulation analysis. BAC clone sequences have a B in the clone name following the temperature designation (i.e., 60 for the 60oC site). Ecotypes containing a mixture of BACs and PCR clones are boxed. BACs are color coded according to (B) missing loci downstream of the 16S rRNA (Blue = missing sufB, argD, and apcAB; Black = missing accC, sufB, argD, and apcAB; Red = missing ispE, accC, sufB, argD, and apcAB). Green BACs contained all genes downstream of the 16S rRNA locus. Position of mobile element denoted by yellow star; also see Supplementary Figures 1 and 5. Reference genome indicated by ‘OSB´ in PE1 (a green highlighted sequence).

## Slide 9
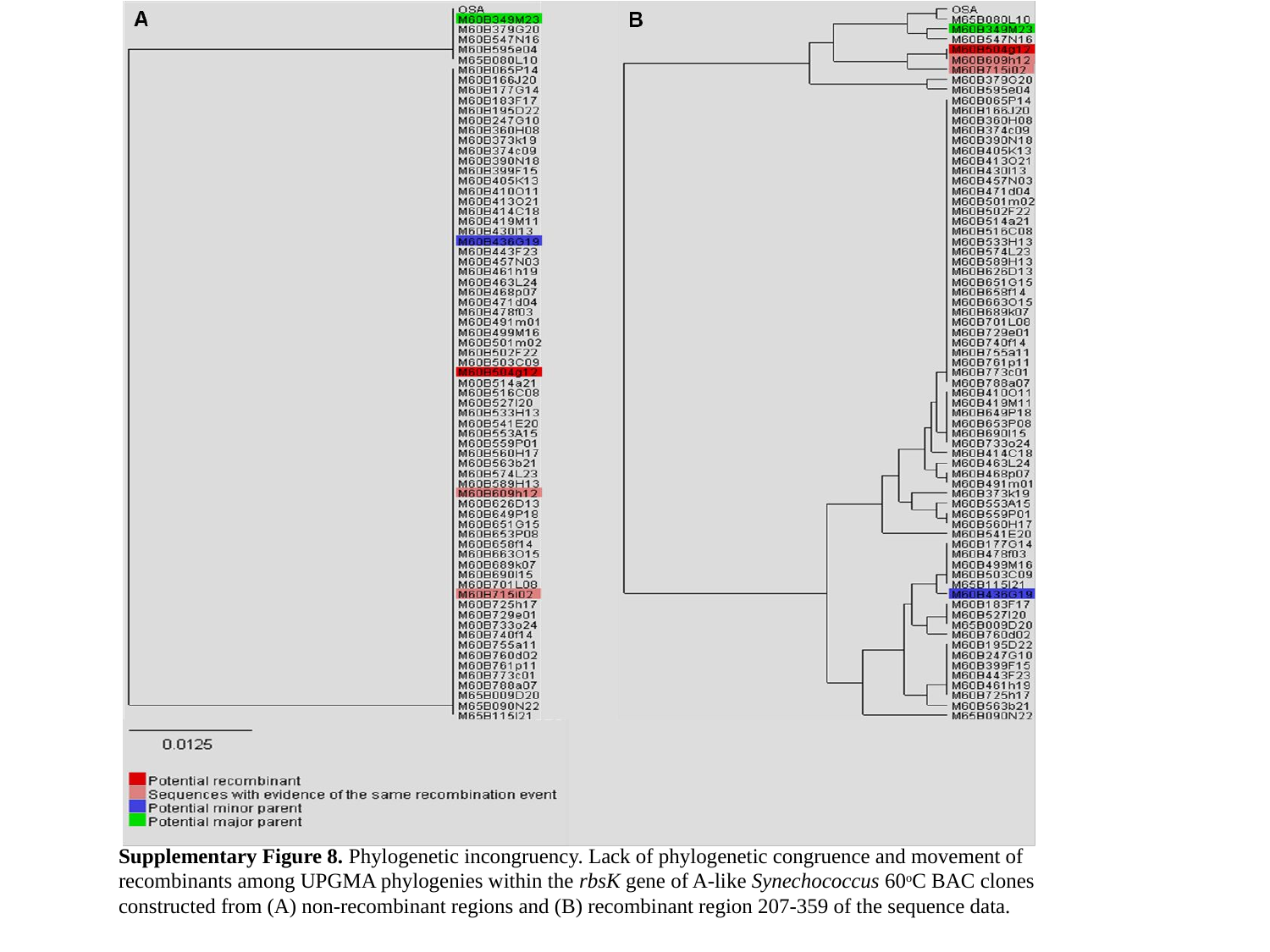

Supplementary Figure 8. Phylogenetic incongruency. Lack of phylogenetic congruence and movement of recombinants among UPGMA phylogenies within the rbsK gene of A-like Synechococcus 60oC BAC clones constructed from (A) non-recombinant regions and (B) recombinant region 207-359 of the sequence data.

## Slide 10
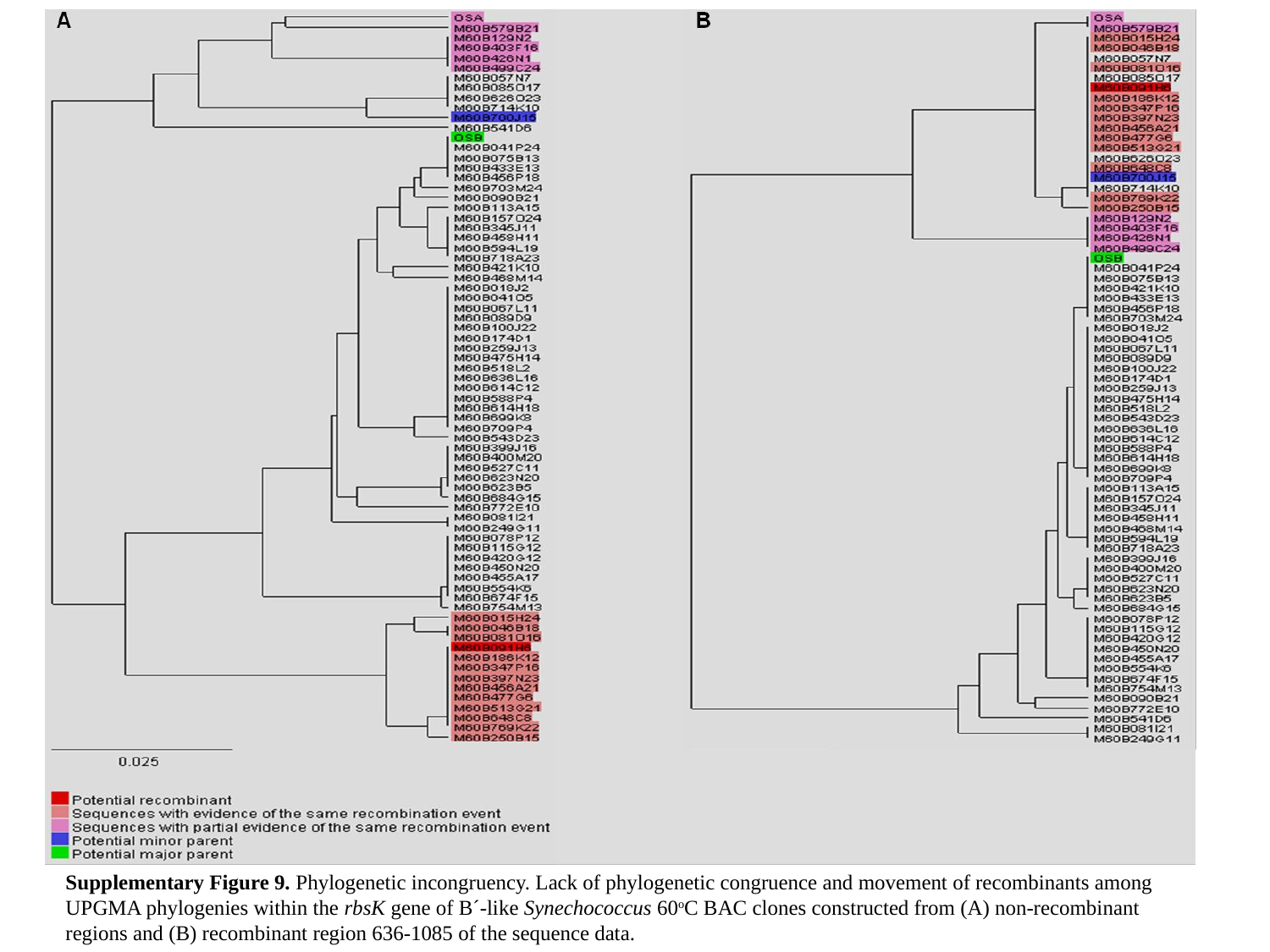

Supplementary Figure 9. Phylogenetic incongruency. Lack of phylogenetic congruence and movement of recombinants among UPGMA phylogenies within the rbsK gene of B´-like Synechococcus 60oC BAC clones constructed from (A) non-recombinant regions and (B) recombinant region 636-1085 of the sequence data.

## Slide 11
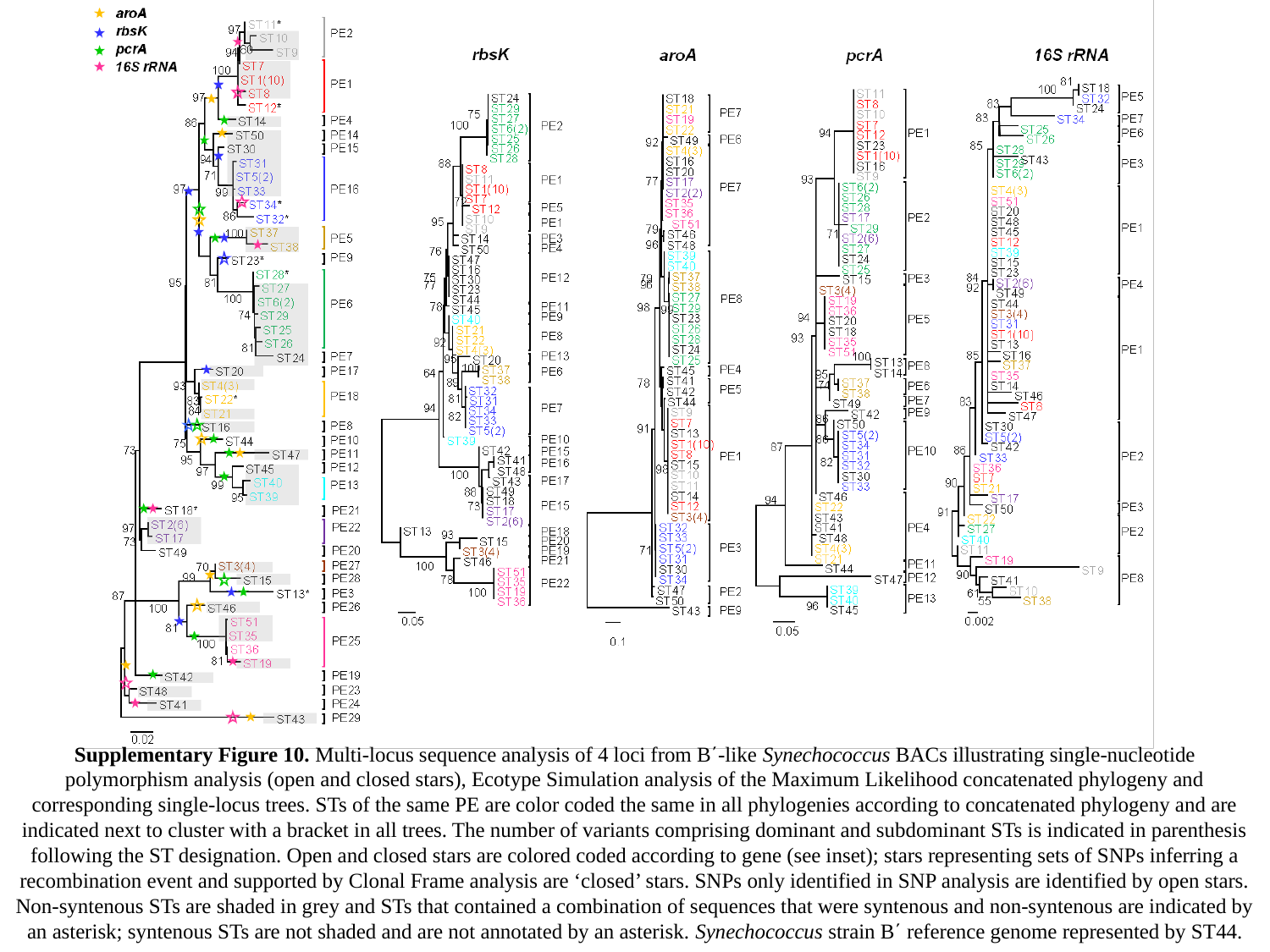

Supplementary Figure 10. Multi-locus sequence analysis of 4 loci from B΄-like Synechococcus BACs illustrating single-nucleotide polymorphism analysis (open and closed stars), Ecotype Simulation analysis of the Maximum Likelihood concatenated phylogeny and corresponding single-locus trees. STs of the same PE are color coded the same in all phylogenies according to concatenated phylogeny and are indicated next to cluster with a bracket in all trees. The number of variants comprising dominant and subdominant STs is indicated in parenthesis following the ST designation. Open and closed stars are colored coded according to gene (see inset); stars representing sets of SNPs inferring a recombination event and supported by Clonal Frame analysis are ‘closed’ stars. SNPs only identified in SNP analysis are identified by open stars. Non-syntenous STs are shaded in grey and STs that contained a combination of sequences that were syntenous and non-syntenous are indicated by an asterisk; syntenous STs are not shaded and are not annotated by an asterisk. Synechococcus strain B΄ reference genome represented by ST44.

## Slide 12
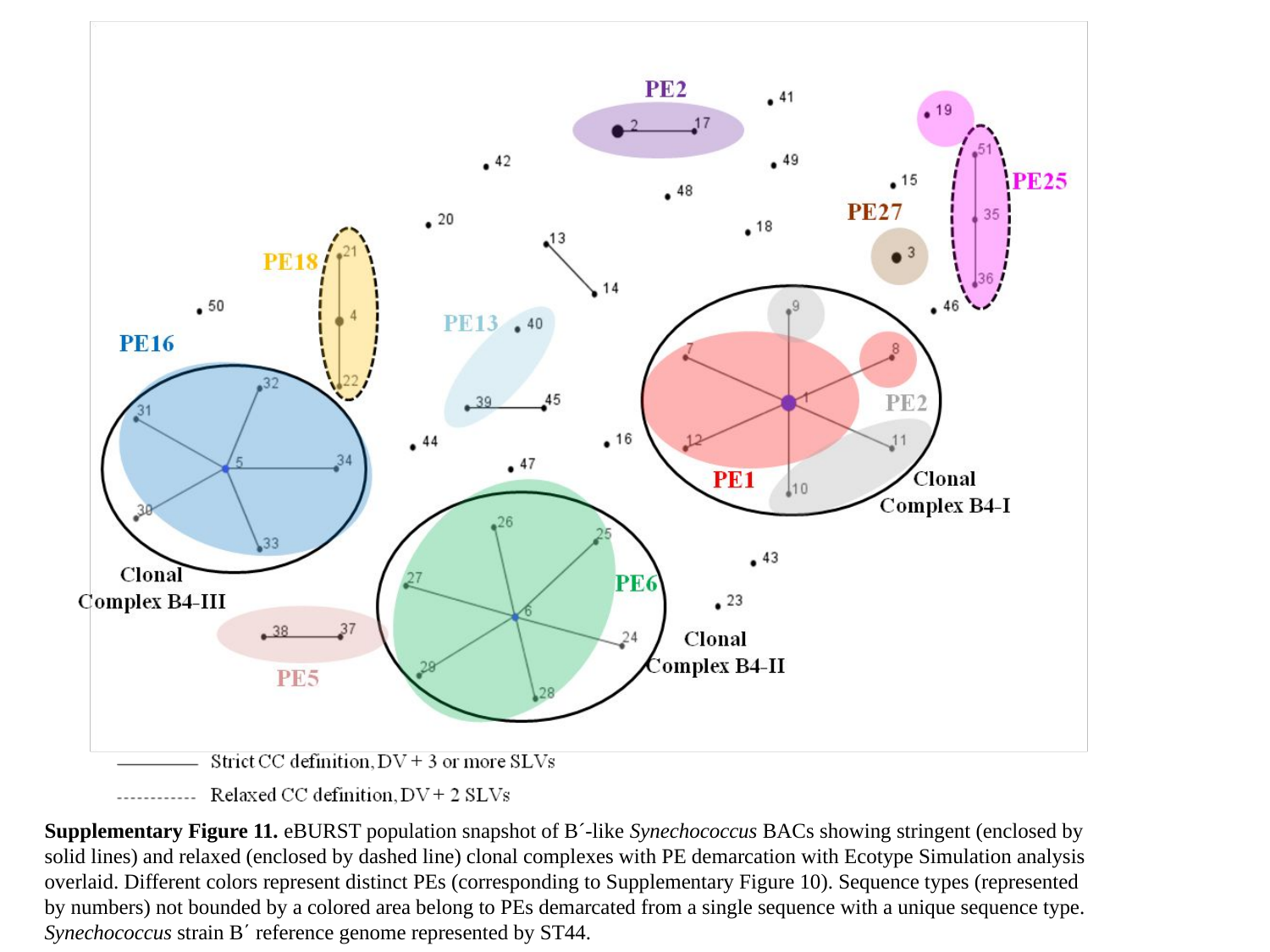

Supplementary Figure 11. eBURST population snapshot of B´-like Synechococcus BACs showing stringent (enclosed by solid lines) and relaxed (enclosed by dashed line) clonal complexes with PE demarcation with Ecotype Simulation analysis overlaid. Different colors represent distinct PEs (corresponding to Supplementary Figure 10). Sequence types (represented by numbers) not bounded by a colored area belong to PEs demarcated from a single sequence with a unique sequence type. Synechococcus strain B΄ reference genome represented by ST44.

## Slide 13
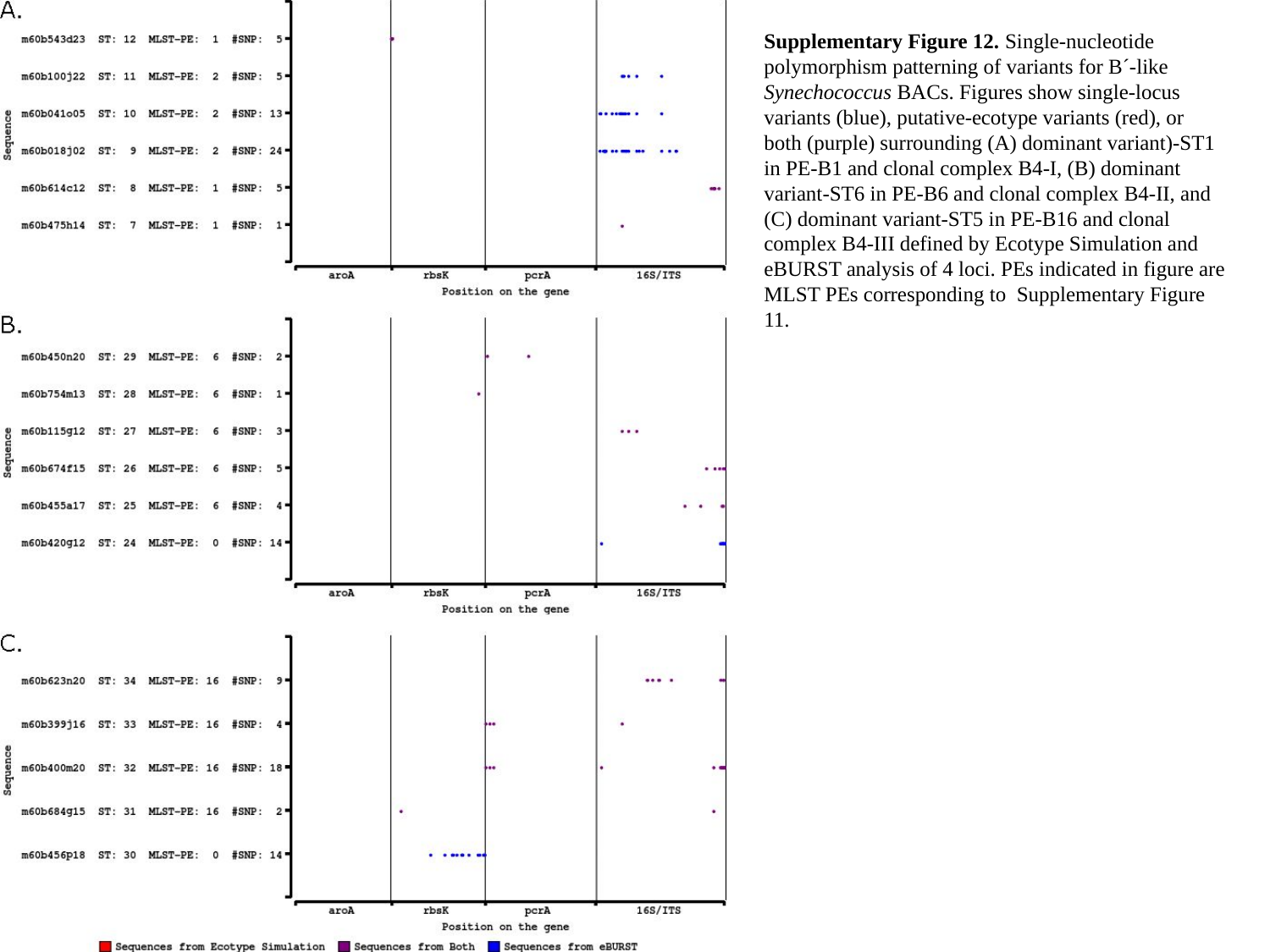

Supplementary Figure 12. Single-nucleotide polymorphism patterning of variants for B´-like Synechococcus BACs. Figures show single-locus variants (blue), putative-ecotype variants (red), or both (purple) surrounding (A) dominant variant)-ST1 in PE-B1 and clonal complex B4-I, (B) dominant variant-ST6 in PE-B6 and clonal complex B4-II, and (C) dominant variant-ST5 in PE-B16 and clonal complex B4-III defined by Ecotype Simulation and eBURST analysis of 4 loci. PEs indicated in figure are MLST PEs corresponding to Supplementary Figure 11.

## Slide 14
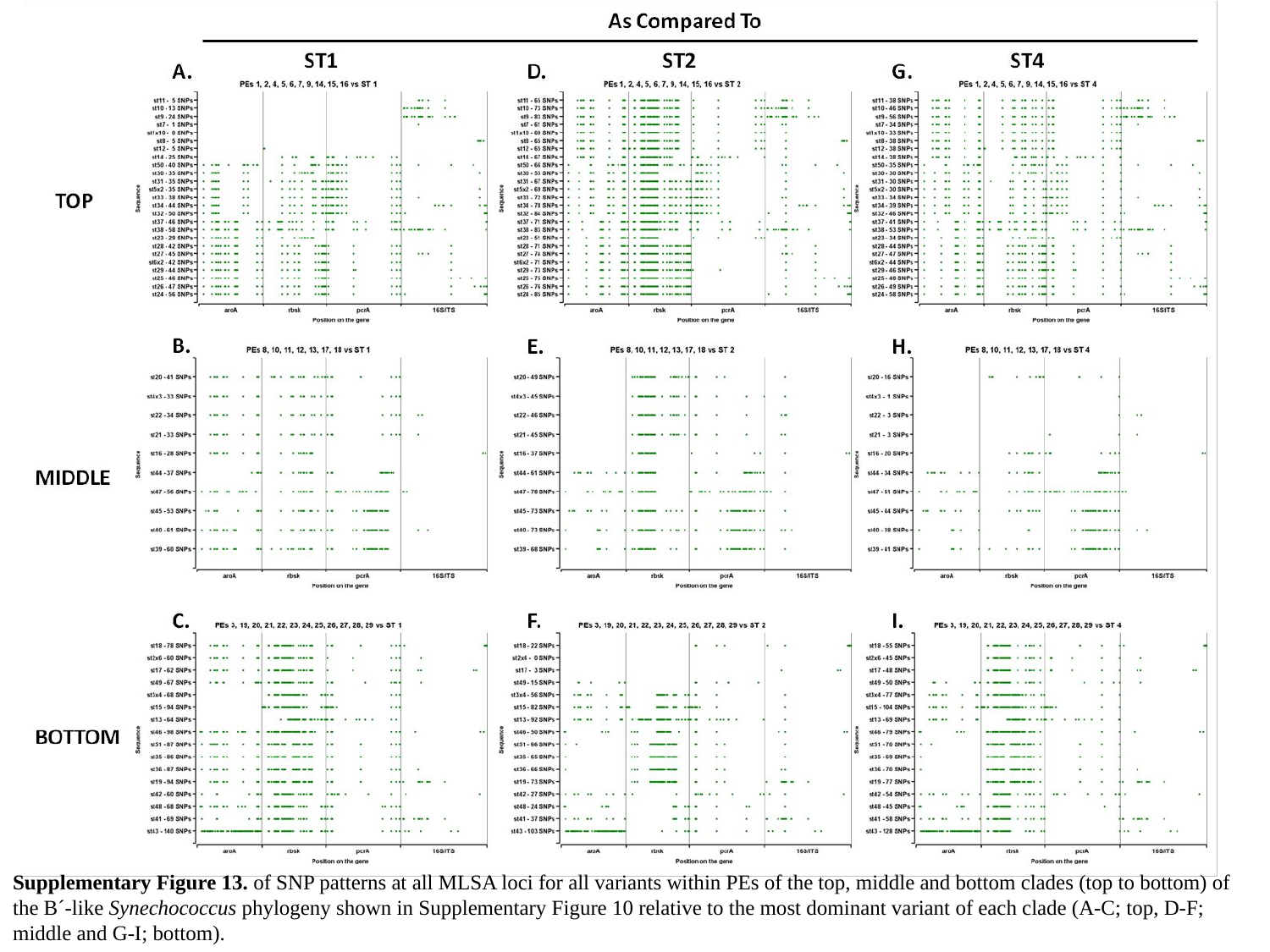

Supplementary Figure 13. of SNP patterns at all MLSA loci for all variants within PEs of the top, middle and bottom clades (top to bottom) of the B´-like Synechococcus phylogeny shown in Supplementary Figure 10 relative to the most dominant variant of each clade (A-C; top, D-F; middle and G-I; bottom).

## Slide 15
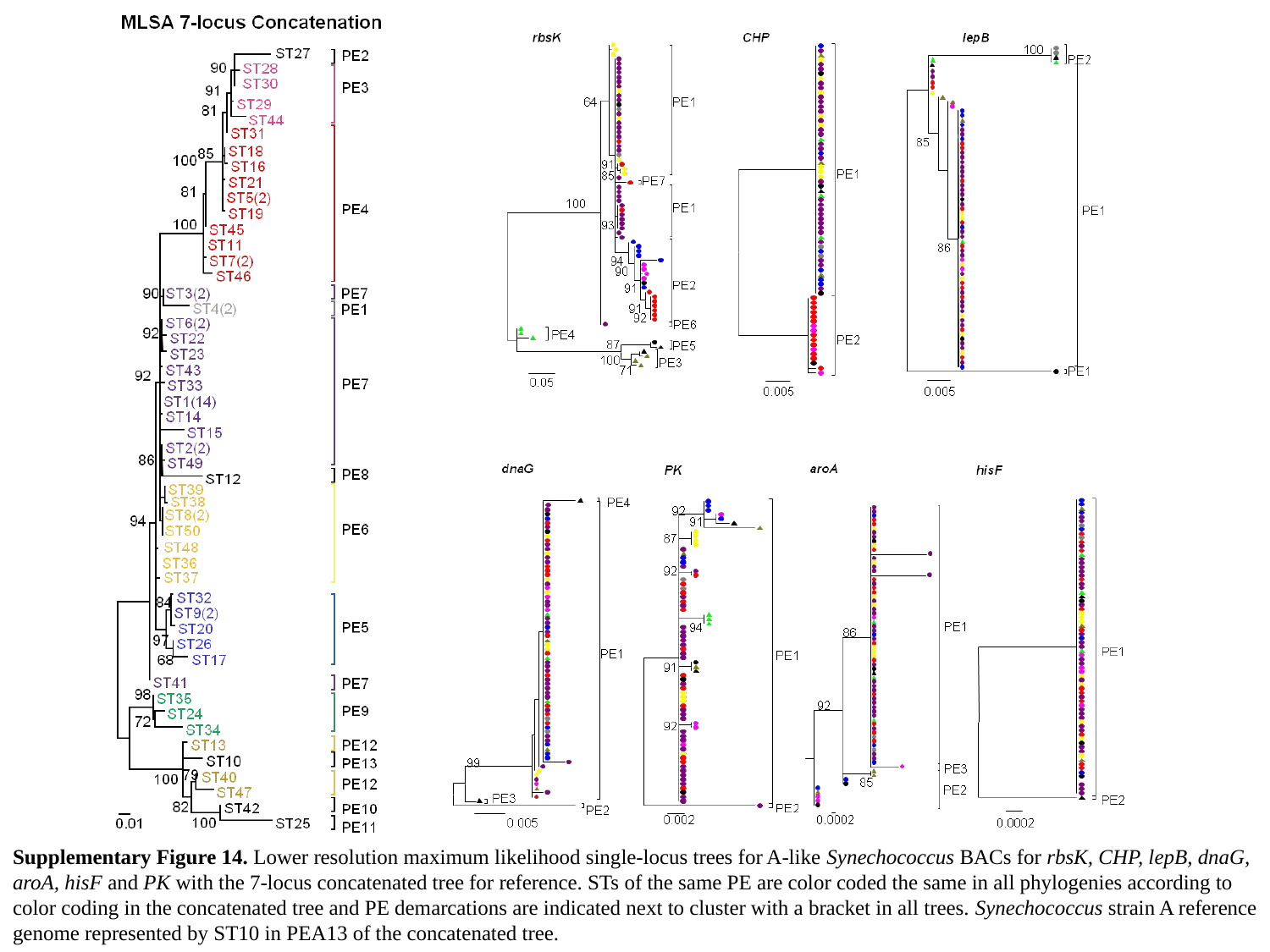

Supplementary Figure 14. Lower resolution maximum likelihood single-locus trees for A-like Synechococcus BACs for rbsK, CHP, lepB, dnaG, aroA, hisF and PK with the 7-locus concatenated tree for reference. STs of the same PE are color coded the same in all phylogenies according to color coding in the concatenated tree and PE demarcations are indicated next to cluster with a bracket in all trees. Synechococcus strain A reference genome represented by ST10 in PEA13 of the concatenated tree.

## Slide 16
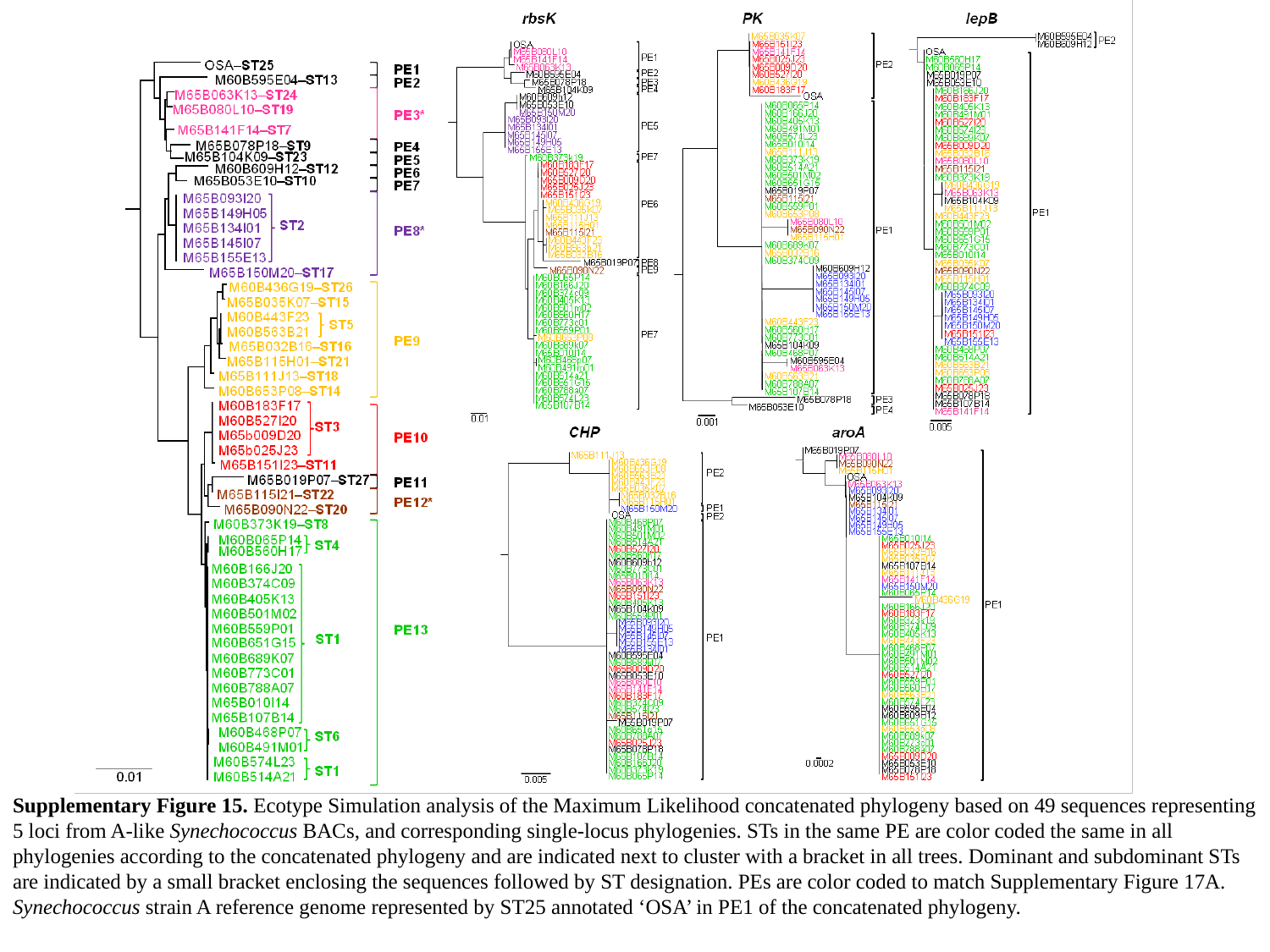

Supplementary Figure 15. Ecotype Simulation analysis of the Maximum Likelihood concatenated phylogeny based on 49 sequences representing 5 loci from A-like Synechococcus BACs, and corresponding single-locus phylogenies. STs in the same PE are color coded the same in all phylogenies according to the concatenated phylogeny and are indicated next to cluster with a bracket in all trees. Dominant and subdominant STs are indicated by a small bracket enclosing the sequences followed by ST designation. PEs are color coded to match Supplementary Figure 17A. Synechococcus strain A reference genome represented by ST25 annotated ‘OSA’ in PE1 of the concatenated phylogeny.

## Slide 17
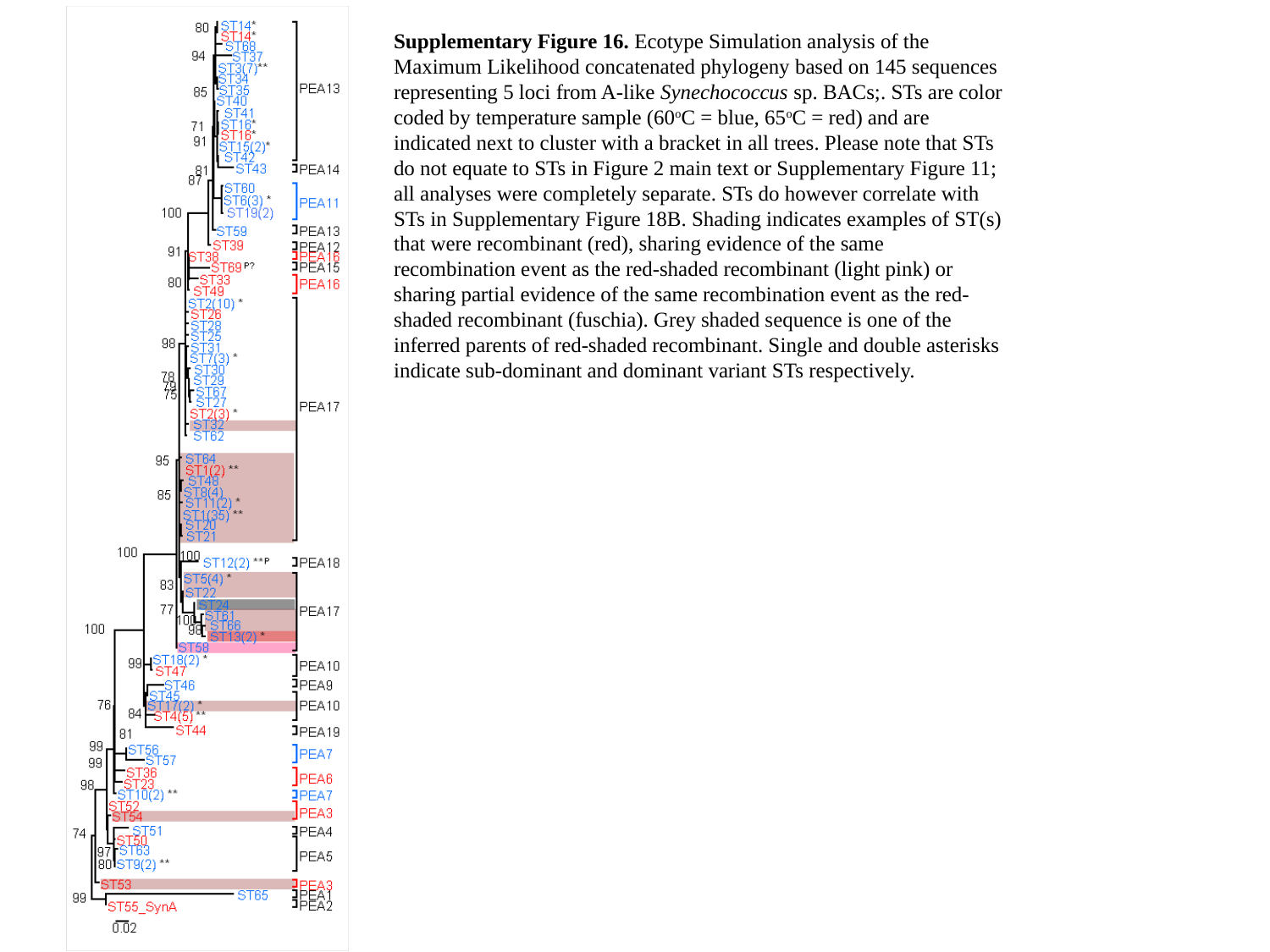

Supplementary Figure 16. Ecotype Simulation analysis of the Maximum Likelihood concatenated phylogeny based on 145 sequences representing 5 loci from A-like Synechococcus sp. BACs;. STs are color coded by temperature sample (60oC = blue, 65oC = red) and are indicated next to cluster with a bracket in all trees. Please note that STs do not equate to STs in Figure 2 main text or Supplementary Figure 11; all analyses were completely separate. STs do however correlate with STs in Supplementary Figure 18B. Shading indicates examples of ST(s) that were recombinant (red), sharing evidence of the same recombination event as the red-shaded recombinant (light pink) or sharing partial evidence of the same recombination event as the red-shaded recombinant (fuschia). Grey shaded sequence is one of the inferred parents of red-shaded recombinant. Single and double asterisks indicate sub-dominant and dominant variant STs respectively.

## Slide 18
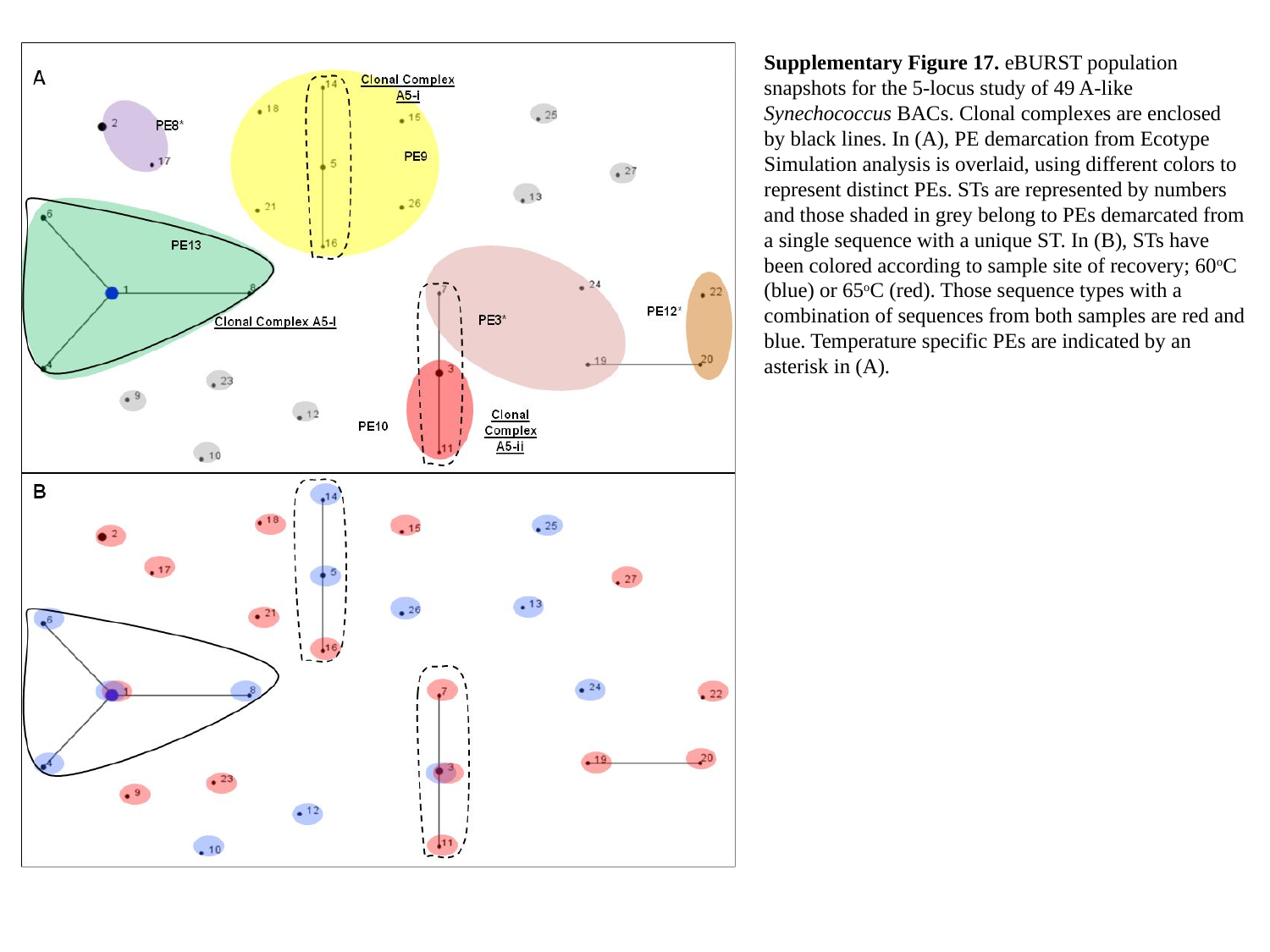

Supplementary Figure 17. eBURST population snapshots for the 5-locus study of 49 A-like Synechococcus BACs. Clonal complexes are enclosed by black lines. In (A), PE demarcation from Ecotype Simulation analysis is overlaid, using different colors to represent distinct PEs. STs are represented by numbers and those shaded in grey belong to PEs demarcated from a single sequence with a unique ST. In (B), STs have been colored according to sample site of recovery; 60oC (blue) or 65oC (red). Those sequence types with a combination of sequences from both samples are red and blue. Temperature specific PEs are indicated by an asterisk in (A).

## Slide 19
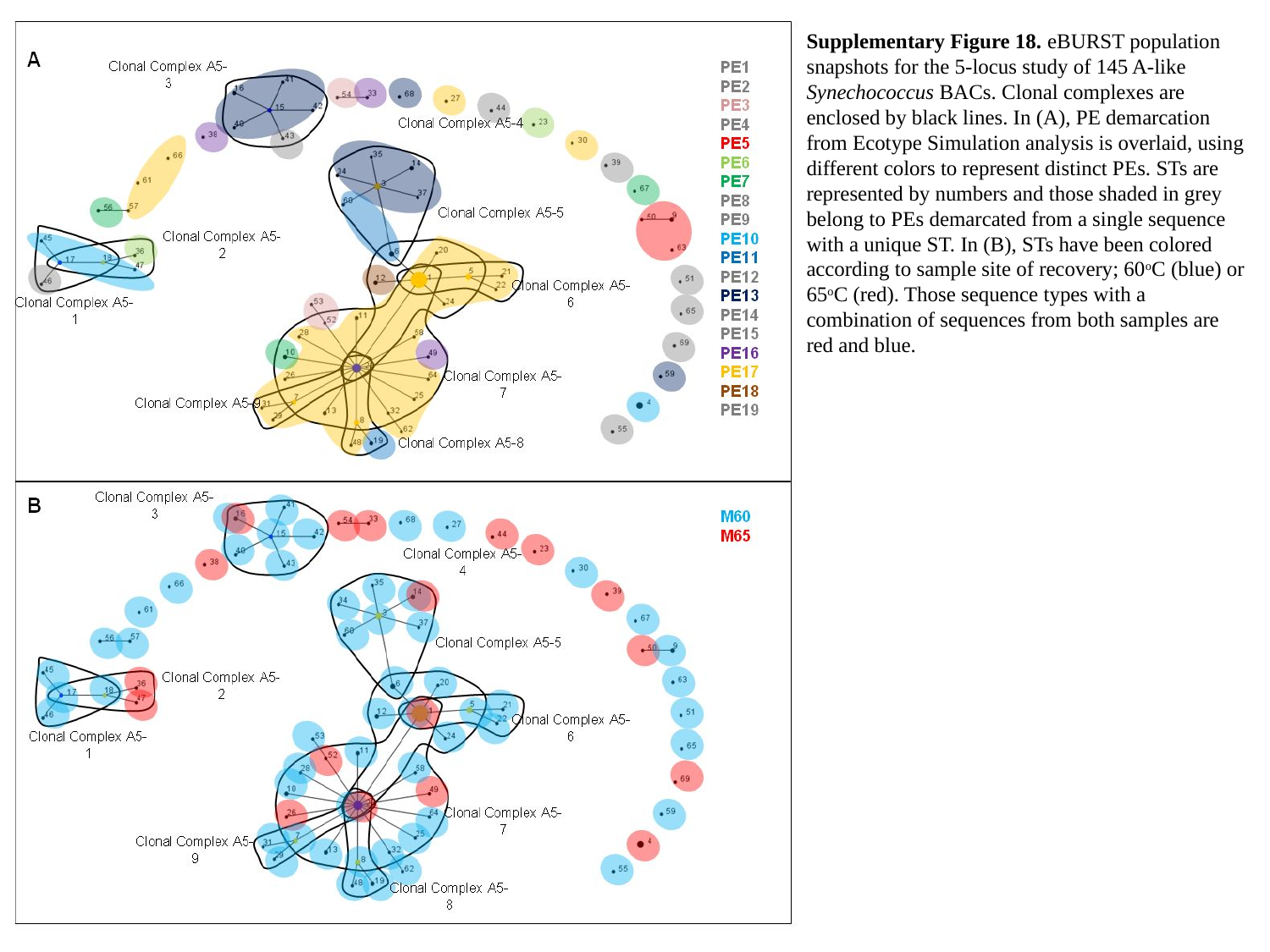

Supplementary Figure 18. eBURST population snapshots for the 5-locus study of 145 A-like Synechococcus BACs. Clonal complexes are enclosed by black lines. In (A), PE demarcation from Ecotype Simulation analysis is overlaid, using different colors to represent distinct PEs. STs are represented by numbers and those shaded in grey belong to PEs demarcated from a single sequence with a unique ST. In (B), STs have been colored according to sample site of recovery; 60oC (blue) or 65oC (red). Those sequence types with a combination of sequences from both samples are red and blue.

## Slide 20
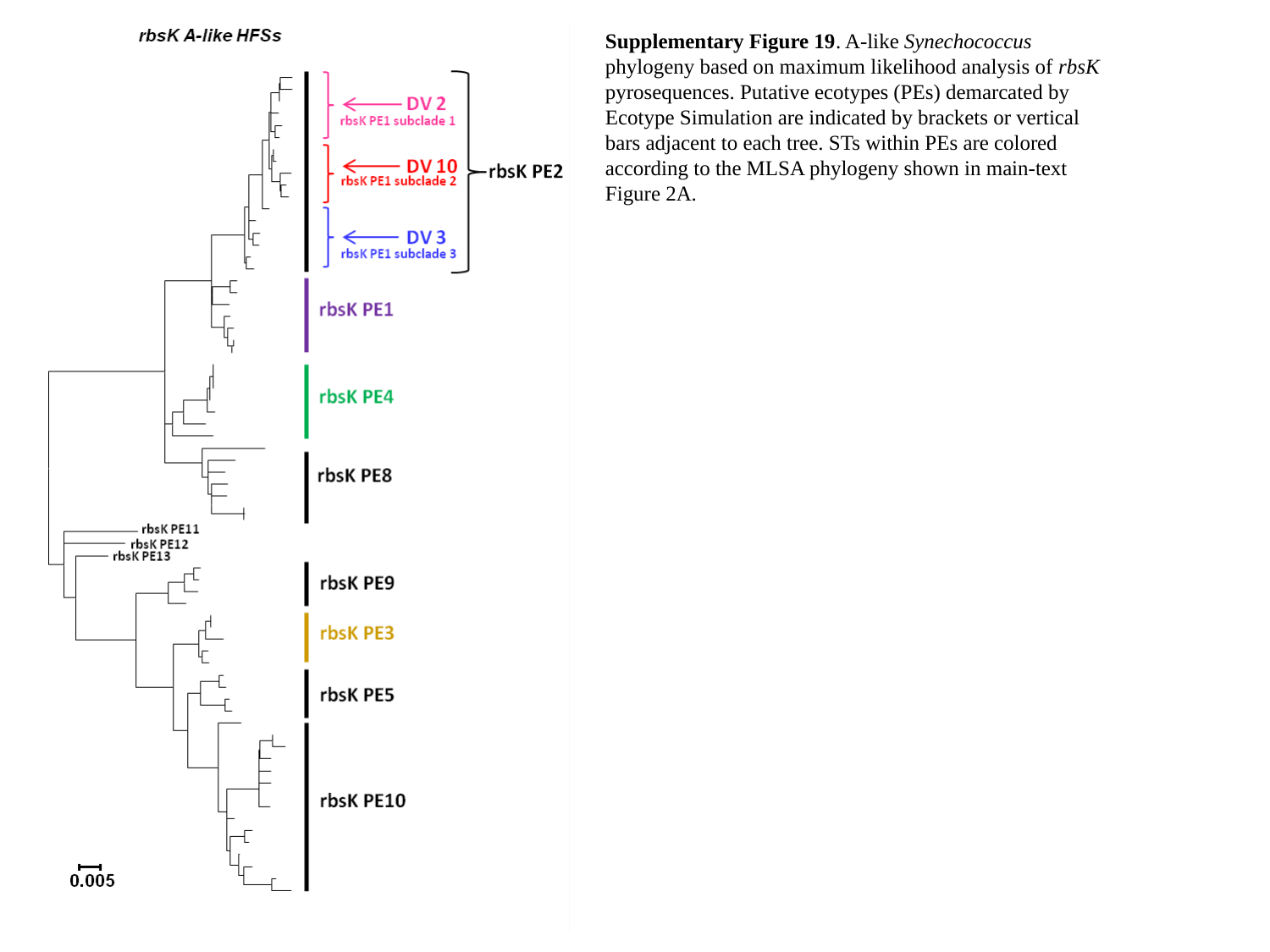

Supplementary Figure 19. A-like Synechococcus phylogeny based on maximum likelihood analysis of rbsK pyrosequences. Putative ecotypes (PEs) demarcated by Ecotype Simulation are indicated by brackets or vertical bars adjacent to each tree. STs within PEs are colored according to the MLSA phylogeny shown in main-text Figure 2A.

## Slide 21
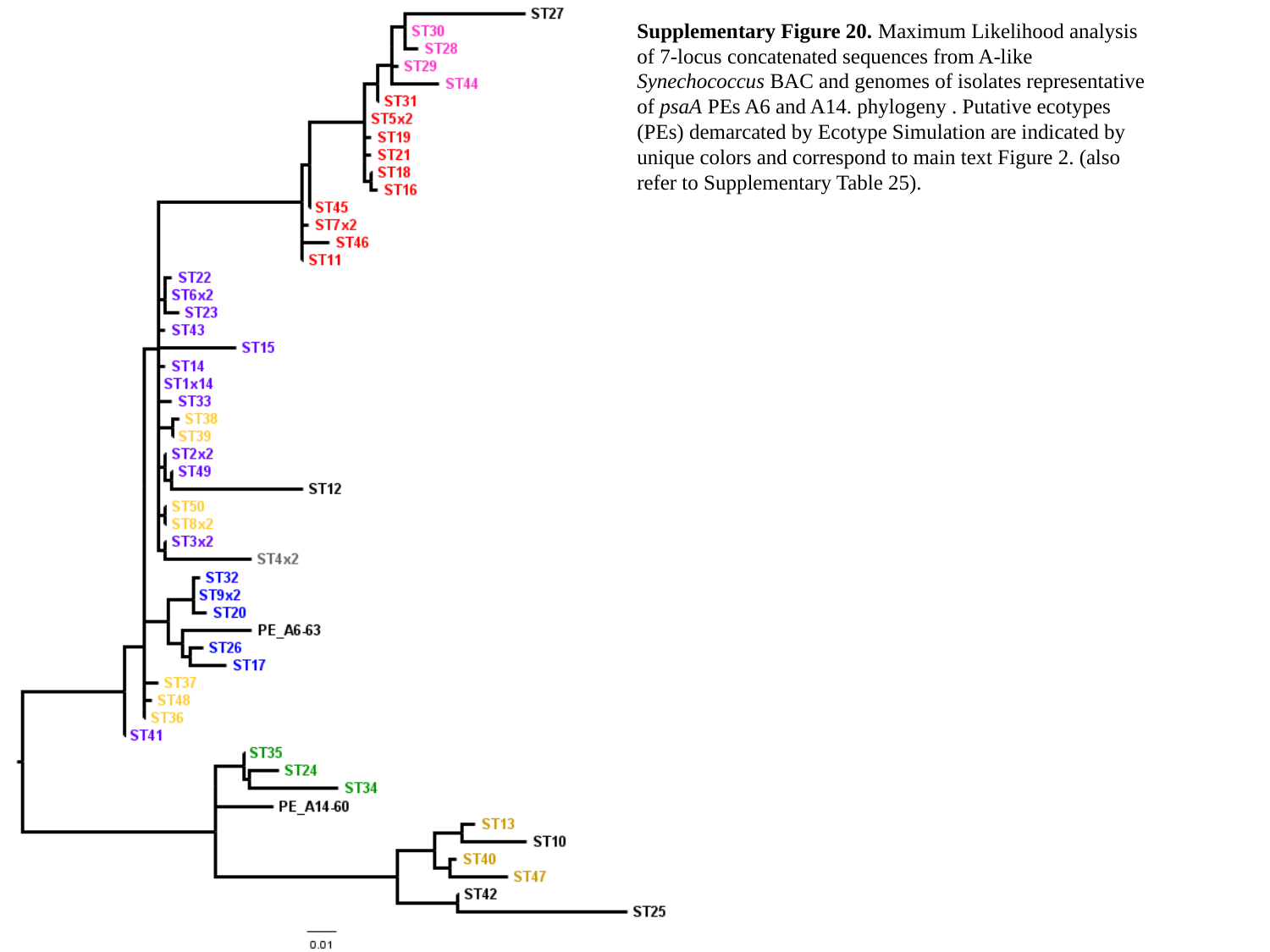

Supplementary Figure 20. Maximum Likelihood analysis of 7-locus concatenated sequences from A-like Synechococcus BAC and genomes of isolates representative of psaA PEs A6 and A14. phylogeny . Putative ecotypes (PEs) demarcated by Ecotype Simulation are indicated by unique colors and correspond to main text Figure 2. (also refer to Supplementary Table 25).

## Slide 22
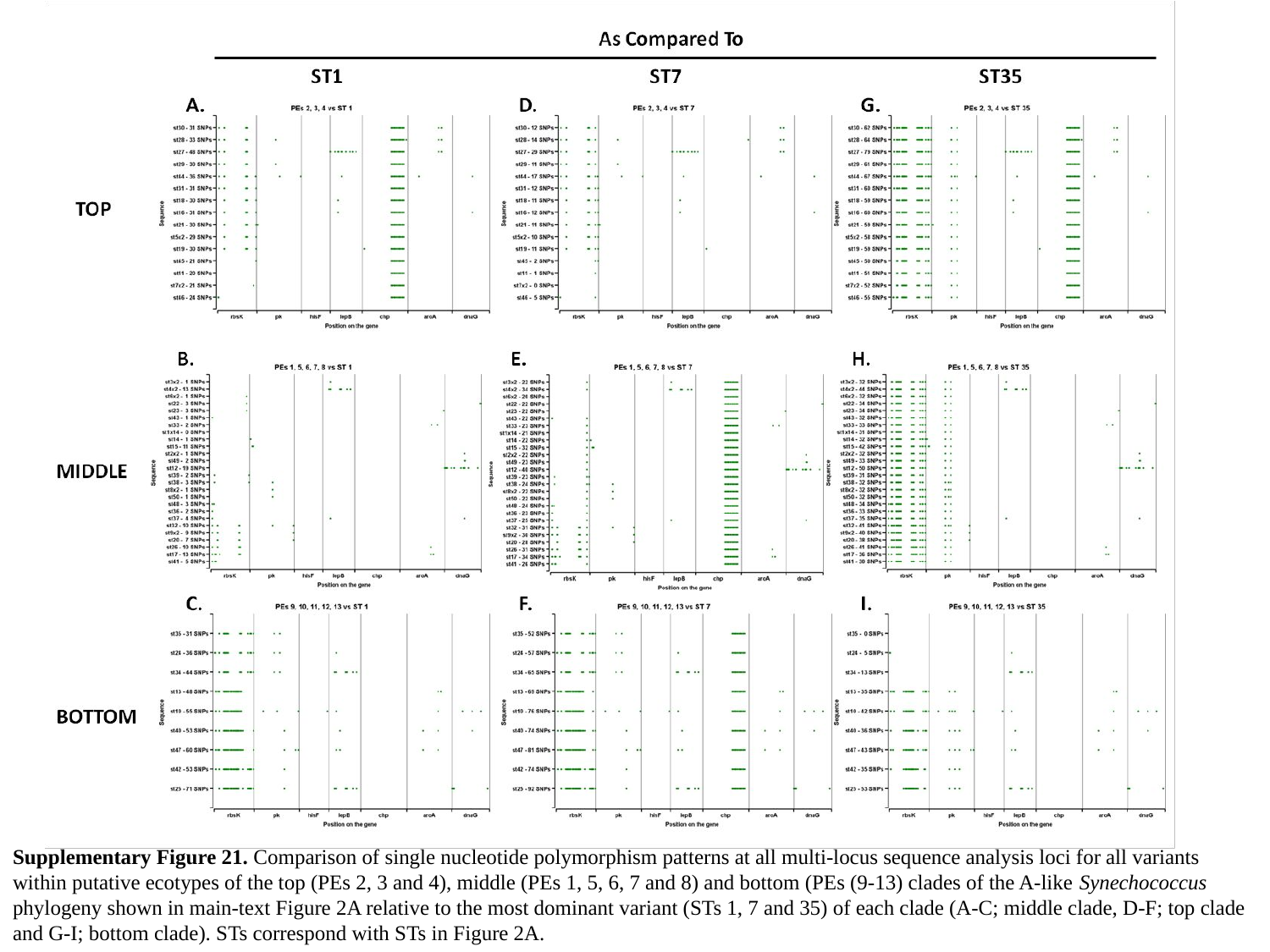

Supplementary Figure 21. Comparison of single nucleotide polymorphism patterns at all multi-locus sequence analysis loci for all variants within putative ecotypes of the top (PEs 2, 3 and 4), middle (PEs 1, 5, 6, 7 and 8) and bottom (PEs (9-13) clades of the A-like Synechococcus phylogeny shown in main-text Figure 2A relative to the most dominant variant (STs 1, 7 and 35) of each clade (A-C; middle clade, D-F; top clade and G-I; bottom clade). STs correspond with STs in Figure 2A.

## Slide 23
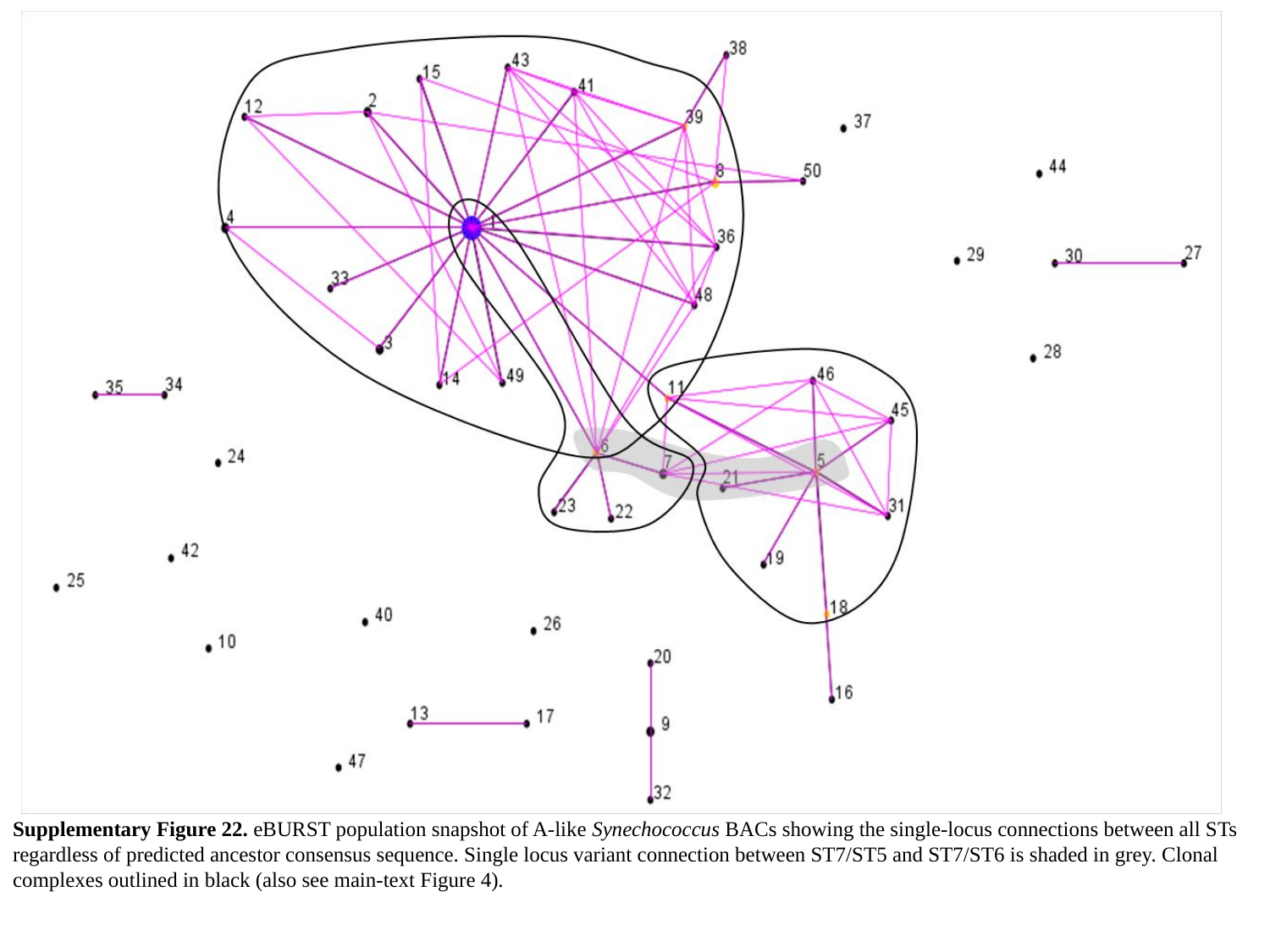

Supplementary Figure 22. eBURST population snapshot of A-like Synechococcus BACs showing the single-locus connections between all STs regardless of predicted ancestor consensus sequence. Single locus variant connection between ST7/ST5 and ST7/ST6 is shaded in grey. Clonal complexes outlined in black (also see main-text Figure 4).
