# Supplementary material for: Recombination Does Not Hinder Formation or Detection of Ecological Species of Synechococcus Inhabiting a Hot Spring Cyanobacterial Mat
Source: Front Microbiol. 2016 Jan 14;6:1540. doi: 10.3389/fmicb.2015.01540 (PMC4712262; doi:10.3389/fmicb.2015.01540)
Supplement: Supplementary file 3 [file DataSheet1.docx]

Supplementary Material Text

Recombination does not hinder formation or detection of ecological species of *Synechococcus* inhabiting a hot spring cyanobacterial mat

Melanie C. Melendrez^1*^, Eric Becraft^1^, Jason M. Wood^1^, Millie T. Olsen^1^, Donald A. Bryant^2^, John F. Heidelberg^3^, Doug B. Rusch^4^, Frederick M. Cohan^5^ and David M. Ward^1^

^1^ Department of Land Resources and Environmental Science, Montana State University, Bozeman, MT, USA

^2^ Department of Biochemistry and Molecular Biology, Pennsylvania State University, University Park, PA, USA

^3^ Department of Biological Sciences, College of Letters, Arts and Sciences, University of Southern California, Los Angeles, CA, USA

^4^ Informatics Group, J. Craig Venter Institute, Rockville, MD, USA

^5^ Department of Biology, Wesleyan University, Middletown, CT, USA

*** Correspondence:** Walter Reed Army Institute of Research, Viral Diseases Branch, 503 Robert Grant Ave. Silver Spring, MD 20910 USA. [mmelendrez@gmail.com](mailto:mmelendrez@gmail.com)

| **List of Acronyms**  *aroA* | 3-phospho-shikimate 1-carboxyvinyl-transferase |
| --- | --- |
| BAC | Bacterial artificial chromosome |
| CC | Clonal complex |
| *CHP* | Conserved hypothetical protein |
| *dnaG* | DNA primase |
| *hisF* | Imidazole-glycerol phosphate synthase, cyclase subunit |
| *lepB* | Signal peptidase I |
| MLSA | Multi-locus sequence analysis |
| *pcrA* | ATP-dependent DNA helicase |
| PE | Putative ecotype |
| *PK* | Protein kinase |
| *rbsK* | Ribokinase |
| SNP | Single nucleotide polymorphism |
| ST | Sequence type |

**SI Text Section I**: Selection of Loci for Multi-Locus Sequence Analysis. Construction of Bacterial Artificial Chromosome Libraries (BAC) and Screening of BAC Clones.

Loci for multi-locus sequence analysis were selected based on several criteria. The genomes of *Synechococcus* strains JA-2-3Aa and JA-2-3B´a(2-13), representative of the A and B´ lineages, respectively ([1](#_ENREF_1)), were screened to identify protein-encoding loci in the vicinity of the two 16S rRNA loci that might be useful for MLSA. Criteria included that they: (i) were present in one copy, (ii) were under neutral or purifying selection (d_N_/d_S_ <1; i.e., the sequence variation was not responsible for adaptations separating PEs), (iii) represented a range in divergence (0.1% to 1.8% for A-like and 0.99% to 3.5% for B´-like variants, respectively), (iv) exhibited a range of distances from the 16S rRNA locus (15-82 kb and 8-61 kb in the *Synechococcus* strain A and B´ genomes, respectively) and (v) were not adjacent to potential mobile elements (exception noted in SI Text Section III and Supplementary Figure 5). Characteristics of the loci selected are presented in Supplementary Table 1 and 2 and Supplementary Figure 1. Supplementary Tables 1 and 2 show loci selected along with their characteristics for *Synechococcus* strain A-like and B´-like BACs. Supplementary Figure 1 shows placement of the loci selected in relation to the nearest 16S rRNA locus of *Synechococcus* strain A (JA-3-3-Ab; Genbank Accession: CP000239.1) and strain B´ (JA-2-3b a (2-13); Genbank Accession: CP000240.1) genomes.

##

## Sample Collection and Preparation

Samples (approximately 1.5 cm length × 0.5 cm width × 0.5 cm thick) were collected from 60°C and 65°C sites in the mat of Mushroom Spring, Yellowstone National Park, WY (44.5386°N, 110.7979°W) on 2 October 2003 as previously described ([2](#_ENREF_2)). Samples were frozen on dry ice, transported to Montana State University and kept frozen at -80°C until analysis. These samples were replicates from the same collection used by Melendrez et al., Klatt et al., and Ward et al. ([2-4](#_ENREF_2)) The top 1 mm-thick green layers of three thawed mat samples from the same site were separated from the bottom layers using a razor blade, combined and homogenized with 2 ml of 10 mM Tris-HCl buffer, pH 7.5 using a Dounce tissue homogenizer. Aliquots (1 ml) of the mat homogenate were transferred into two 2 ml screw cap tubes and centrifuged at 14,000 rpm (Eppendorf, 5415C) for 5 min to pellet the cells. The supernatant was removed and the cells were resuspended in 1 ml of 10 mM Tris/EDTA buffer (TE: 10 mM Tris-HCl, pH 7.5 and 1 mM EDTA).

##

## BAC Library Construction

An 850 μl cell suspension from either 60°C or 65°C samples was transferred to a 2 ml tube, 20 μl of lysozyme (10 mg/ml) was added, and the mixture was incubated for 30 min at 37°C. The cell suspension was viewed under the microscope for the appearance of *Synechococcus* spheroplasts and then centrifuged at 14,000 rpm (Eppendorf, 5415C) for 5 min to pellet the spheroplasts and remaining cells. The supernatant was removed and the cells were frozen and sent to Amplicon Express (<http://www.genomex.com>) (Pullman, WA), where they were resuspended in 800 μl of TE buffer and 870 μl of molten 1% Seakem GTG Agarose was added. This suspension was aliquoted into 200-μl plug molds (BioRad) and plugs were solidified at 4°C for 10-15 min. For in-gel lysis, five plugs were incubated in a 50-ml Falcon tube (Eppendorf) containing 40 ml of ESP buffer (ESP: 0.5 M EDTA, pH 9-9.5; 1% Sarkosyl and 50 μg/ml proteinase K) and an additional 40 mg of crystalline proteinase K (final concentration of solution, 1 mg/ml proteinase K) overnight at 55°C with rotation in a hybridization incubator (Robbins Scientific). The buffer was discarded and replaced with fresh ESP buffer and 40 mg of crystalline proteinase K and the mixture was incubated an additional hour at 55°C with rotation. ESP Buffer was discarded and plugs were briefly washed with 10 mM TE Buffer. 40 ml of fresh TE buffer was added and plugs were incubated at room temperature for 1 hour with rotation. TE buffer was discarded and replaced and plugs were stored at 4°C overnight to stabilize high molecular weight (HMW) DNA.

The 60°C BAC library was constructed using a method adapted from Liles et al. and Tao et al. ([5](#_ENREF_5), [6](#_ENREF_6)).  HMW DNA was partially digested with HindIII and size-selected to target molecules on the order of 100 kbp in length, which were obtained using pulse-field gel electrophoresis (see below). Ligation of partially digested HMW DNA to the pECBAC1 vector was carried out in a tube with 100 μl of insert DNA (molar ratio 4:1 vector excess) and 5 units of T4 DNA ligase (GIBCO BRL) incubated at 16°C for 10 hrs. The resultant BAC clones were transformed into DH10B *Escherichia coli* cells (Invitrogen) and plated on LB agar (Fisher Scientific) with chloramphenicol (12.5 μg/ml), X-gal (40 μg/ml) and IPTG (0.4 mM). Clones were robotically picked with a Genetix QPIX (Genetix) into 792 384-well plates containing Luria Broth (LB) freezing media ([7](#_ENREF_7)). Plates were incubated for sixteen hours, replicated and then frozen at -80°C. The replicated copy was used as a source plate for nylon filters containing imprinted BAC clones that were sent to Montana State University for screening and processing (see below). The 65^o^C library was produced in the same way, except that the cloning vector was pCC1BAC and only 168 384-well plates were made.

To estimate insert sizes, 10 μl aliquots of BAC miniprep DNAs were digested with 5 U of Not I enzyme (New England Biolabs) for 3 hrs at 37°C. The digestion products were separated by pulsed-field gel electrophoresis (CHEF-DRIII system, BioRad) in a 1% agarose gel in 0.5x Tris/borate/EDTA (TBE) electrophoresis buffer (10x TBE: 890 mM Tris, 890 mM boric acid, 20 mM EDTA, pH 8.0). Insert sizes were compared to those of the Lambda Ladder PFG Marker (New England Biolabs). Electrophoresis was carried out for 18 hrs at 14°C with an initial switch time of 5 sec and a final switch time of 15 sec in a voltage gradient of 6 V/cm.

Oligonucleotide Hybridization Screening for Clones Containing *Synechococcus* A/B Lineage-specific 16S rRNA Genes

BAC clones imprinted and lysed on Hybond-N+ nylon membranes (Amersham Biosciences) prepared by Amplicon Express were probed with a ^32^P-radiolabeled *Synechococcus* A/B-lineage cluster probe (5´-ctgagacgcggtttttgg-3´) ([8](#_ENREF_8)), which was prepared by mixing 2.7 μl of the A/B-cluster probe (50 μM) with 4 μl water (Sigma), 1.3 μl T4 10X kinase buffer (Promega), 1.3 μl T4 polynucleotide kinase (Promega) and 4 μl ^32^P-ATP (Perkin Elmer). The mixture was incubated at 37°C for 20 min., denatured for 2 min. at 90°C and immediately placed on ice. 133 μl of water (Sigma) was added to bring the total volume to 146.3 μl.

Membranes were placed into hybridization tubes and rinsed with autoclaved double-distilled water, then with 5× sodium chloride/sodium citrate (SSC) buffer (20x SSC: 3M NaCl, 0.3M Na_3_citrate-2H_2_0, pH 7.0). 1.34 ml of pre-hybridization buffer [5× SSC, 5× Denhardt’s Solution (Fisher Scientific), 0.5% sodium dodecyl sulfate (SDS, Fisher Scientific)] was added to each tube and the tubes were placed into a hybridization oven (Robbins Scientific, model 400) for 30 min. with rotation at 51°C. After pre-hybridization, 36.6 μl of probe mixture was added and the probe and filter were hybridized overnight at 51°C with rotation.

To remove unreacted probe following hybridization, membranes were washed twice at low stringency (2× SSC, 0.1% SDS), twice at medium stringency (1× SCC, 0.1% SDS) and four times at high stringency (0.1× SSC, 0.1% SDS) (adapted from the Amersham Biosciences H-bond Nylon+ Membrane standard protocol). Membranes were kept wet, placed in saran wrap and exposed for approximately 30 h on a phosphoimaging cassette (Kodak). Positive clones were visualized using a phosphoimager (Phosphoimager) and the program ImageQuant Software (<http://www.imsupport.com>). Positive clones were transferred at Amplicon into 96-well plates containing 800 µl of LB (Fisher Scientific), grown overnight at 37°C and then 200 µl of autoclaved 80% glycerol was added to each well for preservation at -80°C. Plates were then frozen until PCR analysis using primers specific for the 16S rRNA internal transcribed spacer region of cyanobacteria (781cyF and L23cyR), as described in reference ([2](#_ENREF_2)).

# SI Text Section II: BAC-end Sequencing and Evidence of Genomic Inversion

The paired ends of all cyanobacterial BACs were sequenced at the J. Craig Venter Institute (JCVI: <http://www.jcvi.org>) using the Sanger sequencing method and T7 (5´-taatacgactcactatag|gg-3´) and M13R (5´-caggaaacagctatgac-3´) primers. Sequences have been submitted to Genbank (Accession Numbers: HQ187926-HQ187928, HQ187930-HQ187935, HQ187937- HQ187941, HQ187944, HQ187945, HQ187947-HQ187953, HQ187956-HQ187959, HQ187961-HQ187965, HQ187967-HQ187969, HQ187971-HQ187979, HQ187981-HQ187983, HQ187985-HQ187992, HQ187995 , HQ188133, HQ188138, HQ188143, HQ188150, HQ188158, HQ188160, HQ188164, HQ188167, HQ188172, HQ188173, HQ188176, HQ188177, HQ188179, HQ188181, HQ188183, HQ188186, HQ188191, HQ188193, HQ188195-HQ188198, HR575928-HR614199). These sequences were compared to reference genomes and metagenomic assemblies ([3](#_ENREF_3)) in BLAST analyses in order to assess their similarity to small- and medium-insert metagenomes (Supplementary Figure 2 and Supplementary Tables 3 and 4).

Paired-end sequences were mapped along the genomes of *Synechococcus* strains A and B´ (main text Figure 1A and Supplementary Figure 3). Both syntenous and non-syntenous clones jointly recruited by the same reference genome clones were observed. Sequences containing *Synechococcus* A-like or B´-like 16S rRNA sequences generally mapped to the vicinity of one (syntenous) or both (non-syntenous) of the 16S rRNA loci (main text Figure 1B and Supplementary Figure 3) with an exception in *Synechococcus* B´-like BACs. For *Synechococcus* B´-like BACs there appears to be a potential B´- or B-like subpopulation (highlighted by grey box in Supplementary Figure 3), in which the end sequences map to a different portion of the reference genome. There were relatively fewer syntenous clones in B´-like BACs than in A-like BACs. Many non-syntenous BACs seem to bridge between the genomic regions near the two 16S rRNA loci in the reference genomes (blue dots in main text Figure 1B and Supplementary Figure 3). Mate pair analysis revealed a difference between randomly sampled BACs and BACs containing *Synechococcus* A-like and B´-like 16S rRNA genes (Figure 3C main text and Supplementary Figure 3 and Supplementary Table 5). BACs containing A-like and B´-like *Synechococcus* 16S rRNA genes show a greater relative abundance of normal- and anti-normal long clones than was observed among randomly sampled BACs, suggested that these may be associated with genomic inversions ([9](#_ENREF_9)).

**SI Text Section III:** Locus Amplification and Distribution in *Synechococcus* A-like and B´-like BACs

Loci were amplified using A-like or B´-like *Synechococcus* lineage-specific primers for each protein-encoding locus (see main-text Materials and Methods) and the distribution of BACs positive for specific loci is shown in Supplementary Tables 6 and 7. The number of BACs positive for any given locus as a function of distance from the 16S rRNA gene is shown in Supplementary Figure 4, which shows that the number of BACs positive for various loci decreased as the distance from the 16S rRNA locus in the reference genome increased. Supplementary Table 7 illustrates the presence of many B´-like BACs containing only loci upstream of the 16S rRNA locus (*aroA, rbsK* and *pcrA*). When the region was examined further a site-specific recombinase was found, suggesting that genes on either side of the recombinase might have been relocated to a different part of the genome (Supplementary Figure 5).

Within the A-like and B´-like lineages, many genes near the two 16S rRNA loci in these organisms have apparently been relocated on the genome as a consequence of genomic inversions and other mechanisms, such as by transposition. It is unlikely these genes were ‘lost’ from the genome as they represent loci that actively code for enzymes necessary for essential pathways of biosynthesis, photosynthesis, iron processing, and DNA strand separation and/or management (Supplementary Tables 1 and 2). There were two possible exceptions: in the A-like *Synechococcus* population, to increase resolution, both a ‘general’ protein kinase (*PK* [locus tag CYA_2262]) and conserved hypothetical protein (*CHP* [locus tag CYA_2291]) were selected for analysis. It was assumed that the likelihood of ‘losing’ these genes was low as protein kinases are involved in protein modification and the hypothetical protein selected was ‘conserved’ suggesting that it was a protein that was not likely to be lost from the genome. In addition, neither of these loci was located near mobile elements (Supplementary Figure 1) that might have promoted gene loss through recombination or transposition.

# SI Text Section IV: Comparison of Diversity Sampled Using PCR and BAC Cloning Methods

To determine if BAC cloning methods yielded sequences representative of the natural diversity, BAC clone sequences were combined with sequences from an *rbsK* gene PCR library and analyzed phylogenetically (see main text Materials and Methods, SI Text Section XIII and reference ([2](#_ENREF_2))). Despite lower coverage, BAC clones were found to sample most of the PEs in the *rbsK* phylogeny that were sampled by PCR-generated small-insert clones for both *Synechococcus* A- and B´-like BACs. (Supplementary Figures 6 and 7). This suggested that BAC cloning methods were sampling the same diversity as was sampled by PCR cloning methods.

**SI Text Section V:** Sequence Type/Allelic Profile Generation for 7-locus MLSA Study of A-like and 4-locus Study of B´-like *Synechococcus* BACs.

Allelic profiles were constructed from sequence alignments with identical sequences at each locus being assigned to the same allele type for that locus. Allele types for all loci were combined for each BAC into an allelic profile and each unique allelic profile was designated as a unique sequence type (ST). Allelic profiles and sequence type designations are shown in Supplementary Tables 8 and 9).

**SI Text Section VI:** Recombination and Linkage within and between A-like and B´-like *Synechococcus* BACs

Within-lineage recombination

As mentioned in the main text, recombination within the A-like and B´like lineages was done using RDP4, Clonal Frame and manual SNP analysis (see main-text Table 3). RDP4 detects recombination using 7 different methods in addition to phylogenetic incongruency (see main-text Materials and Methods, SI Text Section XIII and reference ([10](#_ENREF_10))). All recombinants identified by 3 or more methods at a p-value < 0.05 within RDP4 were further analyzed to determine the clonal complex and PE to which the recombinant belonged. The positions of potential breakpoints in the gene for that recombinant, and the number of single nucleotide polymorphisms (SNPs) between the recombinant gene and the dominant variant sequence type (within the PE or clonal complex) were also determined (Supplementary Tables 10 and 11). Significance values for RDP4 analysis are presented in Supplementary Tables 12 and 13. Breakpoint positions were identified within the *rbsK* locus in both *Synechococcus* A-like and B´-like recombinant BACs with two breakpoint possibilities identified in A-like BAC recombinants (156-358 and 231-518 depending on recombinant) and two potential breakpoints identified in B´-like BAC recombinants (568-928 and 636-1085) (Supplementary Tables 10 and 11). Phylogenies constructed from sequences on either side of breakpoint pairs in the recombinant *rbsK* sequence for A-like and B´-like *Synechococcus* BACs were found to be incongruent (Supplementary Figures 8 and 9). Phylogenies constructed from *rbsK* sequences amplified from BACs were also incongruent with the concatenated multi-locus phylogenies (main text Figure 2, Supplementary Figure 10).

Due to Sanger sequencing length limitations (many targeted genes were >800 bps), trimming due to low quality ends, and primer design limitations, only a portion of each gene was analyzed in this study (27-67% of the total length for the A *Synechococcus* genes and 27-58% of the total length for the B´ *Synechococcus* genes; also see Supplementary Tables 1 and 2). Therefore, it is important to note that recombination may be underestimated and breakpoint ends were not always identified, suggesting that the recombined segment (within the gene) was larger than the portion of the gene that was sequenced. Those instances where no ‘start’ or ‘end’ breakpoint could be defined are noted in the footnotes of Supplementary Tables 10 and 11. Methods for Clonal Frame and SNP analyses were provided in the main text.

These methods were used in the analysis of the primary A-lineage (MLSA7) and B´-lineage (MLSA 4) databases, which contained nearly equal numbers of BACs (71-72), but different numbers of loci. Additionally, we analyzed the two MLSA 5 databases, which contained different numbers of BACs (49 and 145), but the same number of loci. Since different methods detected different recombination events, and the number of BACs and the number of loci affected the results, we reported a range of unique recombination events detected by all methods. Events are recorded in Supplementary Table 10 and main-text Figure 2A for *Synechococcus* A-like BACs and Supplementary Table 11 and Supplementary Figure 10 for *Synechococcus* B´-like BACs. A summary of the events across concatenated datasets is described in Table 3 of the main text. For the *Synechococcus* A-like BAC datasets the number of unique events recorded across all three methods ranged from 8 to 35 illustrating how study design and scale of analysis (number of sequences, number of genes and sequence length of those genes) can affect the number of recombination events found. For the *Synechococcus* B´-like BAC dataset, 24 unique events were found using all methods. Taking into account the number of BACs in the analysis, this amounts to a within-lineage ‘exchange’ of 5.5% and 33.3% for *Synechococcus* A- and B´-like populations respectively.

Recombination between the A and B´ lineages

RDP4 was also used to investigate recombination between lineages, but could only be performed on overlapping sequences of genes common to the analysis of both lineages (i.e., 16S rRNA, and *rbsK*). Alignments containing equivalent numbers of both A- and B´-like BACs for these loci were constructed using MUSCLE ([11](#_ENREF_11)) and trimmed so that only the overlapping nucleotide region common to A- and B´-like BACs was analyzed for both separate and combined datasets. These datasets were unique from MLSA datasets. Alignments were analyzed using RDP4 to compare the number of recombination events (signals) detected within each dataset. Both number of sequences and length of sequence analyzed were affected by combining sequence data from both A- and B´-like BACs; however, enough sequence information was present to conduct RDP4 analysis. Results are presented in Supplementary Table 14. For the 16S rRNA gene no events were detected in any dataset, most likely due to the small length of overlapping sequence analyzed (Supplementary Table 14; 505 nt; 38% of the gene). For the *rbsK* datasets 8 total recombination signals were detected, only 3 of which were specifically between clones from A-like and B´-like BACs. All 3 recombinants were determined to be *Synechococcus* B´-like BACs by 16S rRNA designation, however they appeared to have portions of their *rbsK* genes that were significantly more similar to A-like clones (Supplementary Table 14). This suggested no instances of cross-lineage recombination for the A-like BACs and 3 instances of cross-lineage recombination for B´-like BACs. Given the number of sequences in the analysis (n=123) this amounts to 0% and 2.4% cross-lineage recombination for *Synechococcus* A- and B´-like BACs respectively.

BLAST analysis was used as a separate means of observing evidence of recombination between the A- and B´-lineages. During concatenated phylogeny construction it was found that several STs had visually longer branch lengths. The genes from these STs were BLASTed against isolate genomes to confirm that all genes within the concatenation belonged to the same lineage as dictated by the 16S rRNA gene for that clone/ST. For the *Synechococcus* B´-like BACs STs 13, 15, 43, 46 and 47 were assessed (Supplementary Figure 10). Clone M60B700J15 (ST15) was found to belong to the B´-like population due to the 100% nucleotide identity (ntid) to the 16S rRNA locus, as well as 97-100% ntid of the other genes analyzed (*pcrA* and *aroA*) compared to homologs in the reference genome. The *rbsK* gene on this clone, however, was 93% identical at the nucleotide level to the *Synechococcus* strain A reference genome and 88% identical to the *Synechococcus* strain B´ reference genomes. We presumed that this clone was from the B´-like population and had acquired an A-like *rbsK* gene via recombination. A second clone, also from the B´-like BACs, M60B579B21—ST46, was determined to belong to the B´-like *Synechococcus* population by 16S rRNA homology however had an *rbsK* gene that was nearly equal in nucleotide identity to both reference genome homologs (91% ntid to the *Synechococcus*  isolate A genome and 92% ntid to the *Synechococcus* isolate B´ genome). This clone was also determined to belong to the B´-like population, however it is unclear if the *rbsK* locus was acquired via recombination or if it is just a highly divergent *rbsK* locus.

For *Synechococcus* A-like BACs STs 4, 12, 27, 34 and 25 were examined based on the MLSA 7-locus concatenated phylogeny (main-text Figure 2) and additionaly STs 44 and 65 were examined based on the MLSA 5-locus concatenated phylogeny of 145 sequences (Supplementary Figure 16). Only one clone was found, (M60B573N02—ST65), which was determined to belong to the A-like *Synechococcus* population by 16S rRNA homology, however, contained an *rbsK* gene that was more similar to the *Synechococcus* strain B´ genome homolog (93% ntid) than to the *Synechococcus* strain A genome homolog (89% ntid). All other protein-encoding loci analyzed for this clone were 98-100% identical to the *Synechococcus* strain A reference genome. This clone was therefore presumed to be a member of the A-like population which acquired a portion of its *rbsK* locus via recombination with the B´-like population. For the studies where recombinants were identified from the concatenated phylogenies (via long branch lengths), there were a total of 145 A-like and 72 B´-like sequences for which data for multiple genes was analyzed. As mentioned above, 2 clones were determined to be B´-like and 1 A-like by 16S rRNA locus determination, but contained *rbsK* genes more similar to the other genome. This amounts to 0.69% of A-like and 2.8% of B´-like BACs that exhibited recombination across the A/B´ lineages. This analysis was based on visual examination of the concatenated phylogenetic trees and may underestimate the percent of recombination across lineages.

Linkage disequilibrium

Linkage was assessed using the standardized index of association (I_A_^S^), which measures the degree of association between alleles at different loci based on the variance in genetic distance between genotypes, was determined with LIAN version 3.5 ([12](#_ENREF_12)). I_A_^S^ values were calculated from allelic profiles generated from analysis of MLSA data for *Synechococcus* A-like (7 loci) and B´-like (4 loci) populations. The significance of I_A_^S^ was determined by comparison to the null hypothesis of free recombination simulated by 10,000 randomized reshufflings of alleles for each locus among individuals.

This analysis allowed for the independent assortment of alleles in given sequence types independent of phylogeny or nucleotide similarity scores. Concatenated sequences of all loci for both *Synechococcus* A-like and B´-like populations had a standardized index of association value (I_A_^S^; ([12](#_ENREF_12))) significantly different from zero, which suggested that recombination was not frequent enough to allow linkage equilibrium (Supplementary Table 15). Testing different combinations of loci at different degrees of separation still resulted in linkage disequilibrium (I_A_^S^ significantly different from zero, P < 0.05). The mean allelic diversity (i.e., proportion of polymorphic sites) was also determined to be lower for *Synechococcus* A-like BACs (0.389-0.63 depending on the alignment used) than for B´-like BACs (0.859) (Supplementary Table 15).

# SI Text Section VII: Analysis of 4 Concatenated Loci in *Synechococcus* B´-like BACs

##

## Ecotype Simulation Analysis

For the 4-locus analysis of *Synechococcus* B´-like BACs, Ecotype Simulation predicted 29 B´-like PEs from concatenated MLSA sequence data and 8 to 22 B´-like PEs from individual loci, depending on the locus analyzed (Supplementary Figures 10 and Supplementary Table 16). The greatest number of PEs was predicted from the *rbsK* gene, which had the greatest average evolutionary divergence (AED) as was seen with A-like *Synechococcus* BACs (main text Table 4). Concatenated PE clade sizes ranged from 1-12 sequences. Six of the 29 PE clades for *Synechococcus* B´-like BACs contained a dominant variant, which was identical at all loci surrounded by singleton variants, except in the case of PE27, which only contained identical sequences (number of variants belonging to dominant STs are indicated in parentheses next to ST designation in Supplementary Figure 10). There were no cases of subdominant PE variants in the B´-like *Synechococcus* BAC analysis. Supplementary Table 17 shows how STs and dominant variants were distributed among PEs for this analysis.

The single-locus analysis *rbsK* phylogeny for B΄-like *Synechococcus* BACs was also incongruent with concatenated MLSA phylogeny, as seen in the A-like *Synechococcus* BAC analysis (compare Supplementary Figure 10 and main text Figure 2). There were cases for which several singleton PEs in *rbsK* analysis remained singleton PEs in MLSA analysis. There were 5 cases of complete conservation of the PE clade comparing results from *rbsK* and MLSA analyses (*rbsK*-PE6 and MLSA-PE5, *rbsK-*PE7 and MLSA-PE16, *rbsK*-PE8 and MLSA-PE18, *rbsK*-PE22 and MLSA-PE25, and *rbsK*-PE19 and MLSA-PE27). Similar to the analysis of A-like BACs, concatenated MLSA Ecotype Simulation analysis also split variants that were grouped by *rbsK* single-locus analysis. For instance, *rbsK* PEB´ 12 was split into MLSA4 PEs B´ 8, 9, 10, 11 and 15.

## MLSA-eBURST Analysis of B´-like *Synechococcus* BACs

eBURST analysis was conducted on the same BACs used in the 4-locus analysis (Supplementary Figure 11 and Supplementary Table 18; allelic profiles in Supplementary Table 9). eBURST predicted 3 B´-like clonal complexes (CCs) and 2 additional CCs were identified if the criteria were relaxed to allow consensus groups with only 2 single-locus variants. CC B4-I contained a consensus group of 10 sequences (ST1) and 6 single locus variants, all of which were singleton STs. ST6 (with 2 sequences) was the consensus group for clonal complex B4-II, which included 6 single locus variants. ST5 (with 2 sequences) was the consensus group for clonal complex B4-III, which contained 5 single locus variants. No variants were shared between any of the clonal complexes and if the criterion were relaxed to allow for double-locus variants, no additional clonal complexes were observed

Correspondence between PEs and CCs in the B´-like *Synechococcus* population was generally better than for A-like *Synechococcus* BACs. This may be due to the greater average evolutionary divergence found within the B´-like *Synechococcus* BACs and metagenomic homologs (Supplementary Tables 2 and 16) versus A-like *Synechococcus* BACs and metagenomic homologs (main text Table 4 and Supplementary Table 1), which may facilitate the correspondence between PEs and clonal complexes as organisms diverge over time. In the B´ analysis, none of the 5 CCs overlapped as seen in the A-like population and 4 of 5 circled CCs in Supplementary Figure 11 encompassed all the variants in the colored PE. Similar to the A-like *Synechococcus* BAC analysis, in *Synechococcus* B´-like BAC eBURST analysis, not all variants within a defined CC were found in the same PE (e.g. CC B4-I and PEs 1 and 2 in Supplementary Figure 11). PEs 6, 16, 18 and 25 nearly corresponded with the identified clonal complexes with the exception of singleton STs 19, 24 and 30. Also PEs 2, 5, and 13 were not observed to belong to any clonal complex(es) illustrating that, although there seems to be better correspondence, it is imperfect in B´-like *Synechococcus* BACs.

## Ecotype Simulation PEs and eBURST Clonal Complex SNP Patterns

Supplementary Figure 12 panel A shows SNP patterns observed with variants grouped by Ecotype Simulation into PE-B´1 and B´2 and by eBURST into CC B´4-I around the common dominant variant ST1. STs 7, 8 and 12 were found in the same PE and CC while STs 9, 10 and 11 were only grouped with CC B´4-I presumably because of the large number of SNPs. Supplementary Figure 12 panel B shows variants that group with PE-B´6 and B´7 and by eBURST into CC B´4-II. There was near correspondence between the PE and CC with the exception of ST24 which contained 14 SNPs placing it as a singleton PE in Ecotype Simulation analysis. As with the previous CC B´4-II/PE-B6 comparison, CC B´4-III and PE-B´16 nearly corresponded with the exception of ST30 which differed from dominant variant ST5 by 14 SNPs (Supplementary Figure 12 panel C).

##

## Clade-Specific SNP Patterns

STs in the PEs of the three main lineages in the MLSA concatenated phylogeny (top: PEs 1, 2, 4-7, 14-16; middle: PEs 8, 10, 11- 13, 17 and 18; and bottom: PEs 3, 19, 20-29; Supplementary Figures 10 and 14) were compared to STs representative of each clade (STs 1, 2 and 4) (Supplementary Figures 10 and 13). SNP analysis of the *Synechococcus* B´-like BACs showed that 2-3 recombination events were associated with some divergences despite there being only 4 loci in the B´-like phylogeny (Supplementary Figure 13). Recombination events inferred from SNP analysis and Clonal Frame analysis (see main text for methods) are mapped onto the phylogeny shown in Supplementary Figure 10 (open and closed stars). Four unique events were detected by RDP4 analysis (mostly in the *rbsK* locus), one of which was also found using Clonal Frame (Supplementary Table 11). Clonal Frame and SNP analysis detected an additional 25 recent events (recorded in Supplementary Table 11; open/closed circles on terminal branches leading to STs in Supplementary Figure 10) and 16 historic events (open/closed circles on internal branches Supplementary Figure 10).

**SI Text Section VIII:** Additional Ecotype Simulation Analyses of 7 loci from A-like *Synechococcus* BACs and Habitat Heterogeneity of Putative Ecotypes

Additional Ecotype Simulation Analyses of 7 loci from A-like *Synechococcus* BACs

*PK, aroA* and *hisF* loci single locus Ecotype Simulation analyses are shown in Supplementary Figure 14. These loci exhibited low resolution resulting in Ecotype Simulation predicting 2-3 putative ecotypes (PEs) each.

Habitat Heterogeneity of Putative Ecotypes

An additional analysis was performed in order to test ecological heterogeneity. It involved analysis of a nearly equal proportion of BACs obtained from 60°C and 65°C samples. Allelic profiles were constructed from 5-locus sequence alignments (49 sequences) using the approach described in SI Text Section V and results are presented in Supplementary Table 19 with an Ecotype Simulation-demarcated phylogeny shown in Supplementary Figure 15. A second and separate phylogenetic and Ecotype Simulation analysis was done to determine if more temperature-specific ecotypes could be visualized in the A-like *Synechococcus* BAC sequence dataset from 60°C and 65°C if all available sequences were used (24 65°C sequences and 121 60°C sequences; Supplementary Figure 16; Supplementary Table 20). **[Note that the STs described in these tables are different from and cannot be directly compared to STs from other analyses, such as those in Supplementary Tables 8 and 9**.] In the analysis of 49 sequences, 13 PEs were demarcated on the concatenated phylogeny and between 1 and 9 PEs were demarcated for single gene phylogenies (Supplementary Figure 15 and Supplementary Table 21). Three of the 13 PEs observed in this MLSA were temperature specific (PEs annotated with an asterisk in Supplementary Figure 15). In the larger analysis, a total of 19 PEs were demarcated, 5 of which were temperature-specific (Supplementary Figure 16). A Fisher´s exact contingency test suggested significant evidence of heterogeneity in habitat associations across all ecotypes demarcated by Ecotype Simulation (*p*-value <0.001) regardless of size of dataset.

Overlay of PE demarcations onto the eBURST population snapshots for these datasets showed that 1 of 3 CCs in the smaller dataset of 49 sequences corresponded to a PE predicted by Ecotype Simulation analysis (Supplementary Figure 17 and Supplementary Table 22). Distributions of the variants (dominant/subdominant variants) between PEs and CCs in the small dataset (49 sequences) are shown in Supplementary Table 24. In the larger dataset (145 sequences), 7 of 9 CCs (Supplementary Figure 18) contained STs that were from >1 PE. Overlay of sample specificity onto the eBURST population snapshots for each dataset showed no evidence of sample/temperature-specific CCs with the exception of CC 1 in the larger dataset (Supplementary Figure 18), which was low-temperature specific. However, this result could be a consequence of the limited number of sequences available from the 65°C sample, which might bias the results in favor of 60°C. A Fisher’s exact test showed no significant clustering of CCs by temperature for either dataset, *p*-value = 0.943 (small dataset; Supplementary Figure 17), *p*-value = 0.734 (large dataset; Supplementary Figure 18).

**SI Text Section IX:** Additional evidence of ecological distinction of MLSA PEs.

Pyrosequencing analysis of the *rbsK* locus (Supplementary Figure 19) resulted in a phylogeny that closely resembled the *rbsK* phylogeny sampled in BACs (main text Figure 2B).

#

# SI Text Section X: Description of genome sequence for *Synechococcus* strain 63AY4M2

We report here the genome sequence of *Synechococcus* strain 63AY4M2. The isolate was obtained from a 63°C sample of the microbial mat in the effluent channel of Mushroom Spring using methods described in Nowack et al. ([13](#_ENREF_13)). The purity of the strain was ascertained by pyrosequencing analysis of *psaA* sequences, which demonstrated that 86.6% of 2027 sequences were identical to, and the remainder 1-2 randomly distributed SNPs different from, the *psaA* sequence used to demarcate *psaA* PE A6 as described in Becraft et al. ([14](#_ENREF_14)). The genome sequence is 3.09 Mbp, and is composed of 3 scaffolds, the largest of which is 3,083,974 bp long. There are 2636 CDS, 45 tRNAs, and 1 rRNA operon predicted from the assembly. The sequence data have been deposited at the DDBJ/EMBL/GenBank under the accession PRJNA210218 (Supplementary Table 25).

*Synechococcus* isolate 63AY4M2 is closely related to the other A-lineage low-light-adapted strains investigated by Olsen et al. ([15](#_ENREF_15)) in 16S rRNA sequence (0-2 SNP differences between strains in 1420 bp), average nucleotide identity (Supplementary Table 25) and gene content. It shares the *apcA*/*apcD*/*isiX* gene cassette hypothesized to be associated with low-light adaptation. It also shares other genes with the low-light-adapted PE A4 and A14 isolates, including those coding for *feoAB*, the paralogous nitrate reductase gene, and a sugar (possibly maltose/maltodextrin) ABC transport cluster. It has a beta-carotene ketolase gene as found in the PE A14 isolate (and also in *Synechococcus* JA-2-3B´a(2-13)), and a urea-carboxylase gene cluster, which is found in the PE A1-MS, PE A14, and PE A4 isolates. Isolate placement among *Synechococcus* A-like BAC STs can be seen in Supplementary Figure 20.

**SI Text Section XI:** Additional SNP analysis of 7 Concatenated Loci of A-like *Synechococcus* BACs

Given the evidence of potential ecotype-specific SNP patterning detected in our dataset and the probability that recombination was pulling variants away from their ‘true’ ecotype, we extended the analysis of SNP variants within the *Synechococcus* A-like BACs (Supplementary Figure 21). To investigate this, all STs in the PEs of the three main lineages in the MLSA concatenated phylogeny (top: MLSA PEs 2, 3 and 4; middle: MLSA PEs 1, 5, 6, 7 and 8; and bottom: MLSA PEs 9-13 in main text Figure 2A) were compared to STs representative of each of the three clades (STs 1, 7 and 35) (Supplementary Figure 21). Clear evidence of clade-specific SNP patterns was observed; for instance, the 20-nt SNP pattern in *CHP* was found to be representative of all STs in the top clade (Supplementary Figure 21 panels A and G) when compared to ST1 (from the middle clade) or ST35 (from the bottom clade). Within the top clade, differences in *rbsK* SNP patterns suggested that recombination events at this locus were associated with the divergence of members of MLSA PEs 2, 3 and some members of MLSA PE4 (Supplementary Figure 21 panel D). A unique set of SNPs in the *lepB* locus appears to be associated with the separate demarcation of ST27 as singleton MLSA PE2 from the STs comprising MLSA PE3 (Supplementary Figure 21 panels A, D, G).

#

# SI Text Section XII: eBURST ‘Ancestors’ and Single-locus Variants

As discussed in the main text, in addition to predicting single locus variants belonging to consensus groups, the eBURST algorithm also predicts an ‘ancestor’ which usually enucleates a clonal complex. This means that, in reality, a variant can be a single-locus variant of multiple STs. The eBURST population snapshot shown in Supplementary Figure 22 illustrates this point, where all potential single-locus connections are highlighted in pink lines. Note that many of the connections cross clonal complex demarcations (outlined in black in Supplementary Figure 22) especially in the shaded area which includes STs 5, 6, 7 and 21. Both STs 5 and 6 were predicted as ancestors with their own sets of single-locus variants. Because ST7 was predicted to be a single-locus variant of the ‘ancestor’ ST6, it is grouped with ST6 despite also being a single-locus variant of ST5. Additionally, although an ST may be comprised of multiple identical variants, this does not affect whether it is predicted to be an ancestor or not. STs 6 and 7 are both comprised of two variants; however, given the allelic variation within the entire population, eBURST predicted ST6 to be the ‘ancestor’ rather than ST7.

# Supplementary Figures and Tables

**Supplementary Figures (captions also included with Figures in Supplementary Materials PPTX presentation file)**

**Supplementary Figure 1.** Genome region views of MLSA loci. (A) *Synechococcus* strain A, coordinates 2,224,764—2,339,273 and (B) *Synechococcus* strain B´, coordinates 1,359,388—1,535,948. Loci used in this study are highlighted in blue. Open reading frames annotated as transposons, resolvases, recombinases and mobile elements are highlighted with red stars. Red vertical bars show presence of CRISPR arrays. (Adapted from JGI genome region viewer, [**http://imgweb.jgi-psf.org/cgi-bin/w/main.cgi**](http://imgweb.jgi-psf.org/cgi-bin/w/main.cgi), ([16](#_ENREF_16), [17](#_ENREF_17))).

**Supplementary Figure 2.** BLASTN-based recruitment of metagenomic BAC end sequences by metagenomic assembly clusters and reference genomes.(A) 60^o^C and 65^o^C BAC sequences combined recruited by metagenomic assembly clusters observed by Klatt et al. ([3](#_ENREF_3)). (B) 60^o^C BACs and (C) 65^o^C BACs recruited by genomes of 20 microorganisms of possible relevance to these mats. Clusters (C): 1, A/B-lineage *Synechococcus* spp.; 2, *Roseiflexus* spp.; 3, *Chloroflexus* spp.; 4, *Candidatus* Cab. thermophilum-like organisms; 5, *Chlorobiales*-like organisms; 6, *Anaerolineae*-like organisms; 7 and 8, unknown possibly heterotrophic populations. Reference genomes: SA, *Synechococcus* strain A; SB´, *Synechococcus* strain B´; Telo, *Thermosynechococcus* *elongatus*; Ros, *Roseiflexus* sp. RS-1; C396, *Chloroflexus* sp. 396-1; Cthe, *Candidatus Chloracidobacterium thermophilum*; Ctha, *Chloroherpeton thalassium*; Tros *Thermomicrobium roseum*; Tthe, *Thermus thermophilus*; Haur, *Herpetosiphon aurantiacus*; Acid, *Acidobacterium* sp.; Tpse, *Thermoanaerobacter pseudoethanolicus*; Chyd, *Carboxydothermus hydrogenoformans*; Bvul, *Bacteroides vulgatus*; Tyel, *Thermodesulfovibrio yellowstonii*; Tcom, *Thermodesulfobacterium commune*; Rfer *Rhodoferax ferrireducens*; Mthe, *Methanothermobacter thermoautotrophicum*; Aaeo, *Aquifex aeolicus*; and Tneu, *Thermoproteus neutrophilus*. Shading indicates % nt identity of sequences within bins (details regarding selection of genomes are in Supplementary Table 3 and reference ([3](#_ENREF_3))).

**Supplementary Figure 3.** Coverage of (A) random (B) 16S rRNA *Synechococcus* B´-like BACs and (C) distributions and percent nucleotide identity of jointly recruited, normal-long, non-syntenous sequences in 60^o^C BAC clones containing *Synechococcus* B´-lineage 16S rRNA genes relative to the position along the *Synechococcus* strain B´ genome. Shaded box indicates a potential B´-like or B-like subpopulation (see main text). Lines connect end sequences of the same clone.

**Supplementary Figure 4.** Number of positive BACs per locus as a function of their separation from the 16S rRNA locus. Upstream is indicated by negative numbers, downstream indicated by positive numbers**.**

**Supplementary Figure 5.** Genome view of site-specific recombinase in *Synechococcus* strain B´. Adapted from JGI genome region viewer, <http://imgweb.jgi-psf.org/cgi-bin/w/main.cgi>, ([16](#_ENREF_16), [17](#_ENREF_17)).

**Supplementary Figure 6.** Bootstrapped, neighbor-joining phylogenetic tree for A-like *Synechococcus* *rbsK* sequences obtained from the Mushroom Spring 60^o^C (light blue and dark blue) and 65^o^C (red and pink) mat samples using either PCR- (pink and light blue) or BAC-based (dark blue and red) cloning approaches. Vertical bars indicate putative ecotype (PE) demarcation from Ecotype Simulation analysis. BAC clone sequences have a B in the clone name following the temperature designation (i.e., 60 or 65 representing clones from either the 60^o^C or 65^o^C sites). Ecotypes containing a mixture of BACs and PCR clones are boxed.Reference genome indicated by the word ‘OSA’ in PE10; Genbank accession number CP000239.

**Supplementary Figure 7.** (A) Bootstrapped, neighbor-joining phylogenetic tree for B´-like *Synechococcus* *rbsK* sequences obtained from a Mushroom Spring 60^o^C mat sample using either PCR- (grey colored sequences) or BAC-based cloning approaches. Vertical bars indicate putative ecotype (PE) demarcation from Ecotype Simulation analysis. BAC clone sequences have a B in the clone name following the temperature designation (i.e., 60 for the 60^o^C site). Ecotypes containing a mixture of BACs and PCR clones are boxed. BACs are color coded according to (B) missing loci downstream of the 16S rRNA (Blue = missing *sufB, argD,* and *apcAB*; Black = missing *accC, sufB, argD,* and *apcAB*; Red = missing *ispE, accC, sufB, argD,* and *apcAB*). Green BACs contained all genes downstream of the 16S rRNA locus. Position of mobile element denoted by yellow star; also see Supplementary Figures 1 and 5. Reference genome indicated by ‘OSB´ in PE1 (a green highlighted sequence).

**Supplementary Figure 8.** Phylogenetic incongruency. Lack of phylogenetic congruence and movement of recombinants among UPGMA phylogenies within the *rbsK* gene of A-like *Synechococcus* 60^o^C BAC clones constructed from (A) non-recombinant regions and (B) recombinant region 207-359 of the sequence data.

**Supplementary Figure 9.** Phylogenetic incongruency. Lack of phylogenetic congruence and movement of recombinants among UPGMA phylogenies within the *rbsK* gene of B´-like *Synechococcus* 60^o^C BAC clones constructed from (A) non-recombinant regions and (B) recombinant region 636-1085 of the sequence data.

**Supplementary Figure 10.** Multi-locus sequence analysis of 4 loci from B΄-like *Synechococcus* BACs illustrating single-nucleotide polymorphism analysis (open and closed stars), Ecotype Simulation analysis of the Maximum Likelihood concatenated phylogeny and corresponding single-locus trees. STs of the same PE are color coded the same in all phylogenies according to concatenated phylogeny and are indicated next to cluster with a bracket in all trees. The number of variants comprising dominant and subdominant STs is indicated in parenthesis following the ST designation. Open and closed stars are colored coded according to gene (see inset); stars representing sets of SNPs inferring a recombination event and supported by Clonal Frame analysis are ‘closed’ stars. SNPs only identified in SNP analysis are identified by open stars. Non-syntenous STs are shaded in grey and STs that contained a combination of sequences that were syntenous and non-syntenous are indicated by an asterisk; syntenous STs are not shaded and are not annotated by an asterisk. *Synechococcus* strain B΄ reference genome represented by ST44.

**Supplementary Figure 11.** eBURST population snapshot of B´-like *Synechococcus* BACs showing stringent (enclosed by solid lines) and relaxed (enclosed by dashed line) clonal complexes with PE demarcation with Ecotype Simulation analysis overlaid. Different colors represent distinct PEs (corresponding to Supplementary Figure 10). Sequence types (represented by numbers) not bounded by a colored area belong to PEs demarcated from a single sequence with a unique sequence type. *Synechococcus* strain B΄ reference genome represented by ST44.

**Supplementary Figure 12.** Single-nucleotide polymorphism patterning of variants for B´-like *Synechococcus* BACs. Figures show single-locus variants (blue), putative-ecotype variants (red), or both (purple) surrounding (A) dominant variant)-ST1 in PE-B1 and clonal complex B4-I, (B) dominant variant-ST6 in PE-B6 and clonal complex B4-II, and (C) dominant variant-ST5 in PE-B16 and clonal complex B4-III defined by Ecotype Simulation and eBURST analysis of 4 loci.

**Supplementary Figure 13.** of SNP patterns at all MLSA loci for all variants within PEs of the top, middle and bottom clades (top to bottom) of the B´-like *Synechococcus* phylogeny shown in Supplementary Figure 10 relative to the most dominant variant of each clade (A-C; top, D-F; middle and G-I; bottom).

**Supplementary Figure 14.** Lower resolution maximum likelihood single-locus trees for A-like *Synechococcus* BACs for *rbsK, CHP, lepB, dnaG, aroA, hisF* and *PK* with the 7-locus concatenated tree for reference. STs of the same PE are color coded the same in all phylogenies according to color coding in the concatenated tree and PE demarcations are indicated next to cluster with a bracket in all trees. *Synechococcus* strain A reference genome represented by ST10 in PEA13 of the concatenated tree.

**Supplementary Figure 15.** Ecotype Simulation analysis of the Maximum Likelihood concatenated phylogeny based on 49 sequences representing 5 loci from A-like *Synechococcus* BACs, and corresponding single-locus phylogenies. STs in the same PE are color coded the same in all phylogenies according to the concatenated phylogeny and are indicated next to cluster with a bracket in all trees. Dominant and subdominant STs are indicated by a small bracket enclosing the sequences followed by ST designation. PEs are color coded to match Supplementary Figure 17A. *Synechococcus* strain A reference genome represented by ST25 annotated ‘OSA’ in PE1 of the concatenated phylogeny.

**Supplementary Figure 16.** Ecotype Simulation analysis of the Maximum Likelihood concatenated phylogeny based on 145 sequences representing 5 loci from A-like *Synechococcus* sp. BACs;. STs are color coded by temperature sample (60^o^C = blue, 65^o^C = red) and are indicated next to cluster with a bracket in all trees. Please note that STs do not equate to STs in Figure 2 main text or Supplementary Figure 11; all analyses were completely separate. STs do however correlate with STs in Supplementary Figure 18B. Shading indicates examples of ST(s) that were recombinant (red), sharing evidence of the same recombination event as the red-shaded recombinant (light pink) or sharing partial evidence of the same recombination event as the red-shaded recombinant (fuschia). Grey shaded sequence is one of the inferred parents of red-shaded recombinant. Single and double asterisks indicate sub-dominant and dominant variant STs respectively.

**Supplementary Figure 17.** eBURST population snapshots for the 5-locus study of 49 A-like *Synechococcus* BACs. Clonal complexes are enclosed by black lines. In (A), PE demarcation from Ecotype Simulation analysis is overlaid, using different colors to represent distinct PEs. STs are represented by numbers and those shaded in grey belong to PEs demarcated from a single sequence with a unique ST. In (B), STs have been colored according to sample site of recovery; 60^o^C (blue) or 65^o^C (red). Those sequence types with a combination of sequences from both samples are red and blue. Temperature specific PEs are indicated by an asterisk in (A).

**Supplementary Figure 18.** eBURST population snapshots for the 5-locus study of 145 A-like *Synechococcus* BACs. Clonal complexes are enclosed by black lines. In (A), PE demarcation from Ecotype Simulation analysis is overlaid, using different colors to represent distinct PEs. STs are represented by numbers and those shaded in grey belong to PEs demarcated from a single sequence with a unique ST. In (B), STs have been colored according to sample site of recovery; 60^o^C (blue) or 65^o^C (red). Those sequence types with a combination of sequences from both samples are red and blue.

**Supplementary Figure 19**. A-like *Synechococcus* phylogeny based on maximum likelihood analysis of *rbsK* pyrosequences. Putative ecotypes (PEs) demarcated by Ecotype Simulation are indicated by brackets or vertical bars adjacent to each tree. STs within PEs are colored according to the MLSA phylogeny shown in main-text Figure 2A.

**Supplementary Figure 20.** Maximum Likelihood analysis of 7-locus concatenated sequences from A-like *Synechococcus* BAC and genomes of isolates representative of *psaA* PEs A6 and A14. phylogeny . Putative ecotypes (PEs) demarcated by Ecotype Simulation are indicated by unique colors and correspond to main text Figure 2. (also refer to Supplementary Table 25).

**Supplementary Figure 21.** Comparison of single nucleotide polymorphism patterns at all multi-locus sequence analysis loci for all variants within putative ecotypes of the top (PEs 2, 3 and 4), middle (PEs 1, 5, 6, 7 and 8) and bottom (PEs (9-13) clades of the A-like *Synechococcus* phylogeny shown in main-text Figure 2A relative to the most dominant variant (STs 1, 7 and 35) of each clade (A-C; middle clade, D-F; top clade and G-I; bottom clade). STs correspond with STs in Figure 2A.

**Supplementary Figure 22.** eBURST population snapshot of A-like *Synechococcus* BACs showing the single-locus connections between all STs regardless of predicted ancestor consensus sequence. Single locus variant connection between ST7/ST5 and ST7/ST6 is shaded in grey. Clonal complexes outlined in black (also see main-text Figure 4).

## Supplementary Tables (see Separate PDF Supplementary Materials Tables File).

**Supplementary Table 1**. Characteristics of loci used in analysis of A-like *Synechococcus* mat populations.

**Supplementary Table 2**. Characteristics of loci used in MSLA analysis of B´-like *Synechococcus* populations.

**Supplementary Table 3.** Percent recruitment by clusters defined in 2-12 kbp metagenome assembly analyses and BAC libraries (60^o^C and 65^o^C combined).

**Supplementary Table 4**. Comparison of BAC and small-insert metagenomic library compositions and synteny with reference genomes.

**Supplementary Table 5.** Distributions of mate-pair types of jointly-recruited end sequences of randomly selected and cyanobacterial (cyano) BAC clones.

**Supplementary Table 6**. Locus distribution of *Synechococcus* A-like BACs based on PCR amplification.

**Supplementary Table 7**. Locus distribution on *Synechococcus* B´-like BACs based on PCR amplification.

**Supplementary Table 8.** Allelic profiles generated from analysis of single nucleotide polymorphisms in the A-like *Synechococcus* BACs for protein-encoding sequence datasets of 7 loci.

**Supplementary Table 9.** Allelic profiles generated from analysis of single nucleotide polymorphisms in the B´-like *Synechococcus* BACs for protein-encoding sequence datasets of 4 loci.

**Supplementary Table 10.** Analysis of recombination signals in A-like *Synechococcus* BAC sequences in three

studies (MLSA7, MLSA5-49 sequences and MLSA5-145 sequences).

**Supplementary Table 11**. Analysis of recombination signals in B´-like *Synechococcus* BAC sequences.

**Supplementary Table 12**. P-values for RDP4 analysis of recombinants for A-like *Synechococcus* BACs. Corresponds to Supplementary Table 10.

**Supplementary Table 13**. P-values for RDP4 analysis of recombinants for B´-like *Synechococcus* BACs. Corresponds to Supplementary Table 11.

**Supplementary Table 14.** Sequence datasets used to infer between A- and B´-like *Synechococcus* BAC recombinants (R) using RDP4.

**Supplementary Table 15.** Linkage disequilibrium and per site rho and theta results from analysis of concatenated sequence data sets using LIAN, LDHat and Clonal Frame analysis.

**Supplementary Table 16.** Ecotype Simulation and eBURST output for B´-like *Synechococcus* BACs.

**Supplementary Table 17.** Comparison of singleton STs surrounding dominant variants of PEs and consensus sequences of clonal complexes observed in 4-locus MLSA of B´-like *Synechococcus* BACs. STs common to a PE and a corresponding clonal complex are highlighted in grey.

**Supplementary Table 18.** eBURST analysis of clonal complexes for the 4-locus MLSA of B´-like *Synechococcus* BACs. The number of BACs within STs is in parentheses when greater than 1. Superscripts next to the allele number denote number of nucleotide differences compared to the consensus sequence.

**Supplementary Table 19.** Allelic profiles generated from analysis of single nucleotide polymorphisms in 49 A-like *Synechococcus* BACs for protein-encoding sequence datasets of 5 loci.

**Supplementary Table 20**. Allelic profiles generated from analysis of single nucleotide polymorphisms in 145 A-like *Synechococcus* BACs for protein-encoding sequence datasets of 5 loci.

**Supplementary Table 21.** Ecotype Simulation and eBURST output for A-like *Synechococcus* BACs, 5-locus study of 49 sequences.

**Supplementary Table 22.** eBURST analysis of clonal complexes for the 5-locus MLSA of A-like *Synechococcus* BACs (49 sequences). The number of BACs within an ST is in parentheses when greater than 1. Superscripts next to the allele number denote number of nucleotide differences compared to the consensus sequence. Corresponding to Supplementary Figure 17.

**Supplementary Table 23.** Comparison of singleton STs surrounding dominant variants of PEs and consensus sequences of clonal complexes observed in 7-locus MLSA of A-like *Synechococcus* BACs. STs common to a PE and a corresponding clonal complex are highlighted in bold.

**Supplementary Table 24.** Comparison of singleton STs surrounding dominant variants of PEs and consensus sequences of clonal complexes observed in 5-locus MLSA of A-like *Synechococcus* BACs (49 sequences). STs common to a PE and a corresponding clonal complex are highlighted in bold. Note: STs designated in the A-like *Synechococcus* population 5 locus MLSA *do not* correspond to the STs in the 7 locus MLSA.

**Supplementary Table 25.** Pairwise percent nucleotide identity comparisons among A-like *Synechococcus* isolate genomes (see ref ([15](#_ENREF_15)) for details). Above the diagonal is the percent nucleotide identity for each pair of genomes calculated from an 1136 orthologous gene concatenation. Below the diagonal is the percent nucleotide identity for each pair of genomes calculated from the whole genome using reciprocal best hits. Bold text indicates within-PE comparisons.

**References**

1. Bhaya D, Grossman AR, Steunou AS, Khuri N, Cohan FM, Hamamura N, et al. Population level functional diversity in a microbial community revealed by comparative genomic and metagenomic analyses. ISME J. 2007;1(8):703-13.

2. Melendrez MC, Lange RK, Cohan FM, Ward DM. Influence of molecular resolution on sequence-based discovery of ecological diversity among *Synechococcus* populations in an alkaline siliceous hot spring microbial mat. Appl Environ Microbiol. 2011;77(4):1359-67.

3. Klatt CG, Wood JM, Rusch DB, Bateson MM, Hamamura N, Heidelberg JF, et al. Community ecology of hot spring cyanobacterial mats: predominant populations and their functional potential. ISME J. 2011;5(8):1262-78.

4. Ward DM, Bateson MM, Ferris MJ, Kühl M, Wieland A, Koeppel A, et al. Cyanobacterial ecotypes in the microbial mat community of Mushroom Spring (Yellowstone National Park, Wyoming) as species-like units linking microbial community composition, structure and function. Philosophical transactions of the Roy Soc of Lond Ser B, Biol Sci. 2006;361(1475):1997-2008.

5. Liles MR, Williamson LL, Rodbumrer J, Torsvik V, Goodman RM, Handelsman J. Recovery, purification, and cloning of high-molecular-weight DNA from soil microorganisms. Appl Environ Microbiol. 2008;74(10):3302-5.

6. Tao Q, Wang A, Zhang HB. One large-insert plant-transformation-competent BIBAC library and three BAC libraries of Japonica rice for genome research in rice and other grasses. Theor Appl Genet. 2002;105(6-7):1058-66.

7. Bertani G. Studies on lysogenesis. I. The mode of phage liberation by lysogenic *Escherichia coli.* J Bacteriol. 1951;62(3):293-300.

8. Papke RT, Ramsing NB, Bateson MM, Ward DM. Geographical isolation in hot spring cyanobacteria. Environ Microbiol. 2003;5(8):650-9.

9. Rusch DB, Halpern AL, Sutton G, Heidelberg KB, Williamson S, Yooseph S, et al. The Sorcerer II global ocean sampling expedition: Northwest Atlantic through Eastern Tropical Pacific. PLoS Biol. 2007;5(3):398-431.

10. Martin DP, Lemey P, Lott M, Moulton V, Posada D, Lefeuvre P. RDP3: a flexible and fast computer program for analyzing recombination. Bioinformatics. 2010;26(19):2462-3.

11. Edgar RC. MUSCLE: a multiple sequence alignment method with reduced time and space complexity. BMC Bioinformatics. 2004;5:113.

12. Haubold B, Hudson RR. LIAN 3.0: detecting linkage disequilibrium in multilocus data. Linkage Analysis. Bioinformatics. 2000;16(9):847-8.

13. Nowack S, Olsen MT, Schaible G, Becraft ED, Shen G, Klapper I, et al. The molecular dimension of microbial species: 2. *Synechococcus* isolates representative of putative ecotypes inhabiting different depths in the Mushroom Spring microbial mat exhibit different adaptive and acclimative responses to light. . Front. Microbiol. 2015;6:626.

14. Becraft ED, Wood JM, Rusch DB, Kuhl M, Jensen SI, Bryant DA, et al. The molecular dimension of microbial species: 1. Ecological distinctions among, and homogeneity within, putative ecotypes of *Synechococcus* inhabiting the cyanobacterial mat of Mushroom Spring, Yellowstone National Park. Front. Microbiol. 2015;6:590.

15. Olsen MT, Nowack S, Wood JM, Becraft ED, LaButti K, Lipzen A, et al. The molecular dimension of microbial species: 3. Comparative genomics of *Synechoccocus* isolates with different light responses and *in situ* diel transcription patterns of associated putative ecotypes in the Mushroom Spring microbial mat. Front. Microbiol. 2015;6:604.

16. Markowitz VM, Mavromatis K, Ivanova NN, Chen IM, Chu K, Kyrpides NC. IMG ER: a system for microbial genome annotation expert review and curation. Bioinformatics. 2009;25(17):2271-8.

17. Markowitz VM, Szeto E, Palaniappan K, Grechkin Y, Chu K, Chen IM, et al. The integrated microbial genomes (IMG) system in 2007: data content and analysis tool extensions. Nucleic Acids Res. 2008;36(Database issue):D528-33.
